# Atezolizumab–bevacizumab with or without cisplatin-based HAIC for unresectable HCC: A randomized phase II trial

**DOI:** 10.1016/j.jhepr.2026.102002

**Published:** 2026-08-29

**Authors:** Yasuto Takeuchi, Masahiko Sue, Nozomi Miyake, Takuya Adachi, Hideki Onishi, Miwa Kawanaka, Akinobu Takaki, Kazuhiro Nouso, Kazuya Kariyama, Tetsuya Yasunaka, Kenji Kuwaki, Junichi Toshimori, Shota Iwado, Akio Moriya, Hiroaki Hagihara, Hiroyuki Takabatake, Masahiro Sakata, Takeshi Hatanaka, Shigeo Shimose, Takumi Kawaguchi, Motoyuki Otsuka

**Affiliations:** 1 Department of Gastroenterology, Okayama University Hospital, Japan; 2 Department of Gastroenterology, Okayama City Hospital, Japan; 3 Department of Gastroenterology, Fukuyama City Hospital, Japan; 4 Department of Gastroenterology, Okayama Saiseikai General Hospital, Japan; 5 Department of Gastroenterology, Japanese Red Cross Okayama Hospital, Japan; 6 Department of Gastroenterology, Hiroshima City Hospital, Japan; 7 Department of Gastroenterology, Mitoyo General Hospital, Japan; 8 Department of Gastroenterology, Sumitomo Besshi Hospital, Japan; 9 Department of Gastroenterology, Kurashiki Central Hospital, Japan; 10 Department of Gastroenterology, Fukuyama Medical Center, Japan; 11 Department of Gastroenterology, Gunma Saiseikai Maebashi Hospital, Maebashi, Japan; 12 Division of Gastroenterology, Department of Medicine, Kurume University School of Medicine, Kurume, Japan

**Keywords:** Hepatocellular carcinoma, Atezolizumab plus bevacizumab, Cisplatin hepatic arterial infusion chemotherapy

## Abstract

**Background & Aims:**

Atezolizumab plus bevacizumab (ATZ/BEV) is the global standard first-line treatment for unresectable hepatocellular carcinoma (HCC). However, the objective response rate (ORR) is approximately 30%. Hepatic arterial infusion chemotherapy (HAIC) with cisplatin may enhance its efficacy through locoregional cytoreduction and immunological modulation. We conducted a randomized phase II trial to evaluate whether sequential cisplatin HAIC plus ATZ/BEV (CDDP+ATZ/BEV) improves clinical outcomes compared to ATZ/BEV alone.

**Methods:**

In this multicenter, open-label trial, patients with unresectable HCC were randomized 1:1 to receive cisplatin HAIC within 8 weeks before initiation of ATZ/BEV or ATZ/BEV alone. The primary endpoint was ORR.

**Results:**

Between December 2021 and December 2024, 70 patients were enrolled in this study (CDDP+ATZ/BEV, n = 35; ATZ/BEV, n = 35). The primary endpoint, ORR, was numerically higher in the CDDP+ATZ/BEV group than in the ATZ/BEV group but did not reach statistical significance (45.7% *vs.* 28.6%; *p =* 0.095). Median progression-free survival (PFS) was longer in the CDDP+ATZ/BEV group (8.3 *vs.* 6.6 months; HR 0.53; *p =* 0.036), and median duration of response was not reached *vs.* 4.6 months (HR 0.4; *p =* 0.11). The median overall survival (OS) was 28.1 *vs.* 31.0 months (HR 0.95; *p =* 0.88). Grade 3–4 treatment-related adverse events occurred in 20.0% and 22.9% of patients, respectively.

**Conclusions:**

The primary endpoint of ORR was not met. However, exploratory analyses demonstrated longer PFS without additional safety concerns. Further evaluation in phase III trials is warranted.

**Impact and implications:**

This multicenter randomized phase II trial provides evidence that sequential cisplatin hepatic arterial infusion chemotherapy followed by atezolizumab–bevacizumab may prolong disease control without increasing toxicity in patients with unresectable hepatocellular carcinoma. Although the primary endpoint was not met and the progression-free survival benefit was observed in exploratory analyses, these findings support further investigation of this strategy in phase III trials and provide a rationale for evaluating liver-directed combination approaches to optimize first-line immunotherapy.

**Clinical trial number:**

Japan Registry of Clinical Trials (jRCTs061210065).

## Highlights

• This multicenter randomized phase II trial evaluated sequential cisplatin HAIC followed by atezolizumab–bevacizumab in unresectable HCC.
• The primary endpoint of objective response rate was numerically higher with cisplatin HAIC but did not reach statistical significance.
• Exploratory analyses demonstrated significantly longer progression-free survival without increased treatment-related toxicity.
• These findings support further evaluation of sequential cisplatin HAIC plus atezolizumab–bevacizumab in a phase III trial.

## Graphical abstract

**Figure:**
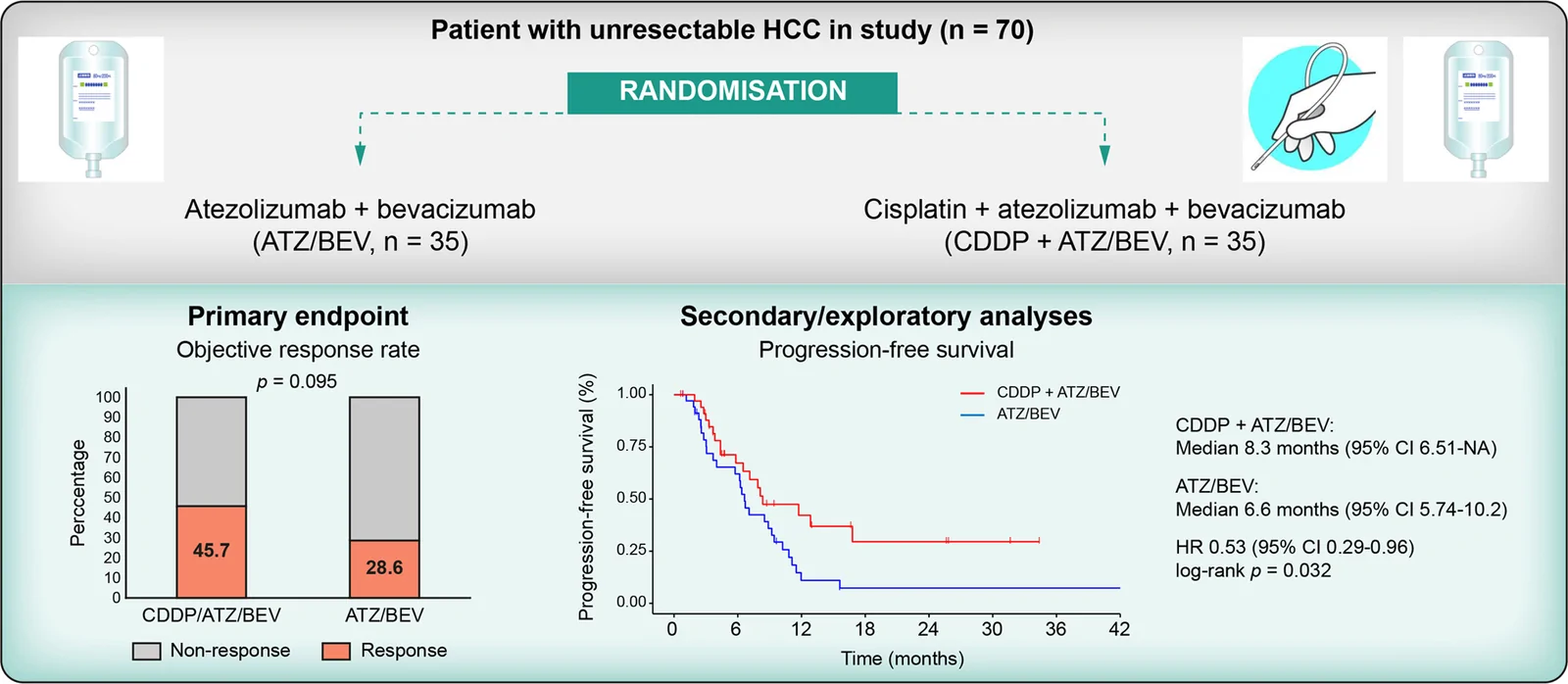

## Introduction

Hepatocellular carcinoma (HCC) remains a leading cause of cancer-related mortality worldwide, especially in cases of unresectable disease where the prognosis is poor despite advances in systemic therapies. The combination of the immune checkpoint inhibitor atezolizumab and the VEGF-targeting agent bevacizumab (ATZ/BEV) has become the global standard first-line treatment, following the compelling survival benefits demonstrated in the IMbrave150 trial.^1^ Nevertheless, the objective response rate (ORR) with ATZ/BEV remains at approximately 30%, indicating that most patients fail to achieve significant tumor shrinkage.

Cisplatin-based hepatic arterial infusion chemotherapy (HAIC) delivers high intrahepatic drug concentrations with meaningful tumor responses and limited systemic toxicity.[2], [3], [4] Beyond local cytoreduction, liver-directed therapies, such as HAIC or transarterial chemoembolization (TACE), may also modulate systemic antitumor immunity. Preclinical and translational studies have suggested that locoregional tumor destruction can release tumor antigens, induce immunogenic cell death, and enhance T-cell priming.^5^ Furthermore, combining these liver-directed therapies with VEGF inhibitors may normalize the tumor vasculature, reduce immunosuppressive myeloid cells, and facilitate immune infiltration, creating a more permissive tumor microenvironment for immune checkpoint blockade.^6^^,^^7^ Collectively, these findings provide a strong biological rationale for combining HAIC with ATZ/BEV, aiming to augment both local control and the systemic immune response.

Building on this concept, we designed a multicenter, randomized, phase II trial to test whether sequential cisplatin HAIC followed by ATZ/BEV could improve outcomes compared to that with ATZ/BEV alone in patients with unresectable HCC. We hypothesized that the addition of HAIC would increase the tumor response through both direct cytoreduction and immunologic modulation, thereby prolonging disease control without compromising safety.

## Patients and methods

### Study design and participants

Eligible patients were adults (aged ≥18 years) with a confirmed diagnosis of unresectable HCC. Unresectability was determined by a multidisciplinary tumor board based on tumor extent, liver function, and patient performance status. All patients were required to have at least one measurable intrahepatic lesion according to RECIST version 1.1^8^ because it has been widely adopted in contemporary clinical trials of immune checkpoint inhibitor-based systemic therapies for HCC and facilitates comparison with established efficacy data from pivotal studies.

Additional inclusion criteria were an Eastern Cooperative Oncology Group (ECOG) performance status of 0 or 1 and preserved liver function defined as a Child–Pugh score of 5–7. Patients were required to be naïve to cisplatin-based HAIC.

Key exclusion criteria included a history of two or more prior TACE procedures, absence of intrahepatic target lesions, and known hypersensitivity to cisplatin or to either component of atezolizumab plus bevacizumab therapy. Patients were also excluded if they had contraindications to immune checkpoint inhibitors or molecular targeted agents, including but not limited to active autoimmune disease requiring systemic immunosuppression. Additional exclusion criteria were the presence of severe cardiovascular disease and impaired renal function, defined as an estimated glomerular filtration rate (eGFR) <30 ml/min/1.73 m^2^ (Fig. 1).

**Fig. 1:**
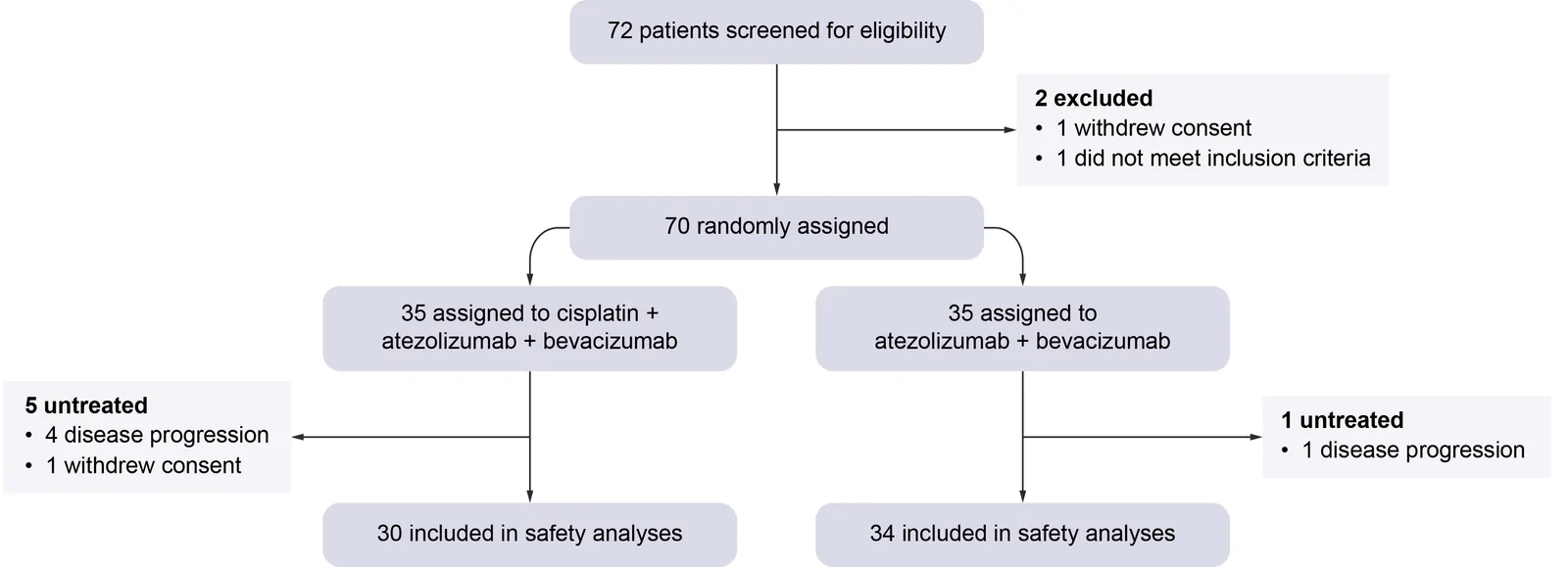
CONSORT flow diagram of patient enrollment, randomization, treatment allocation, and analysis populations. A total of 70 patients were randomized to receive either cisplatin-based hepatic arterial infusion chemotherapy followed by atezolizumab + bevacizumab (n = 35) or atezolizumab plus bevacizumab alone (n = 35). All randomized patients were included in the intention-to-treat efficacy analyses. Patients who received the assigned treatment were included in the safety and per-protocol analyses.

The study was conducted in accordance with the principles of the Declaration of Helsinki and the International Council for Harmonization Good Clinical Practice guidelines. The study protocol was reviewed and approved by the Okayama University Certified Review Board (approval number: CRB21-006). Written informed consent was obtained from all participants prior to enrollment.

### Randomization and procedures

Patients were randomly assigned (1:1) using a REDCap (Research Electronic Data Capture) system,^9^^,^^10^ stratified by previous TACE treatment (yes *vs.* no) and the presence of macrovascular invasion. The trial was an open label.

In the experimental arm, patients received a single infusion of cisplatin (65 mg/m^2^) as HAIC within 8 weeks before initiation of atezolizumab plus bevacizumab. No additional HAIC cycles were planned or permitted after initiation of systemic therapy. The control arm was administered ATZ/BEV at identical doses and schedules. HAIC administration was mandatory prior to ATZ/BEV administration in the experimental arm. For cisplatin hepatic artery infusion, the site was the proper hepatic artery when tumors were present in both lobes of the liver, and the right or left hepatic artery when tumors were present in only one lobe. If patients experienced HAIC-related adverse events, subsequent ATZ/BEV treatment was permitted, provided that the predefined discontinuation criteria were not met.

Treatment was continued until disease progression, unacceptable toxicity, or withdrawal of consent. Treatment beyond progression was permitted when the patient was receiving clinical benefit, at the investigator’s discretion. No dose escalation or reduction of ATZ or BEV was allowed. However, treatment delays with ATZ, BEV, or both were permitted in the event of adverse events (AEs). Intervention with local therapy during treatment was not permitted; however, after progression, intervention with local therapy was permitted at the discretion of the attending physician.

Tumor assessments were performed at baseline, at weeks 9–12, and every 12 weeks thereafter, using contrast-enhanced computed tomography or magnetic resonance imaging and covering all known or suspected disease sites. Baseline tumor evaluation was performed within 28 days before randomization. Subsequent assessments were scheduled 9–12 weeks (±1 week) after the initiation of ATZ/BEV and every 12 weeks (±1 week) thereafter, until progressive disease or treatment discontinuation, whichever occurred later. Changes in tumor measurements and treatment responses were assessed by the investigators according to RECIST version 1.1, which guides decisions regarding treatment continuation.

### Outcomes

The primary endpoint was the objective response rate (ORR), defined as the proportion of patients achieving the best overall response of complete response or partial response according to RECIST version 1.1.

Secondary endpoints included progression-free survival (PFS), disease control rate, duration of response (DoR), overall survival (OS), and safety. PFS and OS were calculated from the time of randomization. PFS was defined as the time from randomization to disease progression or death from any cause, whichever occurred first, and OS as the time from randomization to death from any cause.

For the safety assessment, all AEs were monitored throughout the treatment period and for 48 weeks after treatment initiation, as specified in the protocol, even if the treatment was discontinued earlier. AEs were defined and graded according to the National Cancer Institute CTCAE version 5.0.^11^

The effect of baseline demographic and clinical characteristics on PFS was evaluated in pre-specified subgroup analyses of all randomly assigned patients, excluding those with an undetermined best overall response. The subgroups included standard demographic variables and stratification factors: age, sex, Child–Pugh score, etiology, presence of macrovascular invasion, extrahepatic spread, baseline alpha-fetoprotein concentration, and baseline Barcelona Clinic Liver Cancer (BCLC) stage.^12^

### Data collection

Study data were collected and managed using REDCap electronic data capture tools hosted at Okayama University Hospital, which is a secure web-based software platform designed to facilitate research data management, offering 1) an intuitive interface for validated data entry, 2) audit trails for tracking data manipulation and export, 3) automated export procedures for seamless transfer to common statistical packages, and 4) features for integration and interoperability with external data sources. The data cut-off date for the present analysis was March 31, 2026.

### Sample size and statistical analysis

During protocol development and prior to patient enrollment, the sample size calculation was revised based on updated efficacy results from the IMbrave150 trial. The original protocol assumed efficacy estimates derived from the initial IMbrave150 report; however, subsequently published updated data demonstrated improved treatment outcomes with atezolizumab plus bevacizumab. As a result, the expected response rate in the control arm was revised upward, and the sample size calculation was recalculated accordingly, resulting in a reduction in the required sample size. This amendment was completed and approved before study initiation.

All eligible patients registered in this study were included in the analysis. Patient demographics, baseline clinical characteristics, and tumor status were summarized as aggregated data. Continuous variables were presented as medians with interquartile ranges (25th–75th percentiles). The primary efficacy analyses were conducted in the intention-to-treat (ITT) population, defined as all randomized patients according to their assigned treatment group, regardless of treatment initiation or protocol adherence. Safety analyses were performed in all patients who received at least one dose of study treatment (safety population). Additional per-protocol analyses were performed as sensitivity analyses in patients who received the assigned treatment.

Differences in objective response rate between the treatment groups were assessed using a two-sided Fisher's exact test. PFS, DoR, and OS were estimated using the Kaplan–Meier method, and hazard ratios (HRs) with 95% CIs were calculated using Cox proportional hazards models. Safety was assessed in all randomly assigned patients who enrolled in the study.

All statistical analyses were performed using the R software version 4.4.0. All tests were two-sided, and *p* values <0.05 were considered statistically significant.

## Results

### Patient characteristics

Between December 21, 2021, and December 31, 2024, a total of 72 patients with unresectable HCC provided written informed consent and were screened for eligibility. Of these, one patient withdrew consent prior to enrollment, and one patient was excluded because the eligibility criteria were not met. The remaining 70 eligible patients were randomized in a 1:1 ratio to receive cisplatin-based HAIC plus atezolizumab and bevacizumab (CDDP+ATZ/BEV group, n = 35) or atezolizumab plus bevacizumab alone (ATZ/BEV group, n = 35). In the CDDP+ATZ/BEV group, one patient withdrew consent and four patients experienced rapid disease progression before initiation of the assigned treatment, resulting in 30 patients who actually received the study treatment. In the ATZ/BEV group, one patient was unable to initiate treatment because of disease progression prior to treatment start, leaving 34 patients who received the assigned therapy. Consequently, 70 patients were included in the ITT efficacy analyses according to their randomized treatment assignment. Among them, 30 patients in the CDDP+ATZ/BEV group and 34 patients in the ATZ/BEV group received the assigned treatment and were included in the safety and per-protocol analyses. There were no differences in baseline demographics and disease characteristics between the two groups (Table 1).

**Table 1:** Baseline patient demographics and disease characteristics.

	Cisplatin + atezolizumab + bevacizumab (n = 35)	Atezolizumab + bevacizumab (n = 35)	*p* value
**Patient demographics**			
Age, years	74 (73–79)	75 (63–77)	0.19
Sex			0.52
Male, n (%)	30 (85.7)	28 (80.0)	
Female, n (%)	5 (14.3)	7 (20.0)	
Etiology			0.45
Viral, n (%)	13 (37.1)	10 (28.6)	
Non-viral, n (%)	22 (62.9)	25 (71.4)	
ECOG PS			0.23
0, n (%)	26 (74.3)	30 (85.7)	
1, n (%)	9 (25.7)	5 (14.3)	

**Liver function**			
Child-Pugh score			0.81
5, n (%)	21 (60.0)	22 (62.9)	
6, n (%)	14 (40.0)	13 (37.1)	
ALBI score	-2.30 (-2.45– -2.09)	-2.39 (-2.67– -2.17)	0.40
Creatinine	0.78 (0.68–0.96)	0.75 (0.65–0.90)	0.20

**Tumor characteristics**			
BCLC stage			0.18
0/A	1 (2.9)	5 (14.3)	
B	15 (42.9)	16 (45.7)	
C	19 (54.2)	14 (40.0)	
Maximum intrahepatic tumor diameter (mm)	51 (25–100)	30 (15–100)	0.26
Number of nodules			0.33
1, n (%)	7 (20.0)	7 (20.0)	
2-3, n (%)	7 (20.0)	12 (34.3)	
≥ 4, n (%)	21 (60.0)	16 (45.7)	
Macrovascular invasion, n (%)	12 (34.3)	10 (28.6)	0.61
Extrahepatic spread, n (%)	11 (31.4)	7 (20.0)	0.27
AFP (ng/ml)	20 (5.9–508.3)	138 (10.1–978)	0.12
AFP-L3 (%)	4.7 (0.5–23.5)	23.3 (0.9–51.6)	0.52
DCP (mAU/ml)	1,377 (73–17,486)	517 (64–4,238)	0.77

**Previous treatment**			
Previous TACE, n (%)	11 (31.4)	12 (34.3)	0.80
Previous systemic therapy, n (%)	2 (5.7)	0 (0.0)	0.15

**Treatment characteristics**			
Period until ATZ/BEV	5 (4–6)	—	—

Data are presented as medians (interquartile ranges) or n (%) unless otherwise indicated. AFP, alpha-fetoprotein; AFP-L3, Lens culinaris agglutinin-reactive alpha-fetoprotein isoform; ALBI, albumin–bilirubin; BCLC, Barcelona Clinic Liver Cancer; DCP, des-gamma-carboxy prothrombin; PS, performance status. *p* values were calculated using the Mann–Whitney U test for continuous variables and Pearson’s chi-square test for categorical variables.

The median age was 74 years in the CDDP+ATZ/BEV group and 75 years in the ATZ/BEV group. Most patients were male (85.7% in the CDDP+ATZ/BEV group *vs.* 80.0% in the ATZ/BEV group), and viral etiologies accounted for 37.1% and 28.6% of cases, respectively. The majority of the participants had an ECOG performance status of 0. Most patients had a Child–Pugh score of 5 (60.0% *vs.* 62.9%). The proportions of patients with BCLC stage C disease were 54.2% and 40.0%, respectively. The prevalence of macrovascular invasion and extrahepatic spread was comparable between the two groups (34.3% *vs.* 28.6% and 31.4% *vs.* 20.0%, respectively).

### Efficacy outcomes

The ORR was 45.7% in the CDDP+ATZ/BEV group and 28.6% in the ATZ/BEV group (*p =* 0.095). Complete responses occurred in 5.7% and 2.9% of patients, respectively, and the disease control rates were 74.3% and 68.6%, respectively (Table 2).

**Table 2:** Tumor response outcomes.

	Cisplatin + atezolizumab + bevacizumab (n = 35)	Atezolizumab + bevacizumab (n = 35)	*p* value
Objective response rate^a^, n (%)	16 (45.7)	10 (28.6)	0.095
Disease control rate^a^, n (%)	26 (74.3)	24 (68.6)	0.19
Best overall response			
Complete response, n (%)	2 (5.7)	1 (2.9)	
Partial response, n (%)	14 (40.0)	9 (25.7)	
Stable disease, n (%)	10 (28.6)	14 (40.0)	
Progressive disease, n (%)	3 (8.6)	8 (22.9)	
Not evaluable, n (%)	6 (17.1)	3 (8.5)	
Median time to response (IQR), months^b^	2.99 (2.00–4.89)	2.97 (2.14–5.55)	0.91

Between-group differences in ORR and DCR were assessed using Cochran–Mantel–Haenszel test, and time to response was compared using the Mann–Whitney *U* test.

a Patients who had confirmed complete response or partial response per RECIST (version 1.1).

b Assessed in patients with a confirmed partial or complete response.

For the key secondary endpoint, PFS, CDDP+ATZ/BEV demonstrated a statistically significant benefit over ATZ/BEV (median 8.3 *vs.* 6.6 months; HR 0.53, 95% CI 0.29–0.96; *p =* 0.036; Fig. 2A). Subgroup analyses are shown in Fig. 2B. HRs and 95% CIs for each subgroup are presented in the forest plot. Statistically significant differences were observed in the following subgroups: age ≥65 years, male sex, viral hepatitis, presence of macrovascular invasion, presence of extrahepatic spread, and AFP ≥400 ng/ml.

**Fig. 2:**
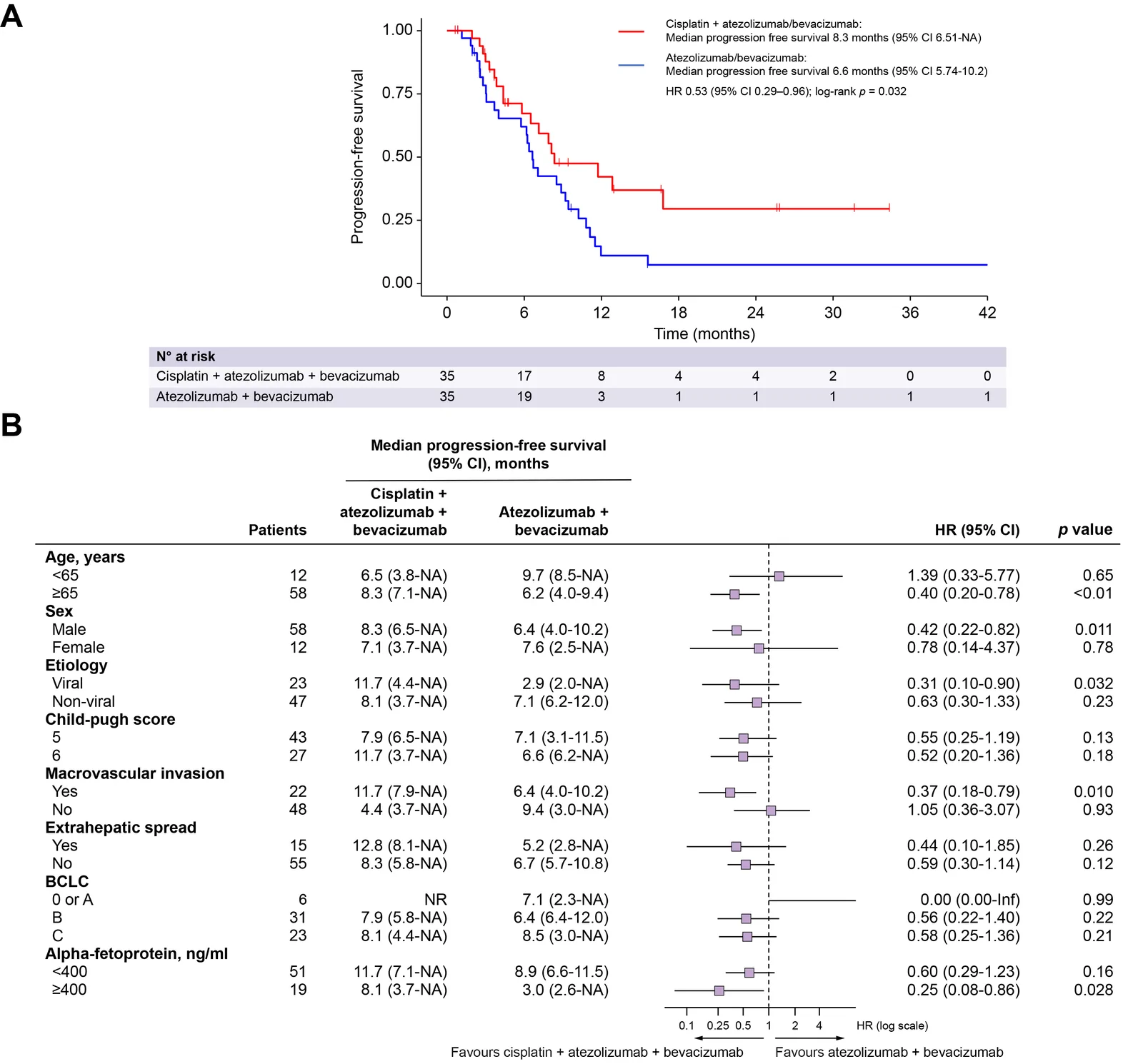
Progression-free survival and forest plot. (A) Kaplan–Meier estimates of progression-free survival for patients treated with cisplatin-based HAIC plus atezolizumab plus bevacizumab (n = 35) and those treated with atezolizumab plus bevacizumab alone (n = 35). Median progression-free survival was 8.3 months (95% CI 6.50–NA) in the cisplatin-based HAIC plus atezolizumab plus bevacizumab group *vs*. 6.6 months (95% CI 5.75–10.2) in the atezolizumab plus bevacizumab alone group (HR 0.53; 95% CI 0.29–0.96; *p* = 0.036). (B) Forest plot of prespecified subgroup analyses. Treatment benefit with cisplatin-based HAIC plus atezolizumab plus bevacizumab over atezolizumab plus bevacizumab was generally consistent across age, sex, etiology, presence of macrovascular invasion, baseline alpha-fetoprotein level, and Barcelona Clinic Liver Cancer stage. HRs are shown with 95% CIs. Numbers at risk and censoring marks are shown below the Kaplan–Meier plot. HAIC, hepatic arterial infusion chemotherapy; HR, hazard ratio; PFS, progression-free survival. PFS was estimated using the Kaplan–Meier method. Hazard ratios and 95% confidence intervals were estimated using Cox proportional hazards models, and between-group differences were assessed using the log-rank test. *p* values for interaction were obtained from Cox proportional hazards models including treatment-by-subgroup interaction terms.

In the exploratory subgroup analysis, the treatment effect on PFS according to macrovascular invasion status was assessed using an interaction test. Kaplan–Meier curves stratified by the presence or absence of macrovascular invasion are shown in Fig. S1. Although the estimated HR favored CDDP+ATZ/BEV more strongly in patients with macrovascular invasion, the interaction test did not demonstrate a statistically significant interaction between treatment effect and macrovascular invasion status (*p* for interaction = 0.084).

The median DoR was not reached (95% CI 7.89–NA) in the CDDP+ATZ/BEV group *vs.* 4.6 months (95% CI 3.22–NA) in the ATZ/BEV group. The HR for DoR was 0.40 (95% CI 0.13–1.22; *p =* 0.11) (Fig. 3). Following study treatment, two patients in the CDDP+ATZ/BEV group and one patient in the ATZ/BEV group underwent curative-intent treatment (surgical resection or local ablation).

**Fig. 3:**
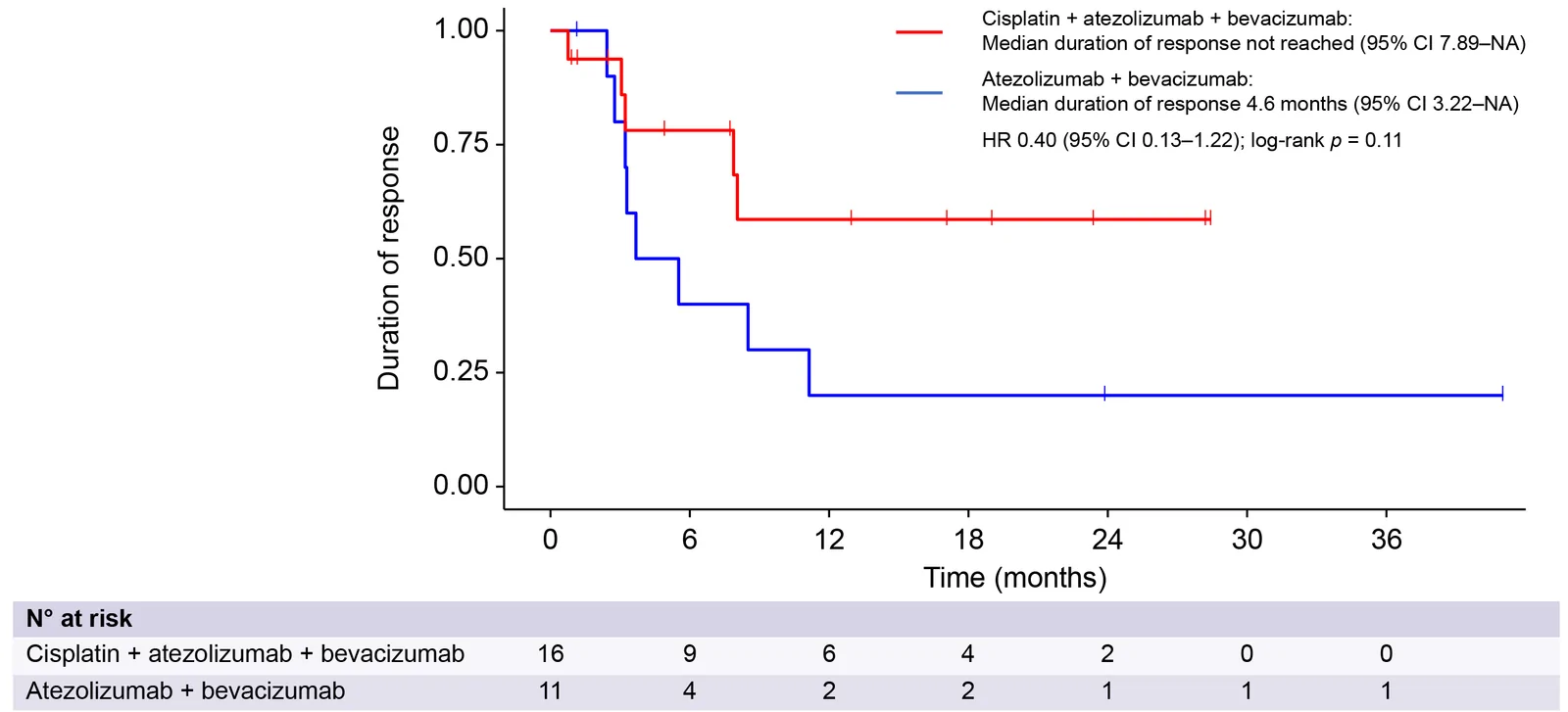
Duration of response among responders. Kaplan–Meier estimates of duration of response in patients who achieved complete or partial response. The median duration of response was not reached (95% CI 7.89–NA) in the cisplatin-based HAIC plus atezolizumab plus bevacizumab group *vs*. 4.6 months (95% CI 3.22–NA) in the atezolizumab + bevacizumab alone group (HR, 0.40; 95% CI 0.13–1.22; *p* = 0.11). The numbers at risk and the censoring marks are shown in the plot below. HAIC, hepatic arterial infusion chemotherapy; HR, hazard ratio. DoR was estimated using the Kaplan–Meier method. Hazard ratios and 95% confidence intervals were estimated using Cox proportional hazards models, and between-group differences were assessed using the log-rank test.

At a median follow-up of 16.7 months, the median OS was 28.1 months (95% CI 25.9–NA) in the CDDP+ATZ/BEV group *vs.* 31.0 months (95% CI 16.5–NA) in the ATZ/BEV group (HR 0.95, 95% CI 0.46–1.95; Fig. 4).

**Fig. 4:**
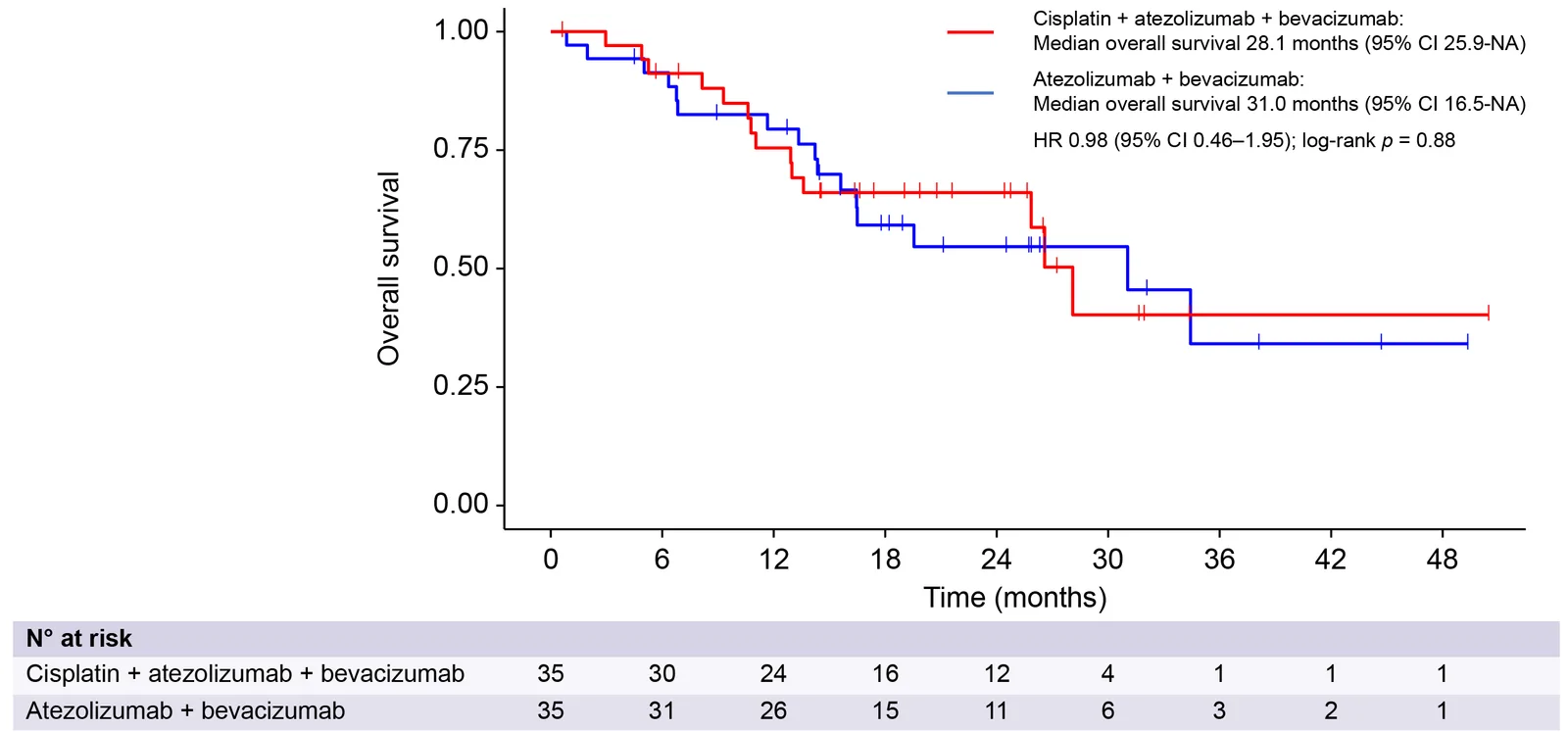
Overall survival in the intention-to-treat population. Kaplan–Meier estimates of overall survival in both treatment groups. At a median follow-up of 16.7 months, the median overall survival was 28.1 months (95% CI 25.9–NA) in the cisplatin-based HAIC plus atezolizumab plus bevacizumab group, compared with 31.0 months (95% CI 16.5–NA) in the atezolizumab + bevacizumab alone group (HR, 0.95; 95% CI 0.46–1.95). The numbers at risk and the censoring marks are shown in the plot below. HAIC, hepatic arterial infusion chemotherapy; HR, hazard ratio. OS was estimated using the Kaplan–Meier method. Hazard ratios and 95% confidence intervals were estimated using Cox proportional hazards models, and between-group differences were assessed using the log-rank test.

### Safety

Treatment-related AEs (TRAEs) were observed in 48.6% and 51.5% of patients in the CDDP+ATZ/BEV group and ATZ/BEV groups, respectively (Table 3). Grade 3–4 TRAEs were reported in 20.0% and 22.9% of patients, respectively. The most common AEs were hepatic dysfunction, proteinuria, and variceal bleeding. To date, no grade 5 TRAEs have been reported. The rate of treatment discontinuation due to TRAEs was comparable between groups (17.2% *vs.* 20.1%).

**Table 3:** Treatment-related adverse events in all treated patients.^a^

	Cisplatin + atezolizumab + bevacizumab (n = 35)		Atezolizumab + bevacizumab (n = 35)	
	Grade 1-2	Grade 3-4^b^	Grade 1-2	Grade 3-4^b^
Any treatment-related adverse event, n (%)	10 (28.6)	7 (20.0)	10 (28.6)	8 (22.9)
Treatment-related adverse event leading to discontinuation^c^, n (%)	1 (2.9)	5 (14.3)	2 (5.8)	5 (14.3)
Treatment-related adverse events				
Fever	1 (2.9)	0	5 (14.3)	0
Hepatic failure	0	1 (2.9)	3 (8.6)	1 (2.9)
Proteinuria	1 (2.9)	1 (2.9)	1 (2.9)	1 (2.9)
Edema	1 (2.9)	0	3 (8.6)	0
Variceal bleeding	1 (2.9)	1 (2.9)	0	2 (5.8)
AST increased	1 (2.9)	1 (2.9)	0	1 (2.9)
ALT increased	1 (2.9)	1 (2.9)	0	1 (2.9)
Hepatic encephalopathy	0	0	1 (2.9)	2 (5.8)
Hypertension	0	0	1 (2.9)	1 (2.9)
Joint pain	1 (2.9)	0	1 (2.9)	0
Neutropenia	0	1 (2.9)	0	1 (2.9)
Pneumonitis	1 (2.9)	1 (2.9)	0	0
Tumor hemorrhage	1 (2.9)	0	0	1 (2.9)
Colitis	0	1 (2.9)	0	0
Cytokine release syndrome	0	0	0	1 (2.9)
Decreased appetite	1 (2.9)	0	0	0
Diarrhea	1 (2.9)	0	0	0
Fatigue	0	0	1 (2.9)	0
Hyperglycemia	0	1 (2.9)	0	0
Hyperthyroidism	1 (2.9)	0	0	0
Hypoadrenocorticism	0	0	0	1 (2.9)
Hypothyroidism	0	0	1 (2.9)	0
Nausea	1 (2.9)	0	0	0
Platelet count decreased	0	1 (2.9)	0	0

Data are n (%). Adverse events were graded per CTCAE v5.0.

ALT, alanine aminotransferase; AST, aspartate aminotransferase.

a Includes patients who received at least one dose of the study treatment.

b No grade 5 treatment-related adverse events occurred in either treatment group.

c This category refers to adverse events leading to discontinuation of any drug in the regimen.

### Sensitivity analyses

Sensitivity analyses performed in the per-protocol population demonstrated findings generally consistent with those observed in the ITT population. The objective response rate remained higher in the CDDP+ATZ/BEV group, and treatment effects on PFS and OS were similar to those observed in the primary analyses. Detailed results are provided in Table S1 and Figs. S2 and S3.

## Discussion

In this multicenter, randomized, phase II trial, the addition of cisplatin-based HAIC prior to ATZ/BEV was compared with ATZ/BEV alone in patients with unresectable HCC. This randomized phase II trial did not meet its primary endpoint of ORR in the ITT population. However, exploratory analyses demonstrated longer PFS and DoR in the CDDP+ATZ/BEV arm. These findings should be interpreted cautiously given the negative primary endpoint and limited sample size. However, these findings suggest that the sequential administration of cisplatin HAIC followed by ATZ/BEV may provide clinically meaningful therapeutic advantages. Importantly, the findings of the per-protocol sensitivity analyses were generally consistent with those observed in the primary ITT analyses, supporting the robustness of the study findings.

The control arm outcomes were concordant with those reported in the IMbrave150 trial,^13^^,^^14^ in which ATZ/BEV achieved an ORR of approximately 30% and median PFS of 6.8 months. This external consistency underscores the validity of our study population and supports the interpretation that the improvements observed with CDDP+ATZ/BEV reflect a true treatment effect rather than a baseline imbalance.

Although the exploratory subgroup analyses suggested a greater treatment effect in patients with macrovascular invasion and extrahepatic spread, these observations were based on a limited number of patients and should be interpreted cautiously. The findings are consistent with previous reports demonstrating clinical activity of HAIC in patients with vascular invasion; however, the present study was not powered to establish differential treatment effects across subgroups. Therefore, these results should be considered hypothesis-generating and require confirmation in larger prospective studies.

Mechanistically, the benefits of HAIC before systemic therapy can be understood through two pathways. First, HAIC delivers high intrahepatic concentrations of cisplatin, achieving locoregional cytoreduction that translates into improved intrahepatic control, as shown in earlier HAIC studies.^3^^,^^4^ Second, the synergistic immunological mechanisms are plausible. Local tumor killing induces antigen release and promotes T-cell infiltration, which may augment checkpoint blockade efficacy.[15], [16], [17], [18], [19] Similar immune-enhancing effects have been reported following other locoregional therapies, such as TACE and radiofrequency ablation.[20], [21], [22] Notably, the observed efficacy in patients with macrovascular invasion and extrahepatic spread is unlikely to be explained solely by a potential abscopal-like effect. In addition to immune modulation, cisplatin HAIC has demonstrated favorable antitumor activity against aggressive intrahepatic disease, including tumors with portal vein invasion. Therefore, improved intrahepatic disease control together with potential immunological enhancement may have contributed to the observed clinical benefit.

The safety profile is also noteworthy. Both cisplatin and bevacizumab are associated with nephrotoxicity,[23], [24], [25] and their combination has raised concerns regarding other toxicities.[26], [27], [28], [29] However, in our trial, the incidence of renal AEs did not increase in the CDDP+ATZ/BEV arm, and the overall rates of grade 3–4 treatment-related AEs were comparable between the groups. These results suggest that delivering cisplatin via hepatic arterial infusion may reduce systemic exposure and mitigate nephrotoxicity.^30^^,^^31^ These findings suggest that this regimen is feasible and tolerable; however, confirmation in a larger cohort is required.

This study has some limitations. First, the relatively small sample size limits the precision of the estimates, which likely contributes to the statistically non-significant difference in ORR despite a clear numerical difference. Second, tumor responses were assessed by investigators without blinded independent central reviews, which may have introduced bias. Third, the OS data remain immature, and a longer follow-up period is needed to clarify whether the PFS and DoR benefits translate into an OS advantage. Although PFS has been proposed as a surrogate endpoint for OS in advanced HCC, the association is not uniform across all trials. Previous trial-level analyses have demonstrated a moderate correlation between PFS and OS,^32^ although longer follow-up is still required to determine whether the observed PFS benefit translates into improved OS. Another limitation of this study is the relatively low proportion of patients with BCLC stage C disease compared with previous pivotal systemic therapy trials. Therefore, the study population may not fully represent the broader population of patients with advanced HCC treated with systemic therapy in routine practice.

In conclusion, although the primary endpoint of ORR did not achieve statistical significance, cisplatin HAIC followed by ATZ/BEV significantly improved PFS and was associated with a longer DoR without any additional toxicity compared to ATZ/BEV alone. These findings are hypothesis-generating and warrant confirmation in larger phase III trials before this strategy can be incorporated into routine clinical practice.

## Abbreviations

AE, adverse event; ATZ/BEV, atezolizumab plus bevacizumab; BCLC, Barcelona Clinical Liver Cancer classification; DoR, duration of response; ECOG PS, Eastern Cooperative Oncology Group performance status; HAIC, hepatic arterial infusion chemotherapy; HCC, hepatocellular carcinoma; ITT, intent-to-treat; ORR, objective response rate; OS, overall survival; PD, progressive disease; PFS, progression-free survival; PS, performance status.

## Authors’ contributions

Yasuto Takeuchi and Kazuhiro Nouso designed the study. Masahiko Sue, Nozomi Miyake, Takuya Adachi, Hideki Onishi, Miwa Kawanaka, Akinobu Takaki, Kazuya Kariyama, Tetsuya Yasunaka, Kenji Kuwaki, Junichiro Toshimori, Shota Iwado, Akio Moriya, Hiroaki Hagihara, Hiroyuki Takabatake, Masahiro Sakata, Takeshi Hatanaka, Shigeo Shimose, Takumi Kawaguchi and Yasuto Takeuchi collected and interpreted data. Yasuto Takeuchi had access to all of the data. Yasuto Takeuchi analyzed the data. Motoyuki Otsuka and Yasuto Takeuchi wrote the manuscript.

## Data availability

The data that support the findings of this study are not available due to institutional policy and patient confidentiality restrictions. Further enquiries can be directed to the corresponding author.

## Statement of ethics

This study protocol was reviewed and approved by Okayama University Certified Review Board, approval number CRB21-006.

## Financial support

This study was supported by 10.13039/100018046Nippon Kayaku Co., Ltd.

## Conflict of interest

The authors have no competing interests to declare. The funder played no role in study design, data collection, data analysis, data interpretation, or manuscript writing.

Please refer to the accompanying ICMJE disclosure forms for further details.
