# Supplementary material for: Atezolizumab–bevacizumab with or without cisplatin-based HAIC for unresectable HCC: A randomized phase II trial
Source: JHEP Rep. 2026 Aug 29;8(11):102002. doi: 10.1016/j.jhepr.2026.102002 (PMC13659464; doi:10.1016/j.jhepr.2026.102002)
Supplement: Supplementary file 1 [file mmc1.pdf]

Atezolizumab–bevacizumab with or without cisplatin-based HAIC for unresectable HCC: A randomized phase II trial

Yasuto Takeuchi, Masahiko Sue, Nozomi Miyake, Takuya Adachi, Hideki Onishi, Miwa Kawanaka, Akinobu Takaki, Kazuhiro Nouso, Kazuya Kariyama, Tetsuya Yasunaka, Kenji Kuwaki, Junichi Toshimori, Shota Iwado, Akio Moriya, Hiroaki Hagihara, Hiroyuki Takabatake, Masahiro Sakata, Takeshi Hatanaka, Shigeo Shimose, Takumi Kawaguchi, Motoyuki Otsuka

Table of contents

Fig. S1……………………………………………………………………………2

Fig. S2……………………………………………………………………………3

Fig. S3……………………………………………………………………………4

Table S1…………………………………………………………………………5

Supplementary figures

Supplementary Figure 1A

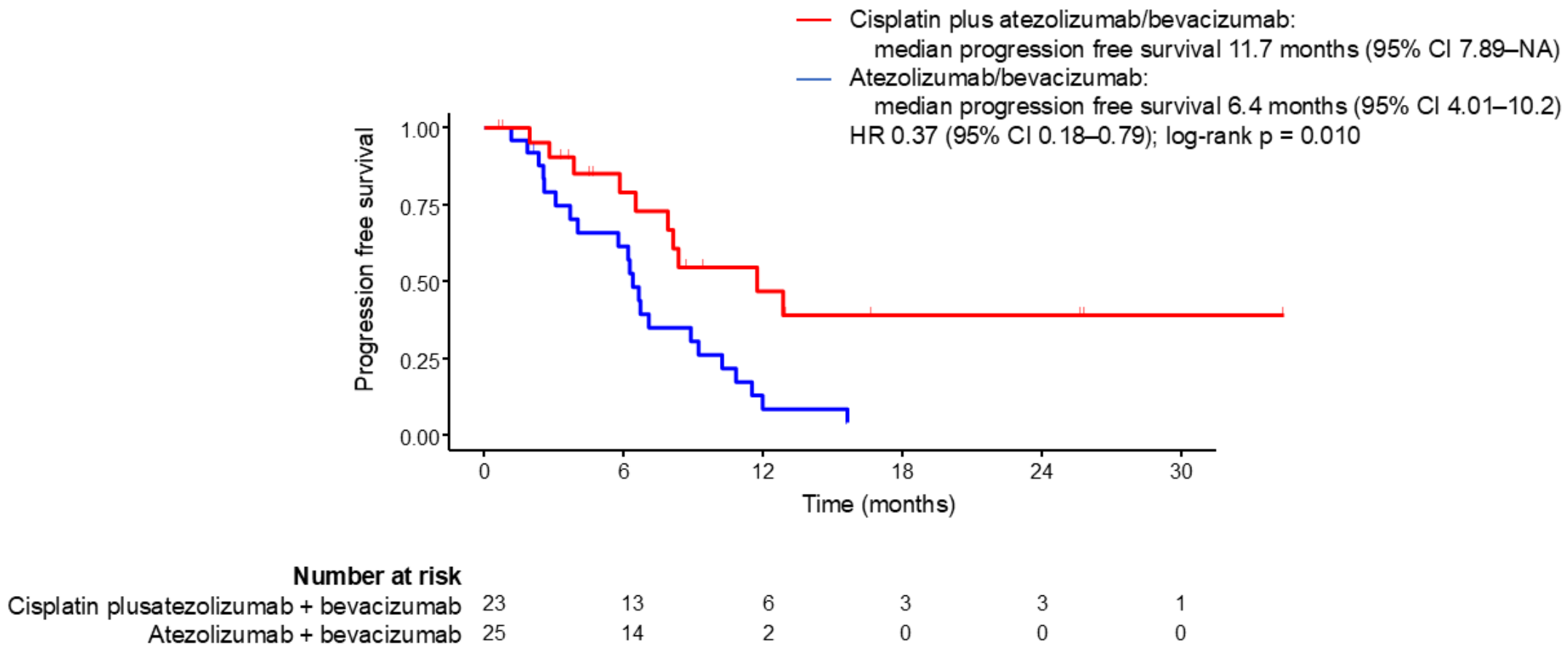

Supplementary Figure 1B

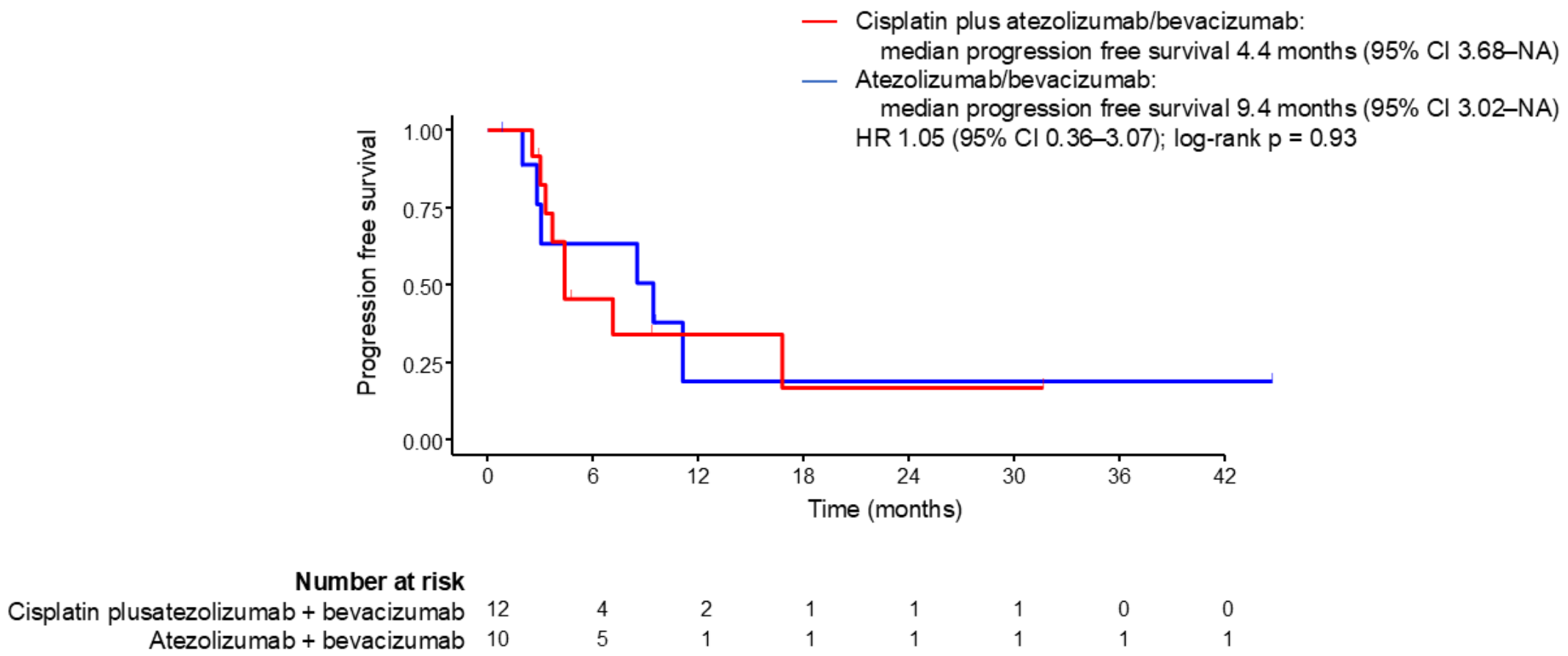

Fig. S1. Overall survival according to treatment group by macrovascular invasion. (A) Kaplan–Meier curves for overall survival comparing CDDP+ATZ/BEV and ATZ/BEV in patients with macrovascular invasion. Median overall survival was 11.7 months in the CDDP+ATZ/BEV group and 6.4 months in the ATZ/BEV group (HR, 0.37; 95% CI, 0.18–0.79; p = 0.010). (B) Kaplan–Meier curves for overall survival

comparing CDDP+ATZ/BEV and ATZ/BEV in patients without macrovascular invasion. Median overall survival was 4.4 months in the CDDP+ATZ/BEV group and 9.4 months in the ATZ/BEV group (HR, 1.05; 95% CI, 0.36–3.07; p = 0.93).

Supplementary Figure 2

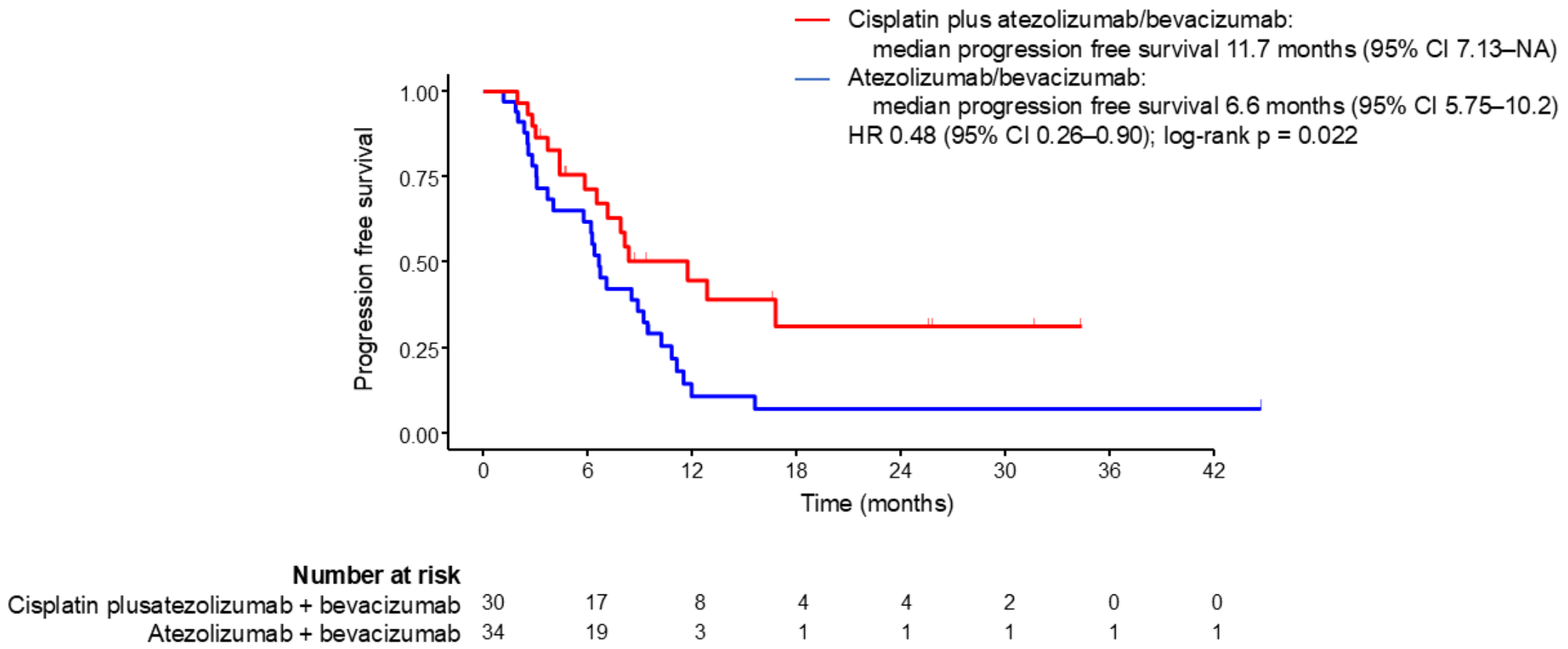

Fig. S2. Kaplan–Meier curves for progression-free survival in the safety/per-protocol population.

Patients who received the assigned treatment were included in this sensitivity analysis. Median progression-free survival was 7.5 months in the CDDP+ATZ/BEV group and 5.7 months in the ATZ/BEV group (hazard ratio, 0.48; 95% confidence interval, 0.26–0.90; p = 0.022).

Supplementary Figure 3

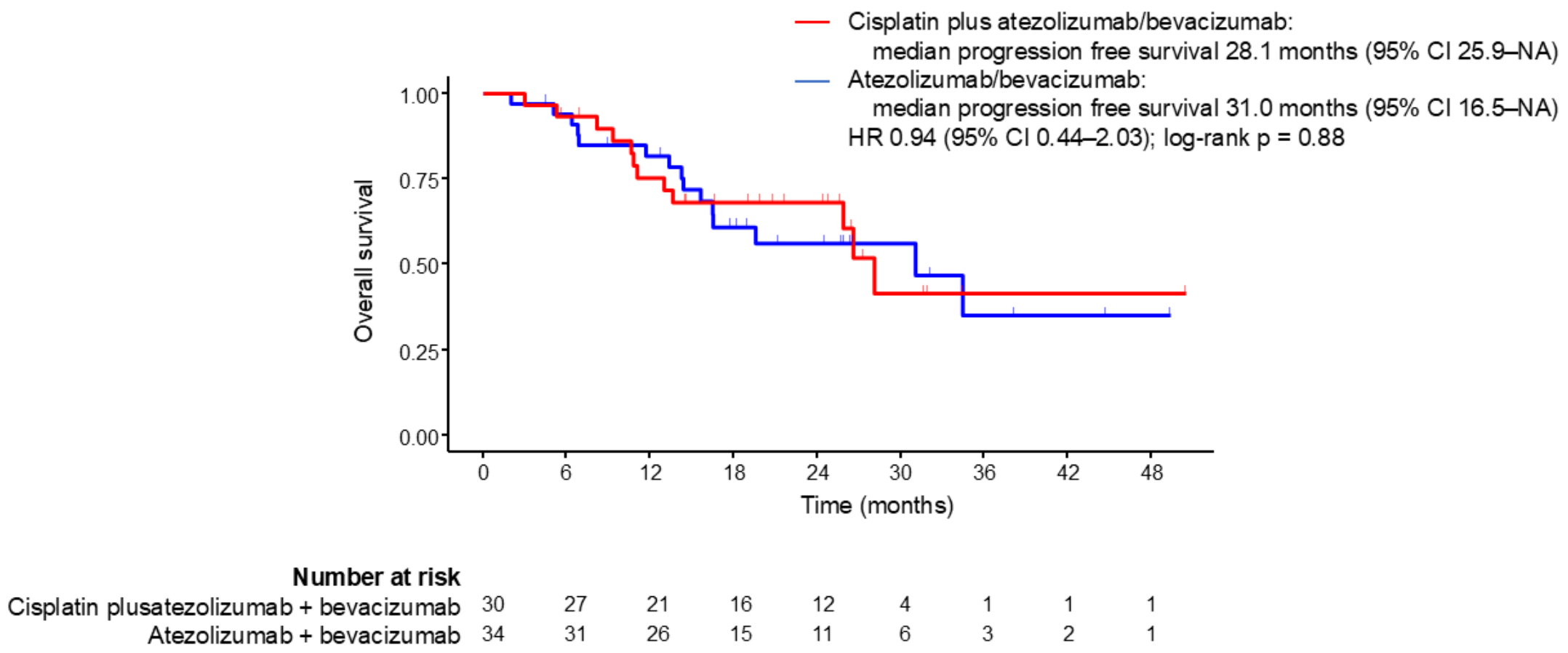

Fig. S3. Overall survival in the safety/per-protocol population.

Kaplan–Meier curves for overall survival comparing CDDP+ATZ/BEV and ATZ/BEV among patients who received the assigned treatment. Median overall survival was 28.1 months in the CDDP+ATZ/BEV group and 31.0 months in the ATZ/BEV group (hazard ratio, 0.94; 95% confidence interval, 0.44–2.03; p = 0.88).

Tables

Table S1: Tumor response outcomes in the per-protocol population

	Cisplatin plus atezolizumab + bevacizumab (n = 30)	Atezolizumab + bevacizumab (n = 34)	*P*-value
Objective response rate*, n (%)	16 (53.3)	10 (29.4)	0.059
Disease control rate*, n (%)	26 (86.7)	24 (70.6)	0.29
Best overall response			
Complete response, n (%)	2 (6.7)	1 (2.9)	
Partial response, n (%)	14 (46.7)	9 (26.5)	
Stable disease, n (%)	10 (33.3)	14 (41.2)	
Progressive disease, n (%)	3 (10.0)	8 (23.5)	
Not evaluable, n (%)	1 (3.3)	2 (5.9)	
Median time to response (IQR), months†	2.99 (2.00–4.89)	2.97 (2.14–5.55)	0.91

*Patients who had confirmed complete response or partial response per Response Evaluation Criteria in Solid Tumors (version 1.1). †Assessed in patients with a confirmed partial or complete response.
