# Supplementary material for: Atezolizumab–bevacizumab with or without cisplatin-based HAIC for unresectable HCC: A randomized phase II trial
Source: JHEP Rep. 2026 Aug 29;8(11):102002. doi: 10.1016/j.jhepr.2026.102002 (PMC13659464; doi:10.1016/j.jhepr.2026.102002)
Supplement: Supplementary file 3 [file mmc5.pdf]

Atezolizumab–bevacizumab with or without cisplatin-based HAIC for unresectable HCC: A randomized phase II trial

Authors

Yasuto Takeuchi, Masahiko Sue, Nozomi Miyake, …, Shigeo Shimose, Takumi Kawaguchi, Motoyuki Otsuka

Correspondence

pwqs6abz@s.okayama-u.ac.jp (Y. Takeuchi).

Graphical abstract

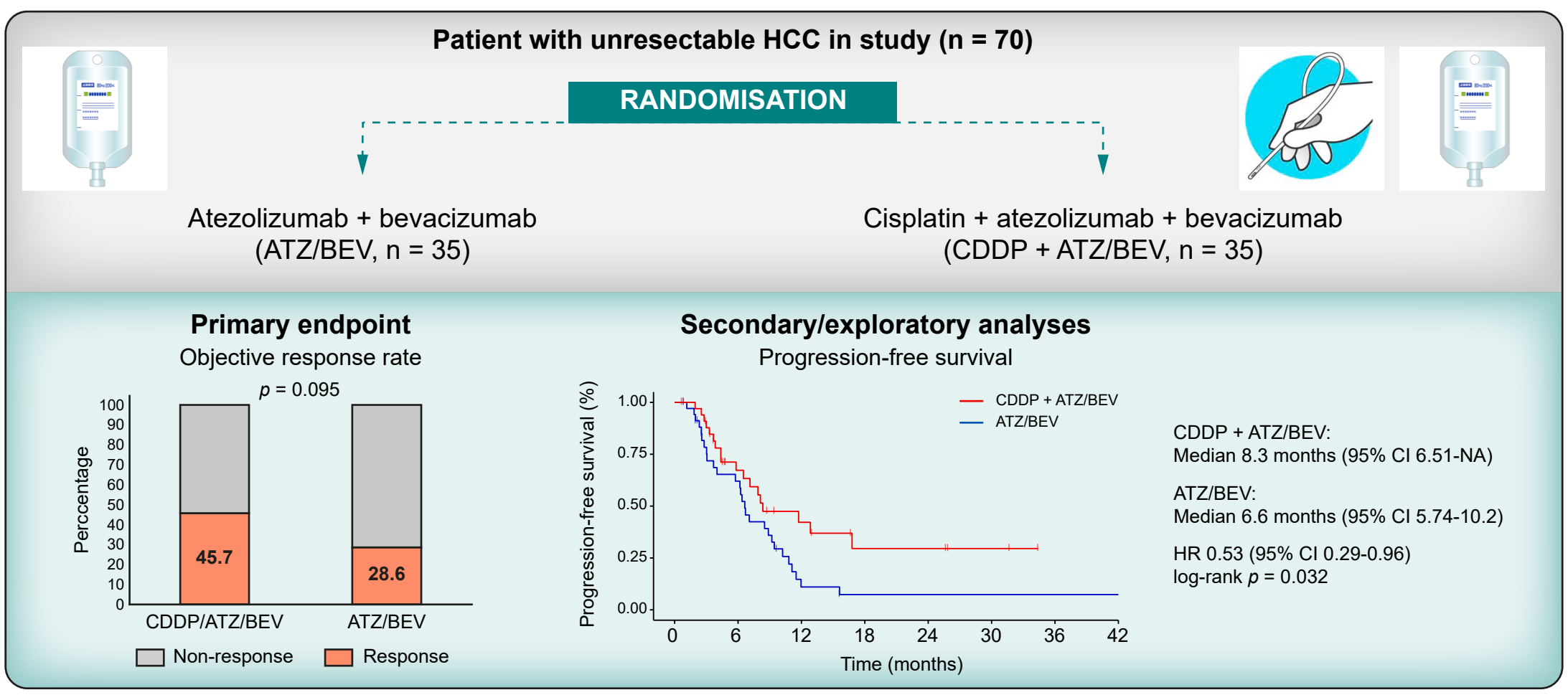

Highlights:

- This multicenter randomized phase II trial evaluated sequential cisplatin HAIC followed by atezolizumab–bevacizumab in unresectable HCC.
- The primary endpoint of objective response rate was numerically higher with cisplatin HAIC but did not reach statistical significance.
- Exploratory analyses demonstrated significantly longer progression-free survival without increased treatment-related toxicity.
- These findings support further evaluation of sequential cisplatin HAIC plus atezolizumab–bevacizumab in a phase III trial.

Impact and implications:

This multicenter randomized phase II trial provides evidence that sequential cisplatin hepatic arterial infusion chemotherapy followed by atezolizumab–bevacizumab may prolong disease control without increasing toxicity in patients with unresectable hepatocellular carcinoma. Although the primary endpoint was not met and the progression-free survival benefit was observed in exploratory analyses, these findings support further investigation of this strategy in phase III trials and provide a rationale for evaluating liver-directed combination approaches to optimize first-line immunotherapy.

https://doi.org/10.1016/j.jhepr.2026.102002
© 2026 The Author(s). Published by Elsevier B.V. on behalf of European Association for the Study of the Liver (EASL). This is an open access article under the CC BY license (http://creativecommons.org/licenses/by/4.0/). JHEP Reports, 2026, 8, 1–9

JHEP|Reports

Atezolizumab–bevacizumab with or without cisplatin-based HAIC for unresectable HCC: A randomized phase II trial

Yasuto Takeuchi[1,*], Masahiko Sue[1], Nozomi Miyake[1], Takuya Adachi[1], Hideki Onishi[1], Miwa Kawanaka[1], Akinobu Takaki[1], Kazuhiro Nouso[2], Kazuya Kariyama[2], Tetsuya Yasunaka[3], Kenji Kuwaki[4], Junichi Toshimori[5], Shota Iwado[6], Akio Moriya[7], Hiroaki Hagihara[8], Hiroyuki Takabatake[9], Masahiro Sakata[10], Takeshi Hatanaka[11], Shigeo Shimose[12], Takumi Kawaguchi[12], Motoyuki Otsuka[1]

JHEP Reports **2026**. vol. 8 | 1–9

Check for updates

Background & Aims: Atezolizumab plus bevacizumab (ATZ/BEV) is the global standard first-line treatment for unresectable hepatocellular carcinoma (HCC). However, the objective response rate (ORR) is approximately 30%. Hepatic arterial infusion chemotherapy (HAIC) with cisplatin may enhance its efficacy through locoregional cytoreduction and immunological modulation. We conducted a randomized phase II trial to evaluate whether sequential cisplatin HAIC plus ATZ/BEV (CDDP+ATZ/BEV) improves clinical outcomes compared to ATZ/BEV alone.

Methods: In this multicenter, open-label trial, patients with unresectable HCC were randomized 1:1 to receive cisplatin HAIC within 8 weeks before initiation of ATZ/BEV or ATZ/BEV alone. The primary endpoint was ORR.

Results: Between December 2021 and December 2024, 70 patients were enrolled in this study (CDDP+ATZ/BEV, n = 35; ATZ/BEV, n = 35). The primary endpoint, ORR, was numerically higher in the CDDP+ATZ/BEV group than in the ATZ/BEV group but did not reach statistical significance (45.7% *vs.* 28.6%; p = 0.095). Median progression-free survival (PFS) was longer in the CDDP+ATZ/BEV group (8.3 *vs.* 6.6 months; HR 0.53; p = 0.036), and median duration of response was not reached *vs.* 4.6 months (HR 0.4; p = 0.11). The median overall survival (OS) was 28.1 *vs.* 31.0 months (HR 0.95; p = 0.88). Grade 3–4 treatment-related adverse events occurred in 20.0% and 22.9% of patients, respectively.

Conclusions: The primary endpoint of ORR was not met. However, exploratory analyses demonstrated longer PFS without additional safety concerns. Further evaluation in phase III trials is warranted.

Clinical trial number: Japan Registry of Clinical Trials (jRCTs061210065).

© 2026 The Author(s). Published by Elsevier B.V. on behalf of European Association for the Study of the Liver (EASL). This is an open access article under the CC BY license (http://creativecommons.org/licenses/by/4.0/).

Introduction

Hepatocellular carcinoma (HCC) remains a leading cause of cancer-related mortality worldwide, especially in cases of unresectable disease where the prognosis is poor despite advances in systemic therapies. The combination of the immune checkpoint inhibitor atezolizumab and the VEGF-targeting agent bevacizumab (ATZ/BEV) has become the global standard first-line treatment, following the compelling survival benefits demonstrated in the IMbrave150 trial.[1] Nevertheless, the objective response rate (ORR) with ATZ/BEV remains at approximately 30%, indicating that most patients fail to achieve significant tumor shrinkage.

Cisplatin-based hepatic arterial infusion chemotherapy (HAIC) delivers high intrahepatic drug concentrations with meaningful tumor responses and limited systemic toxicity.[2–4] Beyond local cytoreduction, liver-directed therapies, such as HAIC or transarterial chemoembolization (TACE), may also modulate systemic antitumor immunity. Preclinical and translational studies have suggested that locoregional tumor destruction can release tumor antigens, induce immunogenic cell death, and enhance T-cell priming.[5] Furthermore, combining these liver-directed therapies with VEGF inhibitors may normalize the tumor vasculature, reduce immunosuppressive myeloid cells, and facilitate immune infiltration, creating a more permissive tumor microenvironment for immune checkpoint blockade.[6,7] Collectively, these findings provide a strong biological rationale for combining HAIC with ATZ/BEV, aiming to augment both local control and the systemic immune response.

Building on this concept, we designed a multicenter, randomized, phase II trial to test whether sequential cisplatin HAIC followed by ATZ/BEV could improve outcomes compared to that with ATZ/BEV alone in patients with unresectable HCC. We hypothesized that the addition of HAIC

* Corresponding author. Address: Department of Gastroenterology, Okayama University Hospital 2-5-1, Shikata-cho, Kita-ku, Okayama City, Okayama 700-8558, Japan; Tel.: +81 86 235 7218, fax: +81 86 225 5991.
E-mail address: pwqs6abz@s.okayama-u.ac.jp (Y. Takeuchi).
https://doi.org/10.1016/j.jhepr.2026.102002

would increase the tumor response through both direct cytoreduction and immunologic modulation, thereby prolonging disease control without compromising safety.

Patients and methods

Study design and participants

Eligible patients were adults (aged ≥18 years) with a confirmed diagnosis of unresectable HCC. Unresectability was determined by a multidisciplinary tumor board based on tumor extent, liver function, and patient performance status. All patients were required to have at least one measurable intrahepatic lesion according to RECIST version 1.1[8] because it has been widely adopted in contemporary clinical trials of immune checkpoint inhibitor-based systemic therapies for HCC and facilitates comparison with established efficacy data from pivotal studies.

Additional inclusion criteria were an Eastern Cooperative Oncology Group (ECOG) performance status of 0 or 1 and preserved liver function defined as a Child–Pugh score of 5–7. Patients were required to be naïve to cisplatin-based HAIC.

Key exclusion criteria included a history of two or more prior TACE procedures, absence of intrahepatic target lesions, and known hypersensitivity to cisplatin or to either component of atezolizumab plus bevacizumab therapy. Patients were also excluded if they had contraindications to immune checkpoint inhibitors or molecular targeted agents, including but not limited to active autoimmune disease requiring systemic immunosuppression. Additional exclusion criteria were the presence of severe cardiovascular disease and impaired renal function, defined as an estimated glomerular filtration rate (eGFR) <30 ml/min/1.73 m^2 (Fig. 1).

The study was conducted in accordance with the principles of the Declaration of Helsinki and the International Council for Harmonization Good Clinical Practice guidelines. The study protocol was reviewed and approved by the Okayama University Certified Review Board (approval number: CRB21-006). Written informed consent was obtained from all participants prior to enrollment.

Randomization and procedures

Patients were randomly assigned (1:1) using a REDCap (Research Electronic Data Capture) system,[9,10] stratified by previous TACE treatment (yes *vs.* no) and the presence of macrovascular invasion. The trial was an open label.

In the experimental arm, patients received a single infusion of cisplatin (65 mg/m^2) as HAIC within 8 weeks before initiation of atezolizumab plus bevacizumab. No additional HAIC cycles were planned or permitted after initiation of systemic therapy. The control arm was administered ATZ/BEV at identical doses and schedules. HAIC administration was mandatory prior to ATZ/BEV administration in the experimental arm. For cisplatin hepatic artery infusion, the site was the proper hepatic artery when tumors were present in both lobes of the liver, and the right or left hepatic artery when tumors were present in only one lobe. If patients experienced HAIC-related adverse events, subsequent ATZ/BEV treatment was permitted, provided that the predefined discontinuation criteria were not met.

Treatment was continued until disease progression, unacceptable toxicity, or withdrawal of consent. Treatment beyond progression was permitted when the patient was receiving clinical benefit, at the investigator's discretion. No dose escalation or reduction of ATZ or BEV was allowed. However, treatment delays with ATZ, BEV, or both were permitted in the event of adverse events (AEs). Intervention with local therapy during treatment was not permitted; however, after progression, intervention with local therapy was permitted at the discretion of the attending physician.

Tumor assessments were performed at baseline, at weeks 9–12, and every 12 weeks thereafter, using contrast-enhanced computed tomography or magnetic resonance imaging and covering all known or suspected disease sites. Baseline tumor evaluation was performed within 28 days before randomization. Subsequent assessments were scheduled 9–12 weeks (±1 week) after the initiation of ATZ/BEV and every 12 weeks (±1 week) thereafter, until progressive disease or treatment discontinuation, whichever occurred later. Changes in tumor measurements and treatment responses were assessed by the

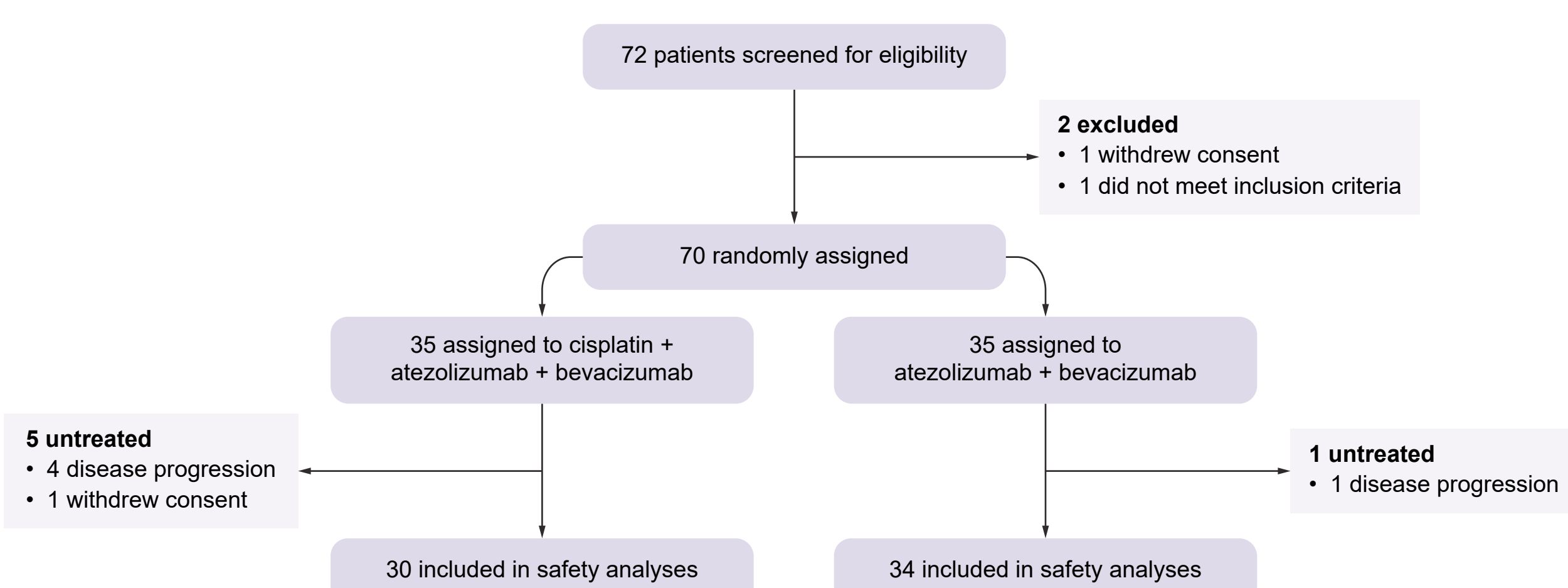

Fig. 1. CONSORT flow diagram of patient enrollment, randomization, treatment allocation, and analysis populations. A total of 70 patients were randomized to receive either cisplatin-based hepatic arterial infusion chemotherapy followed by atezolizumab + bevacizumab (n = 35) or atezolizumab plus bevacizumab alone (n = 35). All randomized patients were included in the intention-to-treat efficacy analyses. Patients who received the assigned treatment were included in the safety and per-protocol analyses.

investigators according to RECIST version 1.1, which guides decisions regarding treatment continuation.

Outcomes

The primary endpoint was the objective response rate (ORR), defined as the proportion of patients achieving the best overall response of complete response or partial response according to RECIST version 1.1.

Secondary endpoints included progression-free survival (PFS), disease control rate, duration of response (DoR), overall survival (OS), and safety. PFS and OS were calculated from the time of randomization. PFS was defined as the time from randomization to disease progression or death from any cause, whichever occurred first, and OS as the time from randomization to death from any cause.

For the safety assessment, all AEs were monitored throughout the treatment period and for 48 weeks after treatment initiation, as specified in the protocol, even if the treatment was discontinued earlier. AEs were defined and graded according to the National Cancer Institute CTCAE version 5.0.[11]

The effect of baseline demographic and clinical characteristics on PFS was evaluated in pre-specified subgroup analyses of all randomly assigned patients, excluding those with an undetermined best overall response. The subgroups included standard demographic variables and stratification factors: age, sex, Child–Pugh score, etiology, presence of macrovascular invasion, extrahepatic spread, baseline alpha-fetoprotein concentration, and baseline Barcelona Clinic Liver Cancer (BCLC) stage.[12]

Data collection

Study data were collected and managed using REDCap electronic data capture tools hosted at Okayama University Hospital, which is a secure web-based software platform designed to facilitate research data management, offering 1) an intuitive interface for validated data entry, 2) audit trails for tracking data manipulation and export, 3) automated export procedures for seamless transfer to common statistical packages, and 4) features for integration and interoperability with external data sources. The data cut-off date for the present analysis was March 31, 2026.

Sample size and statistical analysis

During protocol development and prior to patient enrollment, the sample size calculation was revised based on updated efficacy results from the IMbrave150 trial. The original protocol assumed efficacy estimates derived from the initial IMbrave150 report; however, subsequently published updated data demonstrated improved treatment outcomes with atezolizumab plus bevacizumab. As a result, the expected response rate in the control arm was revised upward, and the sample size calculation was recalculated accordingly, resulting in a reduction in the required sample size. This amendment was completed and approved before study initiation.

All eligible patients registered in this study were included in the analysis. Patient demographics, baseline clinical characteristics, and tumor status were summarized as aggregated data. Continuous variables were presented as medians with interquartile ranges (25th–75th percentiles). The primary efficacy analyses were conducted in the intention-to-treat (ITT) population, defined as all randomized patients according to their assigned treatment group, regardless of treatment initiation or protocol adherence. Safety analyses were performed in all patients who received at least one dose of study treatment (safety population). Additional per-protocol analyses were performed as sensitivity analyses in patients who received the assigned treatment.

Differences in objective response rate between the treatment groups were assessed using a two-sided Fisher's exact test. PFS, DoR, and OS were estimated using the Kaplan–Meier method, and hazard ratios (HRs) with 95% CIs were calculated using Cox proportional hazards models. Safety was assessed in all randomly assigned patients who enrolled in the study.

All statistical analyses were performed using the R software version 4.4.0. All tests were two-sided, and p values <0.05 were considered statistically significant.

Results

Patient characteristics

Between December 21, 2021, and December 31, 2024, a total of 72 patients with unresectable HCC provided written informed consent and were screened for eligibility. Of these, one patient withdrew consent prior to enrollment, and one patient was excluded because the eligibility criteria were not met. The remaining 70 eligible patients were randomized in a 1:1 ratio to receive cisplatin-based HAIC plus atezolizumab and bevacizumab (CDDP+ATZ/BEV group, n = 35) or atezolizumab plus bevacizumab alone (ATZ/BEV group, n = 35). In the CDDP+ATZ/BEV group, one patient withdrew consent and four patients experienced rapid disease progression before initiation of the assigned treatment, resulting in 30 patients who actually received the study treatment. In the ATZ/BEV group, one patient was unable to initiate treatment because of disease progression prior to treatment start, leaving 34 patients who received the assigned therapy. Consequently, 70 patients were included in the ITT efficacy analyses according to their randomized treatment assignment. Among them, 30 patients in the CDDP+ATZ/BEV group and 34 patients in the ATZ/BEV group received the assigned treatment and were included in the safety and per-protocol analyses. There were no differences in baseline demographics and disease characteristics between the two groups (Table 1).

The median age was 74 years in the CDDP+ATZ/BEV group and 75 years in the ATZ/BEV group. Most patients were male (85.7% in the CDDP+ATZ/BEV group *vs.* 80.0% in the ATZ/BEV group), and viral etiologies accounted for 37.1% and 28.6% of cases, respectively. The majority of the participants had an ECOG performance status of 0. Most patients had a Child–Pugh score of 5 (60.0% *vs.* 62.9%). The proportions of patients with BCLC stage C disease were 54.2% and 40.0%, respectively. The prevalence of macrovascular invasion and extrahepatic spread was comparable between the two groups (34.3% *vs.* 28.6% and 31.4% *vs.* 20.0%, respectively).

Efficacy outcomes

The ORR was 45.7% in the CDDP+ATZ/BEV group and 28.6% in the ATZ/BEV group (p = 0.095). Complete responses occurred in 5.7% and 2.9% of patients, respectively, and the disease control rates were 74.3% and 68.6%, respectively (Table 2).

Table 1. Baseline patient demographics and disease characteristics.

	Cisplatin + atezolizumab + bevacizumab (n = 35)	Atezolizumab + bevacizumab (n = 35)	*p* value
**Patient demographics**			
Age, years	74 (73–79)	75 (63–77)	0.19
Sex			0.52
Male, n (%)	30 (85.7)	28 (80.0)	
Female, n (%)	5 (14.3)	7 (20.0)	
Etiology			0.45
Viral, n (%)	13 (37.1)	10 (28.6)	
Non-viral, n (%)	22 (62.9)	25 (71.4)	
ECOG PS			0.23
0, n (%)	26 (74.3)	30 (85.7)	
1, n (%)	9 (25.7)	5 (14.3)	
**Liver function**			
Child-Pugh score			0.81
5, n (%)	21 (60.0)	22 (62.9)	
6, n (%)	14 (40.0)	13 (37.1)	
ALBI score	-2.30 (-2.45– -2.09)	-2.39 (-2.67– -2.17)	0.40
Creatinine	0.78 (0.68–0.96)	0.75 (0.65–0.90)	0.20
**Tumor characteristics**			
BCLC stage			0.18
0/A	1 (2.9)	5 (14.3)	
B	15 (42.9)	16 (45.7)	
C	19 (54.2)	14 (40.0)	
Maximum intrahepatic tumor diameter (mm)	51 (25–100)	30 (15–100)	0.26
Number of nodules			0.33
1, n (%)	7 (20.0)	7 (20.0)	
2-3, n (%)	7 (20.0)	12 (34.3)	
≥ 4, n (%)	21 (60.0)	16 (45.7)	
Macrovascular invasion, n (%)	12 (34.3)	10 (28.6)	0.61
Extrahepatic spread, n (%)	11 (31.4)	7 (20.0)	0.27
AFP (ng/ml)	20 (5.9–508.3)	138 (10.1–978)	0.12
AFP-L3 (%)	4.7 (0.5–23.5)	23.3 (0.9–51.6)	0.52
DCP (mAU/ml)	1,377 (73–17,486)	517 (64–4,238)	0.77
**Previous treatment**			
Previous TACE, n (%)	11 (31.4)	12 (34.3)	0.80
Previous systemic therapy, n (%)	2 (5.7)	0 (0.0)	0.15
**Treatment characteristics**			
Period until ATZ/BEV	5 (4–6)	–	–

Data are presented as medians (interquartile ranges) or n (%) unless otherwise indicated. AFP, alpha-fetoprotein; AFP-L3, Lens culinaris agglutinin-reactive alpha-fetoprotein isoform; ALBI, albumin–bilirubin; BCLC, Barcelona Clinic Liver Cancer; DCP, des-gamma-carboxy prothrombin; PS, performance status. *p* values were calculated using the Mann–Whitney U test for continuous variables and Pearson's chi-square test for categorical variables.

For the key secondary endpoint, PFS, CDDP+ATZ/BEV demonstrated a statistically significant benefit over ATZ/BEV (median 8.3 *vs.* 6.6 months; HR 0.53, 95% CI 0.29–0.96; p = 0.036; Fig. 2A). Subgroup analyses are shown in Fig. 2B. HRs and 95% CIs for each subgroup are presented in the forest plot. Statistically significant differences were observed in the following subgroups: age ≥65 years, male sex, viral hepatitis, presence of macrovascular invasion, presence of extrahepatic spread, and AFP ≥400 ng/ml.

In the exploratory subgroup analysis, the treatment effect on PFS according to macrovascular invasion status was assessed using an interaction test. Kaplan–Meier curves stratified by the presence or absence of macrovascular invasion are shown in Fig. S1. Although the estimated HR favored CDDP+ATZ/BEV more strongly in patients with macrovascular invasion, the interaction test did not demonstrate a statistically significant interaction between treatment effect and macrovascular invasion status (p for interaction = 0.084).

The median DoR was not reached (95% CI 7.89–NA) in the CDDP+ATZ/BEV group *vs.* 4.6 months (95% CI 3.22–NA) in the ATZ/BEV group. The HR for DoR was 0.40 (95% CI 0.13–1.22; p = 0.11) (Fig. 3). Following study treatment, two patients in the CDDP+ATZ/BEV group and one patient in the ATZ/BEV group underwent curative-intent treatment (surgical resection or local ablation).

At a median follow-up of 16.7 months, the median OS was 28.1 months (95% CI 25.9–NA) in the CDDP+ATZ/BEV group *vs.* 31.0 months (95% CI 16.5–NA) in the ATZ/BEV group (HR 0.95, 95% CI 0.46–1.95; Fig. 4).

Safety

Treatment-related AEs (TRAEs) were observed in 48.6% and 51.5% of patients in the CDDP+ATZ/BEV group and ATZ/BEV groups, respectively (Table 3). Grade 3–4 TRAEs were reported in 20.0% and 22.9% of patients, respectively. The most common AEs were hepatic dysfunction, proteinuria, and variceal bleeding. To date, no grade 5 TRAEs have been reported. The rate of treatment discontinuation due to TRAEs was comparable between groups (17.2% *vs.* 20.1%).

A

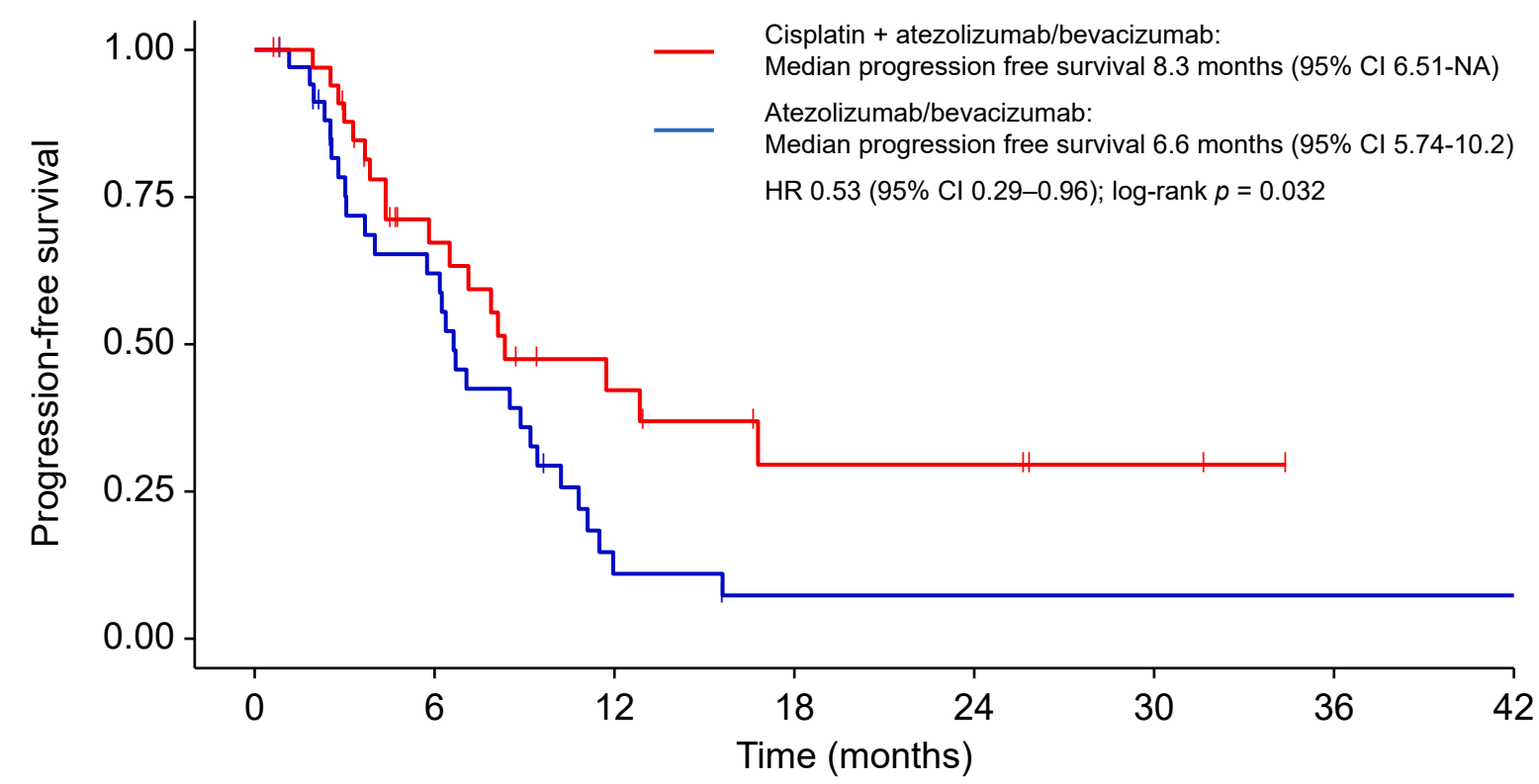

N° at risk								
Cisplatin + atezolizumab + bevacizumab	35	17	8	4	4	2	0	0
Atezolizumab + bevacizumab	35	19	3	1	1	1	1	1

B

	Patients	Median progression-free survival (95% CI), months: Cisplatin + atezolizumab + bevacizumab	Atezolizumab + bevacizumab	HR (95% CI)	*p* value
**Age, years**					
<65	12	6.5 (3.8-NA)	9.7 (8.5-NA)	1.39 (0.33-5.77)	0.65
≥65	58	8.3 (7.1-NA)	6.2 (4.0-9.4)	0.40 (0.20-0.78)	<0.01
**Sex**					
Male	58	8.3 (6.5-NA)	6.4 (4.0-10.2)	0.42 (0.22-0.82)	0.011
Female	12	7.1 (3.7-NA)	7.6 (2.5-NA)	0.78 (0.14-4.37)	0.78
**Etiology**					
Viral	23	11.7 (4.4-NA)	2.9 (2.0-NA)	0.31 (0.10-0.90)	0.032
Non-viral	47	8.1 (3.7-NA)	7.1 (6.2-12.0)	0.63 (0.30-1.33)	0.23
**Child-pugh score**					
5	43	7.9 (6.5-NA)	7.1 (3.1-11.5)	0.55 (0.25-1.19)	0.13
6	27	11.7 (3.7-NA)	6.6 (6.2-NA)	0.52 (0.20-1.36)	0.18
**Macrovascular invasion**					
Yes	22	11.7 (7.9-NA)	6.4 (4.0-10.2)	0.37 (0.18-0.79)	0.010
No	48	4.4 (3.7-NA)	9.4 (3.0-NA)	1.05 (0.36-3.07)	0.93
**Extrahepatic spread**					
Yes	15	12.8 (8.1-NA)	5.2 (2.8-NA)	0.44 (0.10-1.85)	0.26
No	55	8.3 (5.8-NA)	6.7 (5.7-10.8)	0.59 (0.30-1.14)	0.12
**BCLC**					
0 or A	6	NR	7.1 (2.3-NA)	0.00 (0.00-Inf)	0.99
B	31	7.9 (5.8-NA)	6.4 (6.4-12.0)	0.56 (0.22-1.40)	0.22
C	23	8.1 (4.4-NA)	8.5 (3.0-NA)	0.58 (0.25-1.36)	0.21
**Alpha-fetoprotein, ng/ml**					
<400	51	11.7 (7.1-NA)	8.9 (6.6-11.5)	0.60 (0.29-1.23)	0.16
≥400	19	8.1 (3.7-NA)	3.0 (2.6-NA)	0.25 (0.08-0.86)	0.028

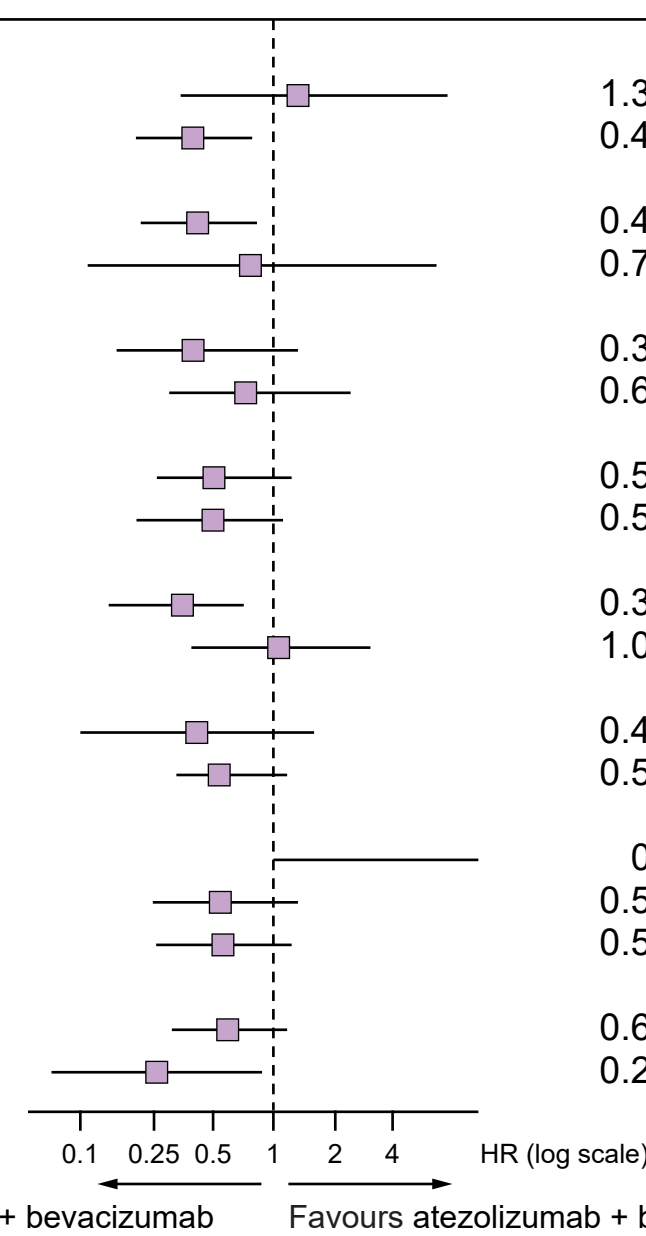

Fig. 2. Progression-free survival and forest plot. (A) Kaplan–Meier estimates of progression-free survival for patients treated with cisplatin-based HAIC plus atezolizumab plus bevacizumab (n = 35) and those treated with atezolizumab plus bevacizumab alone (n = 35). Median progression-free survival was 8.3 months (95% CI 6.50–NA) in the cisplatin-based HAIC plus atezolizumab plus bevacizumab group *vs.* 6.6 months (95% CI 5.75–10.2) in the atezolizumab plus bevacizumab alone group (HR 0.53; 95% CI 0.29–0.96; *p* = 0.036). (B) Forest plot of prespecified subgroup analyses. Treatment benefit with cisplatin-based HAIC plus atezolizumab plus bevacizumab over atezolizumab plus bevacizumab was generally consistent across age, sex, etiology, presence of macrovascular invasion, baseline alpha-fetoprotein level, and Barcelona Clinic Liver Cancer stage. HRs are shown with 95% CIs. Numbers at risk and censoring marks are shown below the Kaplan–Meier plot. HAIC, hepatic arterial infusion chemotherapy; HR, hazard ratio; PFS, progression-free survival. PFS was estimated using the Kaplan–Meier method. Hazard ratios and 95% confidence intervals were estimated using Cox proportional hazards models, and between-group differences were assessed using the log-rank test. *p* values for interaction were obtained from Cox proportional hazards models including treatment-by-subgroup interaction terms.

Sensitivity analyses

Sensitivity analyses performed in the per-protocol population demonstrated findings generally consistent with those observed in the ITT population. The objective response rate remained higher in the CDDP+ATZ/BEV group, and treatment effects on PFS and OS were similar to those observed in the primary analyses. Detailed results are provided in Table S1 and Figs. S2 and S3.

Discussion

In this multicenter, randomized, phase II trial, the addition of cisplatin-based HAIC prior to ATZ/BEV was compared with ATZ/BEV alone in patients with unresectable HCC. This randomized phase II trial did not meet its primary endpoint of ORR in the ITT population. However, exploratory analyses demonstrated longer PFS and DoR in the CDDP+ATZ/BEV arm. These findings should be interpreted cautiously given the

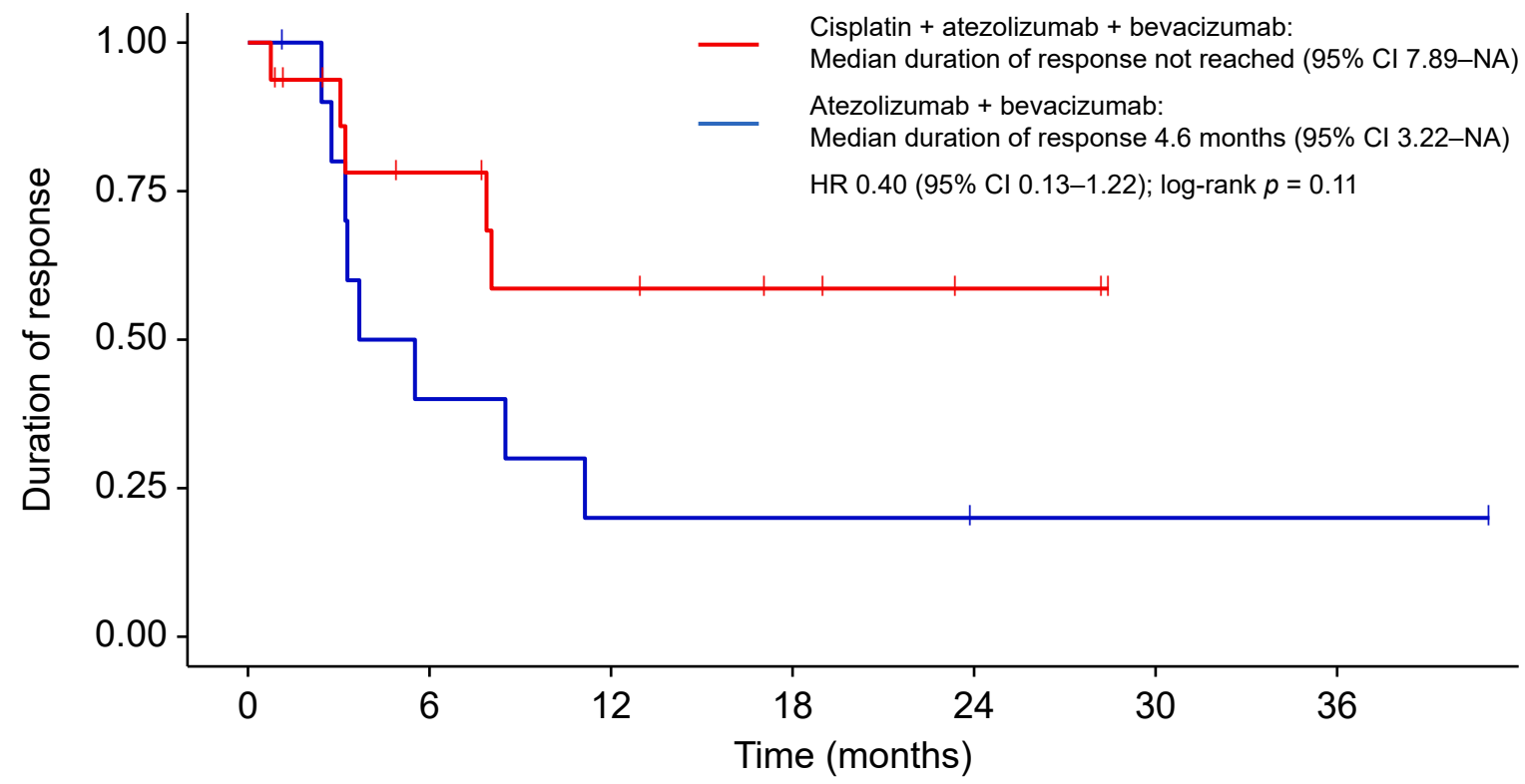

N° at risk							
Cisplatin + atezolizumab + bevacizumab	16	9	6	4	2	0	0
Atezolizumab + bevacizumab	11	4	2	2	1	1	1

Fig. 3. Duration of response among responders. Kaplan–Meier estimates of duration of response in patients who achieved complete or partial response. The median duration of response was not reached (95% CI 7.89–NA) in the cisplatin-based HAIC plus atezolizumab plus bevacizumab group *vs*. 4.6 months (95% CI 3.22–NA) in the atezolizumab + bevacizumab alone group (HR, 0.40; 95% CI 0.13–1.22; *p* = 0.11). The numbers at risk and the censoring marks are shown in the plot below. HAIC, hepatic arterial infusion chemotherapy; HR, hazard ratio. DoR was estimated using the Kaplan–Meier method. Hazard ratios and 95% confidence intervals were estimated using Cox proportional hazards models, and between-group differences were assessed using the log-rank test.

Table 2. Tumor response outcomes.

	Cisplatin + atezolizumab + bevacizumab (n = 35)	Atezolizumab + bevacizumab (n = 35)	*p* value
Objective response rate[a], n (%)	16 (45.7)	10 (28.6)	0.095
Disease control rate[a], n (%)	26 (74.3)	24 (68.6)	0.19
Best overall response			
Complete response, n (%)	2 (5.7)	1 (2.9)	
Partial response, n (%)	14 (40.0)	9 (25.7)	
Stable disease, n (%)	10 (28.6)	14 (40.0)	
Progressive disease, n (%)	3 (8.6)	8 (22.9)	
Not evaluable, n (%)	6 (17.1)	3 (8.5)	
Median time to response (IQR), months[b]	2.99 (2.00–4.89)	2.97 (2.14–5.55)	0.91

Between-group differences in ORR and DCR were assessed using Cochran–Mantel–Haenszel test, and time to response was compared using the Mann–Whitney *U* test.
[a]Patients who had confirmed complete response or partial response per RECIST (version 1.1).
[b]Assessed in patients with a confirmed partial or complete response.

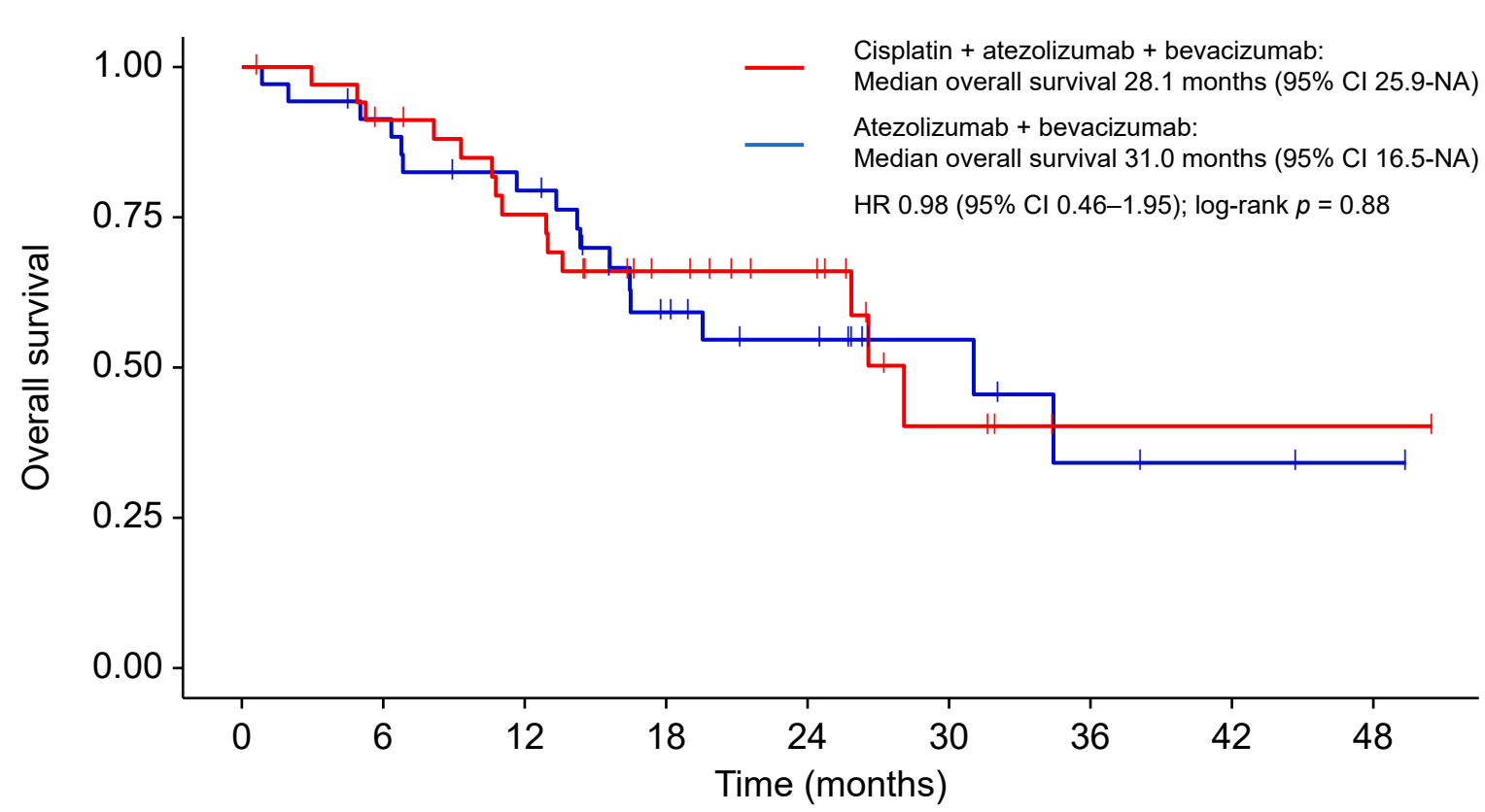

N° at risk									
Cisplatin + atezolizumab + bevacizumab	35	30	24	16	12	4	1	1	1
Atezolizumab + bevacizumab	35	31	26	15	11	6	3	2	1

Fig. 4. Overall survival in the intention-to-treat population. Kaplan–Meier estimates of overall survival in both treatment groups. At a median follow-up of 16.7 months, the median overall survival was 28.1 months (95% CI 25.9–NA) in the cisplatin-based HAIC plus atezolizumab plus bevacizumab group, compared with 31.0 months (95% CI 16.5–NA) in the atezolizumab + bevacizumab alone group (HR, 0.95; 95% CI 0.46–1.95). The numbers at risk and the censoring marks are shown in the plot below. HAIC, hepatic arterial infusion chemotherapy; HR, hazard ratio. OS was estimated using the Kaplan–Meier method. Hazard ratios and 95% confidence intervals were estimated using Cox proportional hazards models, and between-group differences were assessed using the log-rank test.

Table 3. Treatment-related adverse events in all treated patients.[a]

	Cisplatin + atezolizumab + bevacizumab (n = 35)		Atezolizumab + bevacizumab (n = 35)	
	Grade 1-2	Grade 3-4[b]	Grade 1-2	Grade 3-4[b]
Any treatment-related adverse event, n (%)	10 (28.6)	7 (20.0)	10 (28.6)	8 (22.9)
Treatment-related adverse event leading to discontinuation[c], n (%)	1 (2.9)	5 (14.3)	2 (5.8)	5 (14.3)
Treatment-related adverse events				
Fever	1 (2.9)	0	5 (14.3)	0
Hepatic failure	0	1 (2.9)	3 (8.6)	1 (2.9)
Proteinuria	1 (2.9)	1 (2.9)	1 (2.9)	1 (2.9)
Edema	1 (2.9)	0	3 (8.6)	0
Variceal bleeding	1 (2.9)	1 (2.9)	0	2 (5.8)
AST increased	1 (2.9)	1 (2.9)	0	1 (2.9)
ALT increased	1 (2.9)	1 (2.9)	0	1 (2.9)
Hepatic encephalopathy	0	0	1 (2.9)	2 (5.8)
Hypertension	0	0	1 (2.9)	1 (2.9)
Joint pain	1 (2.9)	0	1 (2.9)	0
Neutropenia	0	1 (2.9)	0	1 (2.9)
Pneumonitis	1 (2.9)	1 (2.9)	0	0
Tumor hemorrhage	1 (2.9)	0	0	1 (2.9)
Colitis	0	1 (2.9)	0	0
Cytokine release syndrome	0	0	0	1 (2.9)
Decreased appetite	1 (2.9)	0	0	0
Diarrhea	1 (2.9)	0	0	0
Fatigue	0	0	1 (2.9)	0
Hyperglycemia	0	1 (2.9)	0	0
Hyperthyroidism	1 (2.9)	0	0	0
Hypoadrenocorticism	0	0	0	1 (2.9)
Hypothyroidism	0	0	1 (2.9)	0
Nausea	1 (2.9)	0	0	0
Platelet count decreased	0	1 (2.9)	0	0

Data are n (%). Adverse events were graded per CTCAE v5.0.
ALT, alanine aminotransferase; AST, aspartate aminotransferase.
[a]Includes patients who received at least one dose of the study treatment.
[b]No grade 5 treatment-related adverse events occurred in either treatment group.
[c]This category refers to adverse events leading to discontinuation of any drug in the regimen.

negative primary endpoint and limited sample size. However, these findings suggest that the sequential administration of cisplatin HAIC followed by ATZ/BEV may provide clinically meaningful therapeutic advantages. Importantly, the findings of the per-protocol sensitivity analyses were generally consistent with those observed in the primary ITT analyses, supporting the robustness of the study findings.

The control arm outcomes were concordant with those reported in the IMbrave150 trial,[13,14] in which ATZ/BEV achieved an ORR of approximately 30% and median PFS of 6.8 months. This external consistency underscores the validity of our study population and supports the interpretation that the improvements observed with CDDP+ATZ/BEV reflect a true treatment effect rather than a baseline imbalance.

Although the exploratory subgroup analyses suggested a greater treatment effect in patients with macrovascular invasion and extrahepatic spread, these observations were based on a limited number of patients and should be interpreted cautiously. The findings are consistent with previous reports demonstrating clinical activity of HAIC in patients with vascular invasion; however, the present study was not powered to establish differential treatment effects across subgroups. Therefore, these results should be considered hypothesis-generating and require confirmation in larger prospective studies.

Mechanistically, the benefits of HAIC before systemic therapy can be understood through two pathways. First, HAIC delivers high intrahepatic concentrations of cisplatin, achieving locoregional cytoreduction that translates into improved intrahepatic control, as shown in earlier HAIC studies.[3,4] Second, the synergistic immunological mechanisms are plausible. Local tumor killing induces antigen release and promotes T-cell infiltration, which may augment checkpoint blockade efficacy.[15–19] Similar immune-enhancing effects have been reported following other locoregional therapies, such as TACE and radiofrequency ablation.[20–22] Notably, the observed efficacy in patients with macrovascular invasion and extrahepatic spread is unlikely to be explained solely by a potential abscopal-like effect. In addition to immune modulation, cisplatin HAIC has demonstrated favorable antitumor activity against aggressive intrahepatic disease, including tumors with portal vein invasion. Therefore, improved intrahepatic disease control together with potential immunological enhancement may have contributed to the observed clinical benefit.

The safety profile is also noteworthy. Both cisplatin and bevacizumab are associated with nephrotoxicity,[23–25] and their combination has raised concerns regarding other toxicities.[26–29] However, in our trial, the incidence of renal AEs did not increase in the CDDP+ATZ/BEV arm, and the overall rates of grade 3–4 treatment-related AEs were comparable between the groups. These results suggest that delivering cisplatin via hepatic arterial infusion may reduce systemic exposure and mitigate nephrotoxicity.[30,31] These findings suggest that this regimen is feasible and tolerable; however, confirmation in a larger cohort is required.

This study has some limitations. First, the relatively small sample size limits the precision of the estimates, which likely

contributes to the statistically non-significant difference in ORR despite a clear numerical difference. Second, tumor responses were assessed by investigators without blinded independent central reviews, which may have introduced bias. Third, the OS data remain immature, and a longer follow-up period is needed to clarify whether the PFS and DoR benefits translate into an OS advantage. Although PFS has been proposed as a surrogate endpoint for OS in advanced HCC, the association is not uniform across all trials. Previous trial-level analyses have demonstrated a moderate correlation between PFS and OS,[32] although longer follow-up is still required to determine whether the observed PFS benefit translates into improved OS. Another limitation of this study is the relatively low proportion of patients with BCLC stage C disease compared with previous pivotal systemic therapy trials. Therefore, the study population may not fully represent the broader population of patients with advanced HCC treated with systemic therapy in routine practice.

In conclusion, although the primary endpoint of ORR did not achieve statistical significance, cisplatin HAIC followed by ATZ/BEV significantly improved PFS and was associated with a longer DoR without any additional toxicity compared to ATZ/BEV alone. These findings are hypothesis-generating and warrant confirmation in larger phase III trials before this strategy can be incorporated into routine clinical practice.

Affiliations

[1]*Department of Gastroenterology, Okayama University Hospital, Japan;* [2]*Department of Gastroenterology, Okayama City Hospital, Japan;* [3]*Department of Gastroenterology, Fukuyama City Hospital, Japan;* [4]*Department of Gastroenterology, Okayama Saiseikai General Hospital, Japan;* [5]*Department of Gastroenterology, Japanese Red Cross Okayama Hospital, Japan;* [6]*Department of Gastroenterology, Hiroshima City Hospital, Japan;* [7]*Department of Gastroenterology, Mitoyo General Hospital, Japan;* [8]*Department of Gastroenterology, Sumitomo Besshi Hospital, Japan;* [9]*Department of Gastroenterology, Kurashiki Central Hospital, Japan;* [10]*Department of Gastroenterology, Fukuyama Medical Center, Japan;* [11]*Department of Gastroenterology, Gunma Saiseikai Maebashi Hospital, Maebashi, Japan;* [12]*Division of Gastroenterology, Department of Medicine, Kurume University School of Medicine, Kurume, Japan*

Abbreviations

AE, adverse event; ATZ/BEV, atezolizumab plus bevacizumab; BCLC, Barcelona Clinical Liver Cancer classification; DoR, duration of response; ECOG PS, Eastern Cooperative Oncology Group performance status; HAIC, hepatic arterial infusion chemotherapy; HCC, hepatocellular carcinoma; ITT, intent-to-treat; ORR, objective response rate; OS, overall survival; PD, progressive disease; PFS, progression-free survival; PS, performance status.

Financial support

This study was supported by Nippon Kayaku Co., Ltd.

Conflict of interest

The authors have no competing interests to declare. The funder played no role in study design, data collection, data analysis, data interpretation, or manuscript writing.

Please refer to the accompanying ICMJE disclosure forms for further details.

Authors' contributions

Yasuto Takeuchi and Kazuhiro Nouso designed the study. Masahiko Sue, Nozomi Miyake, Takuya Adachi, Hideki Onishi, Miwa Kawanaka, Akinobu Takaki, Kazuya Kariyama, Tetsuya Yasunaka, Kenji Kuwaki, Junichiro Toshimori, Shota Iwado, Akio Moriya, Hiroaki Hagihara, Hiroyuki Takabatake, Masahiro Sakata, Takeshi Hatanaka, Shigeo Shimose, Takumi Kawaguchi and Yasuto Takeuchi collected and interpreted data. Yasuto Takeuchi had access to all of the data. Yasuto Takeuchi analyzed the data. Motoyuki Otsuka and Yasuto Takeuchi wrote the manuscript.

Data availability

The data that support the findings of this study are not available due to institutional policy and patient confidentiality restrictions. Further enquiries can be directed to the corresponding author.

Statement of ethics

This study protocol was reviewed and approved by Okayama University Certified Review Board, approval number CRB21-006.

Acknowledgements

This study was supported by the Department of Data Science, Center for Innovative Clinical Medicine, Okayama University Hospital, Japan.

Supplementary data

Supplementary data to this article can be found online at https://doi.org/10.1016/j.jhepr.2026.102002.

References

[1] Xin Y, Cao F, Yang H, et al. Efficacy and safety of atezolizumab plus bevacizumab combined with hepatic arterial infusion chemotherapy for advanced hepatocellular carcinoma. Front Immunol 2022 Aug 5;13:929141.

[2] Wang Z, Song S, Zhang L, et al. Hepatic arterial infusion chemotherapy combined with immune checkpoint inhibitors and molecular targeted therapies for advanced infiltrative hepatocellular carcinoma: a single-center experience. Front Immunol 2025 Jan 10;15:1474442.

[3] Ikeda M, Shimizu S, Sato T, et al. Sorafenib plus hepatic arterial infusion chemotherapy with cisplatin for advanced hepatocellular carcinoma: a randomized trial. J Hepatol 2016;64(3):571–578.

[4] Kudo M, Ueshima K, Ikeda M, et al. Hepatic arterial infusion chemotherapy with cisplatin plus sorafenib *vs.* sorafenib alone in advanced hepatocellular carcinoma: a randomized trial. Eur J Cancer 2018;94:1–11.

[5] Luke JJ, Lemons JM, Karrison TG, et al. Safety and clinical activity of pembrolizumab and multisite stereotactic body radiotherapy in patients with advanced solid tumors. J Clin Oncol 2018;36(16):1611–1618.

[6] Hegde PS, Wallin JJ, Mancao C. Predictive markers of anti-VEGF and emerging role of angiogenesis in immune checkpoint inhibitor responses. Nat Rev Clin Oncol 2018;15(6):336–354.

[7] Levine JM, Habib A, Silk M, et al. Abscopal effect with liver-directed therapy: a review of the current literature and future directions. Livers 2024;4(4):601–614.

[8] Eisenhauer EA, Therasse P, Bogaerts J, et al. New response evaluation criteria in solid tumours: revised RECIST guideline (version 1.1). Eur J Cancer 2009;45(2):228–247.

[9] Harris PA, Taylor R, Thielke R, et al. Conde. Research electronic data capture (REDCap) – a metadata-driven methodology and workflow process for providing translational research informatics support. J Biomed Inform 2009 Apr;42(2):377–381.

[10] Harris PA, Taylor R, Minor BL, et al. REDCap Consortium, the REDCap consortium: building an international community of software partners. J Biomed Inform 2019 Jul;95:103208.

[11] Freites-Martinez A, Santana N, Arias-Santiago S, et al. Using the common terminology criteria for adverse events (CTCAE - version 5.0) to evaluate the severity of adverse events of anticancer therapies. Actas Dermosifiliogr (Engl Ed) 2021;112(1):90–92.

[12] Reig M, Forner A, Rimola J, et al. BCLC strategy for prognosis prediction and treatment recommendation: the 2022 update. J Hepatol 2022 Mar;76(3):681–693.

[13] Finn RS, Qin S, Ikeda M, et al. Atezolizumab plus bevacizumab in unresectable hepatocellular carcinoma. N Engl J Med 2020 May 14;382(20):1894–1905.

[14] Salem R, Johnson GE, Kim E, et al. Characterization of response to atezolizumab + bevacizumab versus sorafenib for hepatocellular carcinoma: results from the IMbrave150 trial. Cancer Med 2021;10(18):6079–6090.
[15] Catanzaro E, Beltrán-Visiedo M, Galluzzi L, et al. Immunogenicity of cell death and cancer immunotherapy. Cell Mol Immunol 2025 Jan;22(1):24–39.
[16] Fabian KP, Wolfson B, Hodge JW. From immunogenic cell death to immunogenic modulation: select chemotherapy regimens induce a spectrum of immune enhancing activities in the tumor microenvironment. Front Oncol 2021 Aug 23;11:728018.
[17] Arimoto KI, Miyauchi S, Liu M, et al. Emerging role of immunogenic cell death in cancer immunotherapy. Front Immunol 2024 May 10;15:1390263.
[18] Yu S, Xiao H, Ma L, et al. Reinforcing the immunogenic cell death to enhance cancer immunotherapy. Biochim Biophys Acta Rev Cancer 2023 Sep;1878(5):188946.
[19] Zhu H, Shan Y, Ge K, et al. Oxaliplatin induces immunogenic cell death in hepatocellular carcinoma cells and synergizes with immune checkpoint blockade therapy. Cell Oncol (Dordr) 2020;43:1203–1214.
[20] Young S, Hannallah J, Goldberg D, et al. Friend or Foe? Locoregional therapies and immunotherapies in the current hepatocellular treatment landscape. Int J Mol Sci 2023 Jul 14;24(14):11434.
[21] Brandi N, Renzulli M. The synergistic effect of interventional locoregional treatments and immunotherapy for the treatment of hepatocellular carcinoma. Int J Mol Sci 2023 May 11;24(10):8598.
[22] Luke JJ, Lemons JM, Karrison TG, et al. Safety and clinical activity of pembrolizumab and multisite stereotactic body radiotherapy in patients with advanced solid tumors. J Clin Oncol 2018;36(16):1611–1618.
[23] Miller RP, Tadagavadi RK, Ramesh G, et al. Mechanisms of cisplatin nephrotoxicity. Toxins (Basel) 2010 Nov;2(11):2490–2518.
[24] Hayman SR, Leung N, Grande JP, et al. VEGF inhibition, hypertension, and renal toxicity. Curr Oncol Rep 2012 Aug;14(4):285–294.
[25] Eremina V, Jefferson JA, Kowalewska J, et al. VEGF inhibition and renal thrombotic microangiopathy. N Engl J Med 2008 Mar 13;358(11):1129–1136.
[26] Sandler A, Gray R, Perry MC, et al. Paclitaxel-carboplatin alone or with bevacizumab for non-small-cell lung cancer. N Engl J Med 2006 Dec 14;355(24):2542–2550.
[27] Tewari KS, Sill MS, Penson RT, et al. Bevacizumab for advanced cervical cancer: final overall survival and adverse event analysis of a randomised, controlled, open-label, phase 2 trial (Gynecologic Oncology Group 240). Lancet 2017 Oct 7;390(10103):1654–1663.
[28] Ranpura V, Hapani S, Wu S. Treatment-related mortality with bevacizumab in cancer patients: a meta-analysis. JAMA 2011;305(5):487–494.
[29] Botrel TEA, Clark O, Clark L, et al. Efficacy of bevacizumab (Bev) plus chemotherapy (CT) compared to CT alone in previously untreated locally advanced or metastatic non-small cell lung cancer (NSCLC): systematic review and meta-analysis. Lung Cancer 2011 Oct;74(1):89–97.
[30] Stewart DJ, Benjamin RS, Zimmerman S, et al. Clinical pharmacology of intraarterial cis-diamminedichloroplatinum (II). Cancer Res 1983 Feb;43(2):917–920.
[31] Vexler AM, Mou X, Gabizon AA, et al. Reduction of the systemic toxicity of cisplatin by intra-arterial hepatic route administration for liver malignancies. Int J Cancer 1995 Mar 3;60(5):611–615.
[32] Llovet JM, Montal R, Villanueva A. Randomized trials and endpoints in advanced HCC: role of PFS as a surrogate of survival. J Hepatol 2019;70(6):1262–1277.

Keywords: Hepatocellular carcinoma; Atezolizumab plus bevacizumab; Cisplatin hepatic arterial infusion chemotherapy.
Received 27 March 2026; received in revised form 4 August 2026; accepted 17 August 2026; Available online 29 August 2026

Journal of Hepatology, Volume *8*

Supplemental information

Atezolizumab–bevacizumab with or without cisplatin-based HAIC for unresectable HCC: A randomized phase II trial

Yasuto Takeuchi, Masahiko Sue, Nozomi Miyake, Takuya Adachi, Hideki Onishi, Miwa Kawanaka, Akinobu Takaki, Kazuhiro Nouso, Kazuya Kariyama, Tetsuya Yasunaka, Kenji Kuwaki, Junichi Toshimori, Shota Iwado, Akio Moriya, Hiroaki Hagihara, Hiroyuki Takabatake, Masahiro Sakata, Takeshi Hatanaka, Shigeo Shimose, Takumi Kawaguchi, and Motoyuki Otsuka

Atezolizumab–bevacizumab with or without cisplatin-based HAIC for unresectable HCC: A randomized phase II trial

Yasuto Takeuchi, Masahiko Sue, Nozomi Miyake, Takuya Adachi, Hideki Onishi, Miwa Kawanaka, Akinobu Takaki, Kazuhiro Nouso, Kazuya Kariyama, Tetsuya Yasunaka, Kenji Kuwaki, Junichi Toshimori, Shota Iwado, Akio Moriya, Hiroaki Hagihara, Hiroyuki Takabatake, Masahiro Sakata, Takeshi Hatanaka, Shigeo Shimose, Takumi Kawaguchi, Motoyuki Otsuka

Table of contents

Fig. S1…………………………………………………………………………………2

Fig. S2…………………………………………………………………………………3

Fig. S3…………………………………………………………………………………4

Table S1………………………………………………………………………………5

Supplementary figures

Supplementary Figure 1A

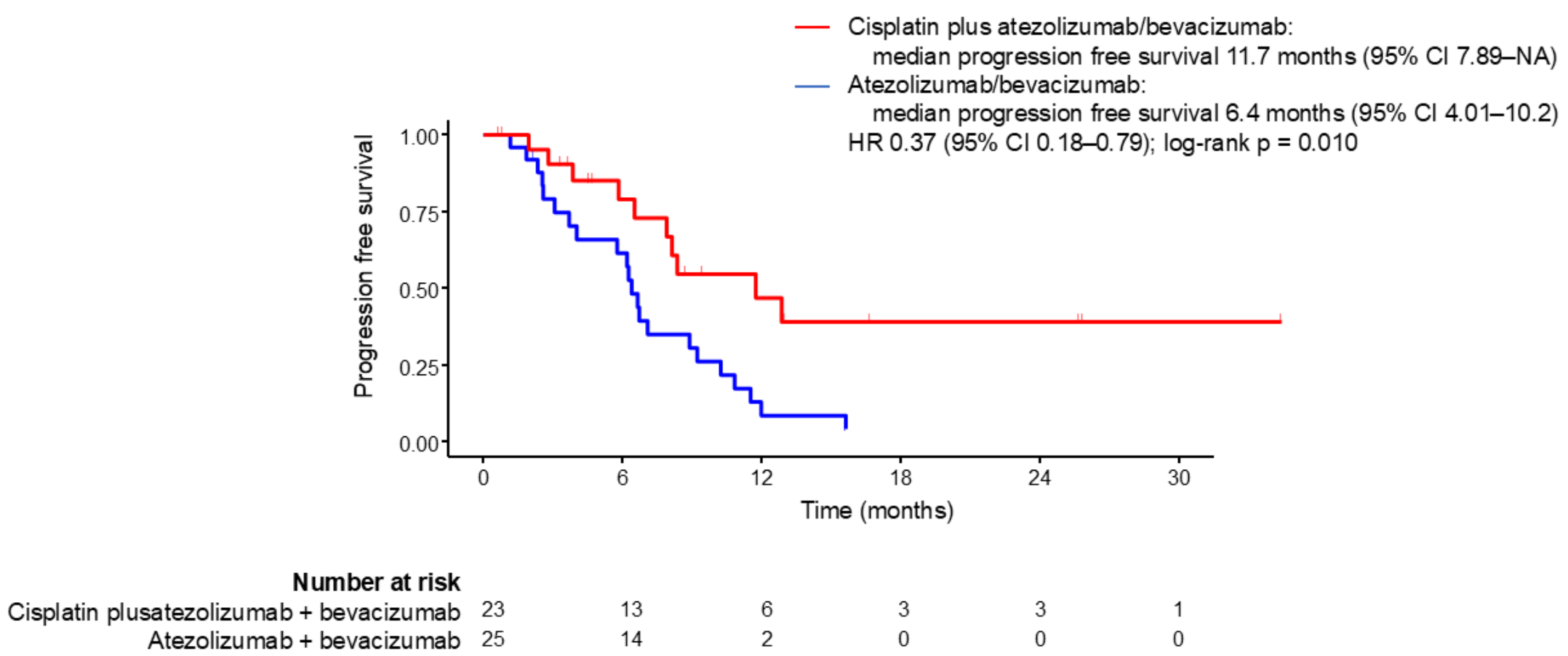

Supplementary Figure 1B

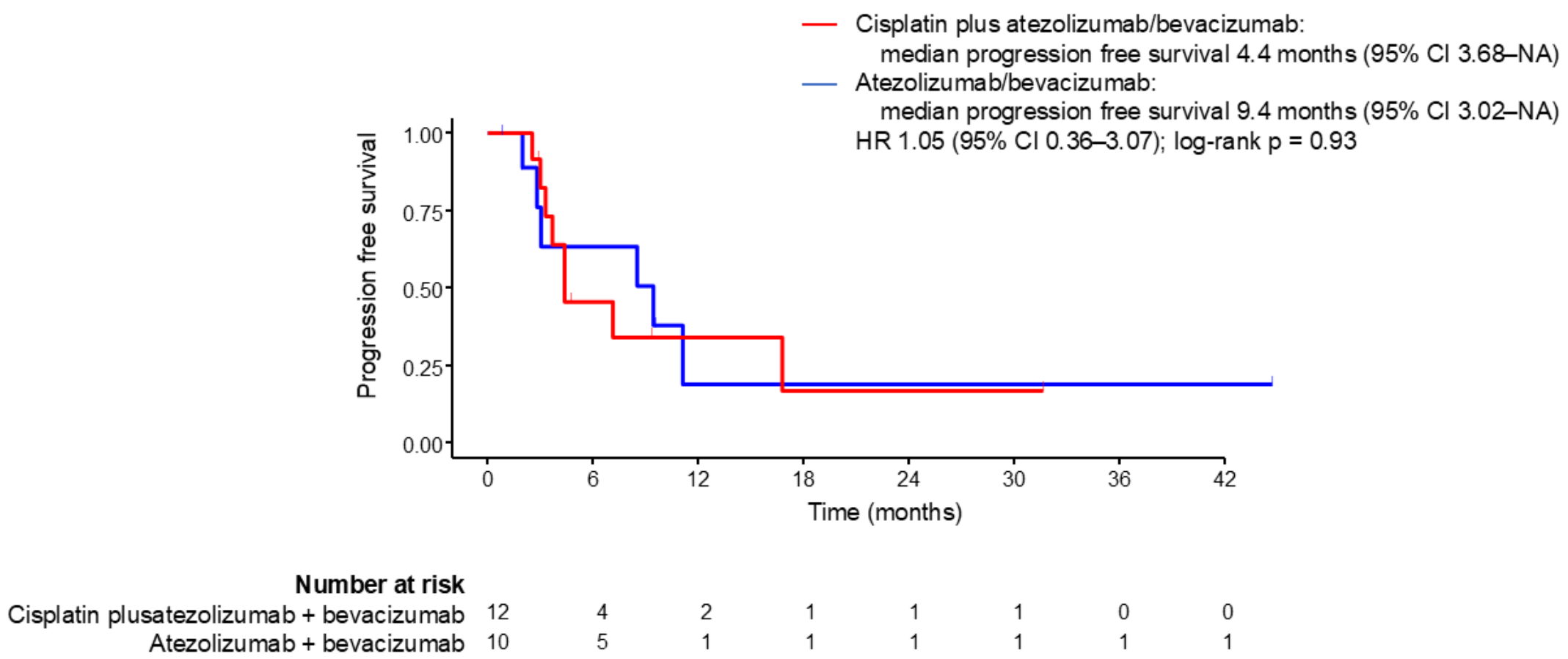

Fig. S1. Overall survival according to treatment group by macrovascular invasion. (A) Kaplan–Meier curves for overall survival comparing CDDP+ATZ/BEV and ATZ/BEV in patients with macrovascular invasion. Median overall survival was 11.7 months in the CDDP+ATZ/BEV group and 6.4 months in the ATZ/BEV group (HR, 0.37; 95% CI, 0.18–0.79; p = 0.010). (B) Kaplan–Meier curves for overall survival

comparing CDDP+ATZ/BEV and ATZ/BEV in patients without macrovascular invasion. Median overall survival was 4.4 months in the CDDP+ATZ/BEV group and 9.4 months in the ATZ/BEV group (HR, 1.05; 95% CI, 0.36–3.07; p = 0.93).

Supplementary Figure 2

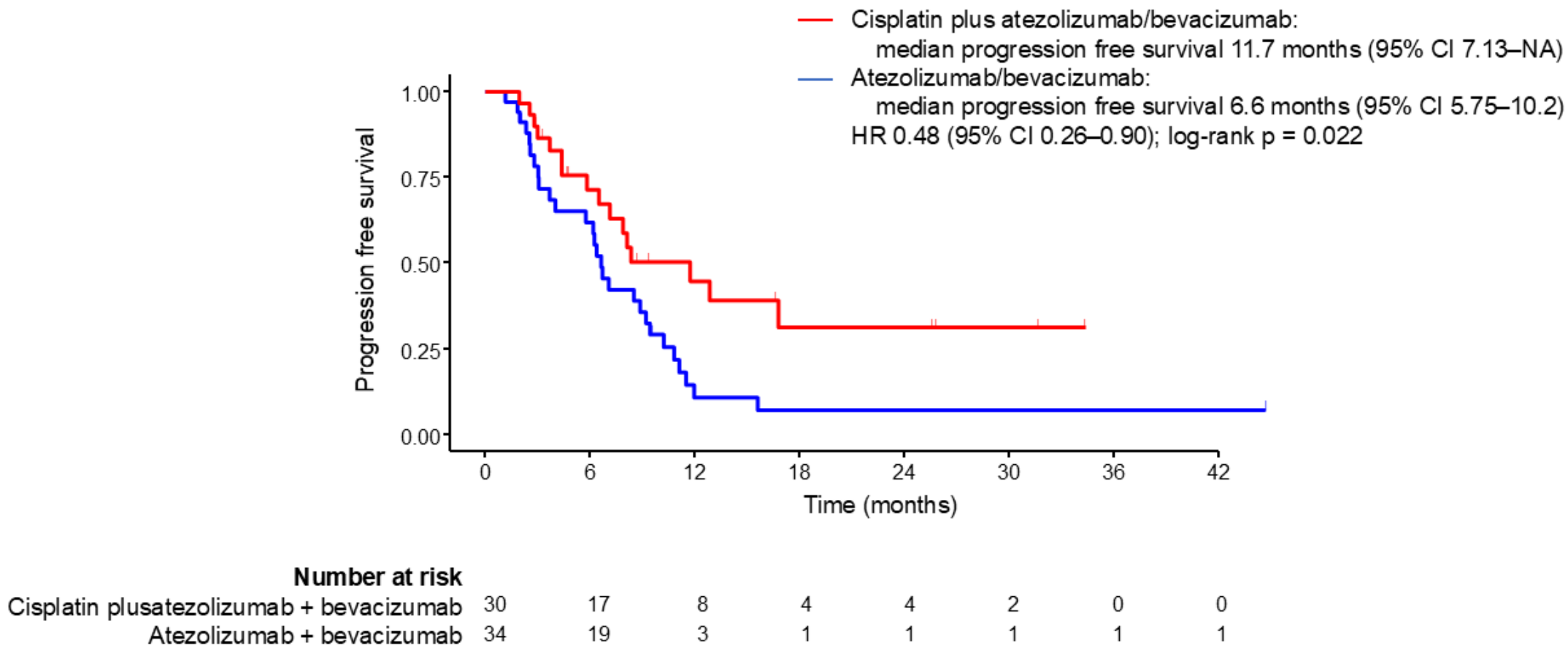

Fig. S2. Kaplan–Meier curves for progression-free survival in the safety/per-protocol population.

Patients who received the assigned treatment were included in this sensitivity analysis. Median progression-free survival was 7.5 months in the CDDP+ATZ/BEV group and 5.7 months in the ATZ/BEV group (hazard ratio, 0.48; 95% confidence interval, 0.26–0.90; p = 0.022).

Supplementary Figure 3

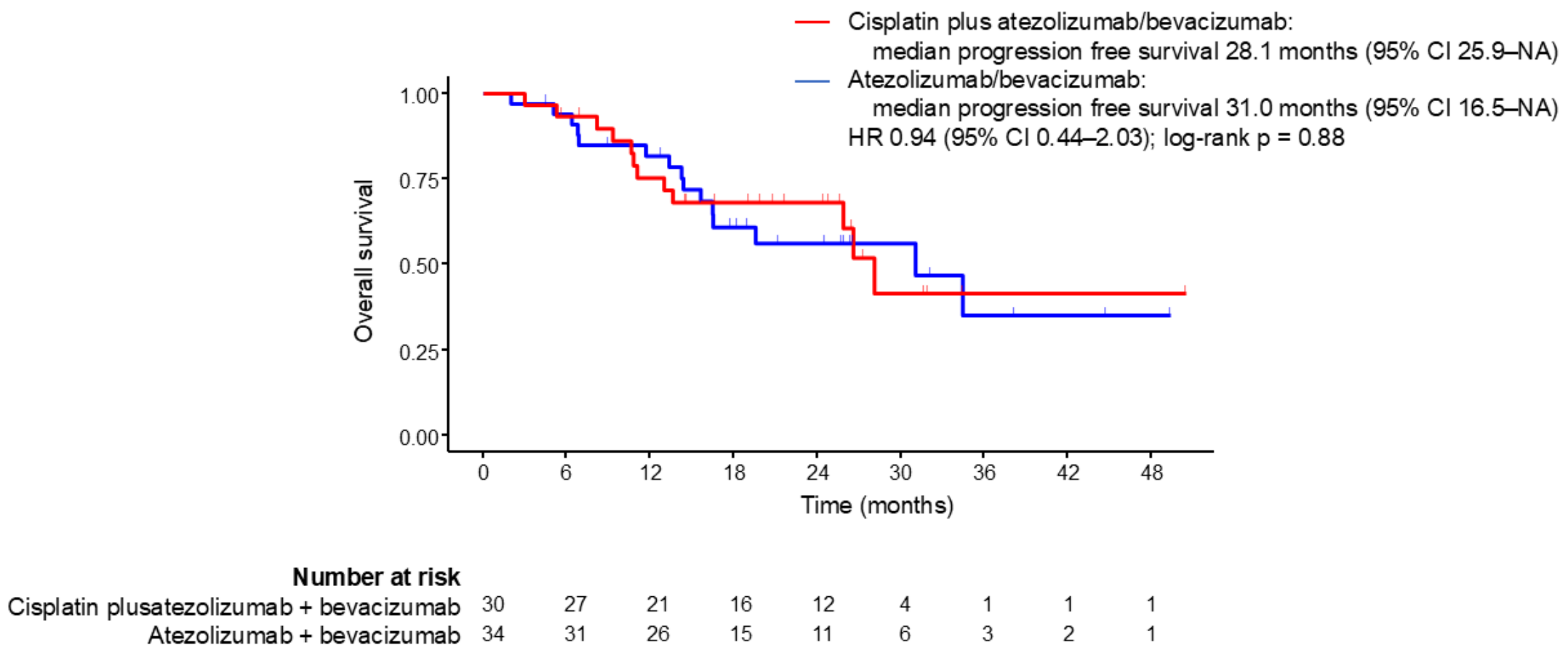

Fig. S3. Overall survival in the safety/per-protocol population.

Kaplan–Meier curves for overall survival comparing CDDP+ATZ/BEV and ATZ/BEV among patients who received the assigned treatment. Median overall survival was 28.1 months in the CDDP+ATZ/BEV group and 31.0 months in the ATZ/BEV group (hazard ratio, 0.94; 95% confidence interval, 0.44–2.03; p = 0.88).

Tables

Table S1: Tumor response outcomes in the per-protocol population

	Cisplatin plus atezolizumab + bevacizumab (n = 30)	Atezolizumab + bevacizumab (n = 34)	*P*-value
Objective response rate*, n (%)	16 (53.3)	10 (29.4)	0.059
Disease control rate*, n (%)	26 (86.7)	24 (70.6)	0.29
Best overall response			
Complete response, n (%)	2 (6.7)	1 (2.9)	
Partial response, n (%)	14 (46.7)	9 (26.5)	
Stable disease, n (%)	10 (33.3)	14 (41.2)	
Progressive disease, n (%)	3 (10.0)	8 (23.5)	
Not evaluable, n (%)	1 (3.3)	2 (5.9)	
Median time to response (IQR), months†	2.99 (2.00–4.89)	2.97 (2.14–5.55)	0.91

*Patients who had confirmed complete response or partial response per Response Evaluation Criteria in Solid Tumors (version 1.1). †Assessed in patients with a confirmed partial or complete response.
