# Supplementary material for: Atezolizumab–bevacizumab with or without cisplatin-based HAIC for unresectable HCC: A randomized phase II trial
Source: JHEP Rep. 2026 Aug 29;8(11):102002. doi: 10.1016/j.jhepr.2026.102002 (PMC13659464; doi:10.1016/j.jhepr.2026.102002)
Supplement: Supplementary file 5 [file mmc4.zip › Clinical Trial/Approval letter.pdf]

Unified Format 4

Reference No.	CRB21-006

Date:2021/09/28

Notification of Review Results

Sponsor
Dr. Takeuchi Yasuto
Okayama University Hospital
(■individual (natural person)／
□Legal Entity ・Organization)

Certified Review Board
Okayama University Certified Review Board Chair
Regional Bureau(s) of Health and Welfare：Chugoku-Shikoku Regional Bureau of Health and Welfare

We would like to notify you regarding the requested review as follows.

The clinical trial number (jRCT No.)			
Scientific Title	Efficacy and safety of Combination therapy of Hepatic Arterial Infusion Chemotherapy and Atezolizumab with Bevacizumab for patients with unresectable advanced Hepatocellular Carcinoma		
Items for Review	■Applicability of the Research conduct (Initial Review Request Form (2021/09/09))<br>□Applicability of the Research continuation<br>□Implementation Plan Amendment (Amendment Review Request Form (YYYY/MM/DD))<br>(□Submission of the Primary Endpoint Report)<br>□Disease, etc. Report<br>(□Pharmaceutical Disease, etc. Report (YYYY/MM/DD))<br>(□Medical Device Disease, etc. or Malfunction Report (YYYY/MM/DD))<br>(□Regenerative Medical Product Disease, etc. or Malfunction Report (YYYY/MM/DD))<br>□Periodic Report (Periodic Report Form (YYYY/MM/DD))<br>□Major Non-compliance (Major Non-compliance Report (YYYY/MM/DD))<br>□Others ( )<br>□Discontinuation of Clinical Research (Notification of Discontinuation (YYYY/MM/DD))<br>□Completion of Clinical Research (Notification of Completion (YYYY/MM/DD))		
Review Division	■Full Board (Date of Board meeting: 2021/09/28)<br>□Simplified review (Date of Board meeting: YYYY/MM/DD)<br>□Urgent review (Date of Board meeting: YYYY/MM/DD)		
Outcome	■Approved	□Disapproval	□Continuous review
Reason(s) for non-approved case			
Comments			
Notes			
