# Supplementary material for: Atezolizumab–bevacizumab with or without cisplatin-based HAIC for unresectable HCC: A randomized phase II trial
Source: JHEP Rep. 2026 Aug 29;8(11):102002. doi: 10.1016/j.jhepr.2026.102002 (PMC13659464; doi:10.1016/j.jhepr.2026.102002)
Supplement: Supplementary file 5 [file mmc4.zip › Clinical Trial/English_ICF_ver1.0.docx]

**Informed Consent Form**

**Safety and Efficacy of Combination Therapy with Hepatic Arterial Infusion Chemotherapy
and Systemic Chemotherapy (Atezolizumab plus Bevacizumab)
for Unresectable Hepatocellular Carcinoma**

*English text-only version translated from the attached Japanese explanatory/consent document.
Figures and tables have been omitted at the user's request.*

Version 1.0 (created July 20, 2021)

Principal Investigator: Yasuto Takeuchi, MD

Center for Innovative Clinical Medicine, Okayama University Hospital

# Introduction

The "clinical research" for which we are asking your participation has been planned and designed by physicians engaged in actual clinical practice in view of its medical necessity and importance. Establishing new treatments through clinical research is one of the missions of physicians, and this can be accomplished only with the cooperation of patients. This study is not a so-called company-sponsored "clinical trial" conducted to investigate the safety and usefulness of a new drug for the purpose of obtaining approval from the Ministry of Health, Labour and Welfare.

In Japan, the incidence of primary liver cancer is high by global standards, and the number of deaths from hepatocellular carcinoma ranks fifth among malignant tumors in this country. When hepatocellular carcinoma is judged to be unresectable because of vascular invasion within the liver or metastasis outside the liver, systemic chemotherapy is recommended, and several drugs are available. At present, treatment using the two anticancer drugs atezolizumab and bevacizumab is considered the first choice. On the other hand, in the Japanese clinical practice guidelines for liver cancer, hepatic arterial infusion chemotherapy using cisplatin (an anticancer drug that damages cancer cells) is also an available option. However, there is no clear evidence regarding the combination of cisplatin-based hepatic arterial infusion therapy with atezolizumab plus bevacizumab. Previously, a study was conducted to confirm the efficacy of combination therapy using hepatic arterial infusion chemotherapy together with sorafenib, an anticancer drug approved in 2009. Although a statistically significant difference was not demonstrated, the results suggested favorable therapeutic efficacy of the combination therapy. The purpose of this study is to confirm the therapeutic effect of combining a cisplatin preparation with atezolizumab plus bevacizumab, which has higher efficacy than sorafenib, and to consider treatment strategies that should be recommended in the future. In addition, similar studies in lung cancer have reported increases in serious adverse events such as proteinuria, hypertension, bleeding events, neutropenia, febrile neutropenia, and treatment-related death. Therefore, another purpose of this study is to confirm safety by investigating the characteristics of adverse events in detail.

Please also note that minors, or patients for whom it is difficult to provide consent based on sufficient understanding on their own, are not eligible for this study. We appreciate your understanding and cooperation.

# 1. Permission from the Head of the Research Institution to Conduct This Study

This clinical research has been approved by the Okayama University Certified Clinical Research Review Board, and the administrator of each participating medical institution (the hospital director) has granted permission for its conduct. The study will be carried out in compliance with the Clinical Research Act. Whether or not to participate in this study is entirely up to you. You will suffer no disadvantage whatsoever if you decide not to participate.

[The table describing the review committee has been omitted in this English text-only version.]

The appropriateness of conducting this study and related matters has been reviewed by the following committee to determine whether the clinical research is ethically and scientifically appropriate.

The standard operating procedures of the Okayama University Certified Clinical Research Review Board, the list of committee members, and summaries of committee deliberations are publicly available on its website and may be viewed freely: http://www.hsc.okayama-u.ac.jp/nintei/index.html

# 2. Study Implementation Structure

This study will be conducted under the following structure.

Principal Investigator
Affiliation: Center for Innovative Clinical Medicine, Okayama University Hospital
Position: Physician
Name: Yasuto Takeuchi

Collaborating research institutions
See separate appendix.

# 3. Purpose and Significance of This Study

The purpose of this study is to administer combination therapy consisting of hepatic arterial infusion chemotherapy using a cisplatin preparation and systemic chemotherapy using atezolizumab plus bevacizumab to patients with unresectable hepatocellular carcinoma, and to evaluate its efficacy and safety. Clarifying these points may lead to the development of a new treatment option.

# 4. Methods and Duration of This Study

## 4-1) Methods

This study will enroll patients who have been diagnosed with unresectable hepatocellular carcinoma at our hospital or at a collaborating research institution and who consent to participate in this study. There are also several other conditions for participation. Please refer to "5. Reasons Why You Were Selected as a Research Participant" below for details.

Study flow

(Screening period)
For those who provide consent to participate, we will first conduct screening. This includes reviewing clinical information such as age and diagnosis, as well as information from imaging studies and blood tests previously performed, to confirm whether all criteria necessary for formal participation in the study are met.

(Registration and treatment period)
Once the above eligibility has been confirmed, you will be registered in the study. At that time, you will be assigned to one of two treatment approaches: either administration of atezolizumab plus bevacizumab after administration of the cisplatin preparation, or administration of atezolizumab plus bevacizumab without administration of the cisplatin preparation. The assignment will be determined with a 1:1 probability. Neither your attending physician nor you will know in advance which treatment method will be assigned, and it cannot be chosen. Once the method has been determined, hepatic arterial infusion therapy with the cisplatin preparation will be performed during hospitalization. Within 4 weeks after the arterial infusion therapy, atezolizumab plus bevacizumab will likewise be administered during hospitalization. The hospitalization period after administration of atezolizumab plus bevacizumab is expected to be approximately 1 to 3 days, although this may vary depending on your condition. After discharge, atezolizumab plus bevacizumab will be repeated every 3 weeks and will be continued at the discretion of the attending physician until the disease worsens or treatment must be discontinued because of adverse effects. Please refer to the later explanation for details of the study schedule, including the treatment schedule.

Treatment schedule
[The treatment schedule figure has been omitted in this English text-only version.]

If severe adverse effects occur due to hepatic arterial infusion chemotherapy with the cisplatin preparation, administration of atezolizumab plus bevacizumab may be postponed or discontinued. In addition, if severe adverse effects occur with atezolizumab plus bevacizumab or if the effect is insufficient, treatment may be temporarily withheld or discontinued.

During the treatment period, there are no restrictions on treatment for adverse effects (combination therapy or supportive care). However, cancer treatments other than those specified in this study, such as chemotherapy, radiotherapy, hormonal therapy, immunotherapy, hyperthermia, or surgery, are prohibited. This is for patient safety and to allow proper evaluation of efficacy. Nevertheless, there may be situations in which the use of these drugs or treatments becomes necessary to protect your health. In such cases, continued participation in the study may become difficult. Please ask your attending physician for details.

(Observation period)
If administration of atezolizumab plus bevacizumab is completed or discontinued, your condition will be observed for study purposes for 12 months from that date. Please refer to the table below for the specific items to be observed.

[The schedule table has been omitted in this English text-only version.]

## 4-2) Planned participation period

This study will be conducted from the date of publication in the public database established by the Ministry of Health, Labour and Welfare, the Japan Registry of Clinical Trials (jRCT) (https://jrct.niph.go.jp/search), until December 31, 2025.

The planned participation period for each patient is approximately 2 years in total, consisting of a screening period of about 4 weeks, a treatment period of about 8 months (which varies among individuals), and an observation period of 12 months.

## 4-3) Types of study drugs

The following medicinal products will be used in this study:
- Cisplatin preparation: IA-call(R) for arterial infusion, 50 mg
- Atezolizumab: Tecentriq(R), 1200 mg
- Bevacizumab: Avastin(R), 100 mg

## 4-4) Method of administration of the study drugs

If you participate in this study, you will be assigned to one of the following groups:
- IA-call(R) for arterial infusion administration group
- IA-call(R) for arterial infusion non-administration group

In either group, you will receive Tecentriq(R) 1200 mg and Avastin(R) 15 mg/kg every 3 weeks.

IA-call(R) for arterial infusion will be administered into the hepatic artery through a catheter. Tecentriq(R) and Avastin(R) will be administered by intravenous infusion. The start date of administration will be determined by the attending physician according to your condition and other factors. Intravenous infusion of Tecentriq(R) and Avastin(R) will be repeated every 3 weeks from the first treatment day. At each visit, your symptoms and other information will be checked using a treatment diary, so please keep the diary accurately.

## 4-5) Planned number of participants

This is a multicenter collaborative study to be conducted in a total of 100 patients with unresectable hepatocellular carcinoma at 11 institutions. At our hospital, approximately 40 patients are planned to be enrolled.

## 4-6) Cases in which your participation in the study may be discontinued

Even if you agree to participate, your participation may be discontinued in the following situations. If participation is discontinued, the attending physician will explain the reason and how the data collected up to that point will be handled. Please be assured that even after discontinuation, the attending physician will continue to treat you sincerely.

1. If you request withdrawal from participation or revoke your consent.
2. If a pre-existing illness (such as a comorbidity) worsens and it is considered difficult for you to continue participating in the study.
3. If adverse effects or other events that may affect continued participation occur after the start of the study.
4. If the study as a whole is discontinued for some reason.
5. If you intentionally fail to comply with the matters described in "4-7) Matters you are asked to observe if you participate in the study."
6. If the investigator otherwise judges that discontinuation of the study is appropriate.

The following situations are anticipated as possible reasons for discontinuing the study as a whole:
- When important information regarding the quality, safety, or efficacy of the drugs used in this study becomes available.
- When recruitment of participants is difficult and it is judged to be extremely difficult to achieve the planned number of cases.
- When the objective of the study has been achieved before the planned number of cases or planned study period has been reached.
- When the Certified Clinical Research Review Board instructs changes to the implementation plan or related documents, and it is judged difficult to accept such instructions.

## 4-7) Matters you are asked to observe if you participate in the study

If you are currently visiting another hospital, please inform us of the name of that hospital, your diagnosis, and any medications you are using. Please also inform us if you are taking any medicines purchased at a pharmacy or elsewhere. This information is important for conducting the study safely. In addition, if you are visiting another hospital, we may inform that hospital that you are participating in this study. Thank you for your understanding.

## 4-8) If new information becomes available

If, during the course of the study, new information becomes available that may affect your safety or your willingness to continue participation, we will inform you promptly. Whether or not to continue participating in this study is entirely your own decision.

# 5. Reasons Why You Were Selected as a Research Participant

Patients who participate in this study will be selected according to the following criteria.

## 5-1) Inclusion criteria (main requirements for participation)

1. You are 20 years of age or older at the time consent is obtained.
2. Either sex is eligible.
3. Your activities of daily living are either completely unrestricted, or you are ambulatory and able to perform light work or seated work although strenuous physical activity is restricted.
4. You have been diagnosed with unresectable hepatocellular carcinoma.
5. You have never received hepatic arterial infusion chemotherapy using IA-call(R) for arterial infusion.
6. Treatment with Tecentriq(R) and Avastin(R) is planned.
7. Intrahepatic lesions are confirmed.
8. After receiving sufficient explanation regarding participation in this study, you are able to provide written consent of your own free will based on adequate understanding.

## 5-2) Exclusion criteria (main reasons you cannot participate)

1. Poor liver function (to be judged specifically by the principal investigator or subinvestigator).
2. Two or more prior treatments with transarterial chemoembolization, except when performed as part of curative treatment in combination with surgery or radiofrequency ablation.
3. Allergy to CT contrast media.
4. Poor renal function (to be judged specifically by the principal investigator or subinvestigator).
5. Allergy to any component of IA-call(R) for arterial infusion.
6. Allergy to any component of Tecentriq(R).
7. Allergy to any component of Avastin(R).
8. History of hemoptysis.
9. Presence of an autoimmune disease, or a history of chronic or recurrent autoimmune disease.
10. Presence of interstitial pneumonia.
11. Intra-abdominal inflammation or an unhealed surgical wound.
12. Brain metastasis.
13. A hereditary disease associated with increased bleeding tendency.
14. Serious heart disease such as severe heart failure.
15. Pregnant women or women who may be pregnant.
16. Any other case in which the principal investigator or subinvestigator judges participation to be inappropriate, for example because introduction of chemotherapy is difficult.

We are asking eligible patients who meet these criteria to participate in this study.

# 6. Burdens, Foreseeable Risks, and Expected Benefits of Participating in This Study

## (1) Expected benefits

One potential benefit of participating in this study is that, if the additional effect of IA-call(R) for arterial infusion is obtained, the combination therapy may improve treatment efficacy.

## (2) Expected disadvantages

If you participate in this study, the following disadvantages are possible. The physicians in charge of this study will always check your health condition and will conduct the study with the utmost care to minimize the burden on you.

1. If hepatic arterial infusion chemotherapy is performed, an additional hospitalization period of approximately 5 to 10 days will be required compared with standard chemotherapy alone; however, the duration of hospitalization required for each course of hepatic arterial infusion chemotherapy or systemic chemotherapy itself will not differ from that in usual clinical practice.
2. Events such as those described below under "(3) Foreseeable adverse effects/complications" may occur.

Even if you participate in this study, compared with ordinary clinical care, there is no increase in the number of outpatient visits, length of hospital stay, number of examinations, or number of blood draws.

## (3) Foreseeable adverse effects/complications

The drugs used in this study - IA-call(R) for arterial infusion, Tecentriq(R), and Avastin(R) - have been reported in previous studies to cause the following adverse effects.

[Detailed adverse-event frequency lists from the package inserts and domestic clinical studies have been translated conceptually here, but long tabular/numerical formatting has been kept in prose because tables were omitted.]

IA-call(R) for arterial infusion
In domestic clinical trials in patients with hepatocellular carcinoma, major adverse effects included decreased appetite, nausea/vomiting, pyrexia, malaise, leukopenia, neutropenia, thrombocytopenia, elevated AST, decreased hemoglobin, elevated ALT, increased serum bilirubin, decreased serum albumin, decreased total serum protein, elevated LDH, increased BUN, and increased blood creatinine. Other reported adverse reactions include acute renal failure, bone marrow suppression such as pancytopenia, thrombocytopenia, leukopenia, neutropenia, anemia, shock, anaphylaxis, hepatic dysfunction, jaundice, fulminant hepatitis, hepatobiliary disorders, myocardial infarction, angina pectoris, congestive heart failure, arrhythmia, pulmonary tuberculosis, hearing impairment, papilledema, retrobulbar optic neuritis, cortical blindness, cerebral infarction, hemolytic uremic syndrome, hemolytic anemia, interstitial pneumonia, syndrome of inappropriate secretion of antidiuretic hormone, gastrointestinal bleeding, peptic ulcer, gastrointestinal perforation, acute pancreatitis, hyperglycemia, worsening diabetes mellitus, and rhabdomyolysis.

Tecentriq(R)
In domestic clinical trials in patients with hepatocellular carcinoma, major adverse effects included hypertension, proteinuria, fatigue, increased AST, pruritus, infusion-related reaction, diarrhea, increased ALT, and decreased appetite. Other reported adverse reactions include interstitial lung disease, hepatic dysfunction/hepatitis, colitis, severe diarrhea, pancreatitis, type 1 diabetes mellitus, thyroid dysfunction including hypothyroidism, hyperthyroidism, and thyroiditis, adrenal dysfunction including adrenal insufficiency, pituitary dysfunction including hypophysitis and hypopituitarism, encephalitis, meningitis, neurologic disorders such as peripheral neuropathy, polyneuropathy, and Guillain-Barre syndrome, myasthenia gravis, severe skin disorders such as erythema multiforme, toxic epidermal necrolysis, and Stevens-Johnson syndrome, renal dysfunction including acute kidney injury, renal failure, tubulointerstitial nephritis, and nephritis, myositis, rhabdomyolysis, myocarditis, hemophagocytic syndrome, infusion reaction, and febrile neutropenia.

Avastin(R)
In domestic clinical trials in patients with hepatocellular carcinoma, major adverse effects included hypertension, proteinuria, fatigue, increased AST, pruritus, infusion-related reaction, diarrhea, increased ALT, and decreased appetite. Other reported adverse reactions include shock, anaphylaxis, gastrointestinal perforation, fistula, delayed wound healing including wound dehiscence and postoperative hemorrhage, bleeding, thromboembolism such as cerebral infarction, transient ischemic attack, angina pectoris, myocardial infarction, cerebrovascular accident, and cerebral ischemia, hypertensive encephalopathy, hypertensive crisis, reversible posterior leukoencephalopathy syndrome, nephrotic syndrome, bone marrow suppression including pancytopenia when combined with other antineoplastic agents, neutropenia, leukopenia, anemia, thrombocytopenia, infection, congestive heart failure, interstitial pneumonia, thrombotic microangiopathy, and arterial dissection.

In addition, adverse effects that are not yet known may also occur. During the study period, we will carefully observe you for the appearance of adverse effects and other symptoms. If any adverse effect occurs, appropriate treatment will be provided. If you notice any symptoms or physical condition different from usual, please record them accurately in the treatment diary provided to you and inform the study physician immediately.

# 7. Freedom to Participate and Withdrawal of Consent

Participation in this study is entirely voluntary. If you understand this study and are willing to participate of your own free will, please sign the separate "Clinical Research Consent Form." Even after giving consent, you may withdraw it at any time. In that case, please notify the attending physician verbally and sign the separate "Consent Withdrawal Form." Whether or not you consent, and even if you later withdraw your consent, you will suffer no disadvantage whatsoever in your medical care. If you withdraw your consent, information concerning you that has been obtained in the study will be discarded; however, please understand that after the study results have already been analyzed, it may be difficult to discard such information.

# 8. How Information About This Study Will Be Made Public

Before this study begins, it will be registered in advance in the database of the Clinical Research Submission and Disclosure System (jRCT), and after the study is completed the results will be made public. In addition, the study results are planned to be presented in jRCT, medical journals, academic meetings, and similar settings.

# 9. Access to the Study Protocol and Related Materials

If you would like to know about the study plan or related materials for this study, we can provide such information except for personal information of other patients and matters that could interfere with the conduct of the study as a whole. Please ask the study physician if you wish to obtain or review such materials.

# 10. Handling of Personal Information

Information extracted from your medical records and similar sources will be managed using study-specific code numbers and other measures, with sufficient care taken so that your name and other identifying information are not known to third parties. This is called anonymization.

The results obtained in this study may be published in medical journals, academic meetings, and similar venues, but your name and other identifying information will never be disclosed, and your privacy will be protected.

If the information obtained in this study is to be used for future research, a new review by the ethics committee will be sought. In such a case as well, sufficient care will be taken to protect your privacy.

This is a collaborative study conducted with other institutions. Therefore, your information will be provided to and compiled at Okayama University Hospital; however, your name and other identifying information will not be included, and the information will be sent with full consideration for your privacy.

# 11. Methods for Storage and Disposal of Information

Information obtained in this study will be used for this study and will be stored in a locked location at each institution (at the lead institution, Okayama University Hospital, in the Department of Gastroenterology office) until five years have elapsed from the date on which the end of the study is reported. Thereafter, the information will be completely destroyed, for example by shredding paper records or deleting electronic files.

# 12. Research Funding and Conflicts of Interest

This study is funded by Nippon Kayaku Co., Ltd.

In addition, the researchers have declared conflicts of interest in advance to the Okayama University Certified Clinical Research Review Board, and it has been confirmed that there are no problems with their management. Furthermore, by periodically reporting the progress of the study to the Okayama University Certified Clinical Research Review Board, fairness with respect to any interests related to this study will be maintained.

# 13. Financial Burden and Honoraria

The costs necessary for this study will generally be covered within ordinary insured medical care; however, if hepatic arterial infusion chemotherapy is combined, a difference from the cost of ordinary chemotherapy may arise because of extension of the hospitalization period and similar factors.

Please also understand that no honorarium, travel reimbursement, or similar payment will be provided even if you participate in this study.

# 14. Other Treatment Options if You Do Not Participate in This Study

If you do not participate in this study, the most appropriate treatment will be selected according to your condition from among established conventional therapies in line with the latest clinical practice guidelines for liver cancer.

# 15. Care After the End of the Study

After this study has ended, the study physician will responsibly provide what is considered the most appropriate medical care, taking into account the findings obtained in this study as well.

# 16. Handling of Important Findings Obtained Through the Study

If, as a result of conducting this study, (1) important findings concerning your health or (2) other important findings are obtained, we may inform you of such findings.

# 17. Compensation for Health Damage

This clinical research is scientifically planned and will be conducted carefully. However, if during participation in this study you notice any symptoms or physical condition different from usual, please inform the study physician immediately. Appropriate measures and treatment will be provided without delay. In such cases, treatment will be provided using your health insurance in the same manner as ordinary medical care.

Because the treatment investigated in this study is used within the scope of existing regulatory approval, there is no special compensation such as payment of medical expenses, medical allowances, or compensation money for health damage that occurs. Please make your decision about participation with full understanding of this point.

However, if serious health damage occurs due to adverse effects despite your receiving treatment correctly in accordance with the instructions of the study physician, you may be eligible to apply for benefits under the Relief System for Adverse Drug Reactions. Please note that if a causal relationship between the health damage and the drug used is denied, or if the health damage occurred because of intentional misconduct or gross negligence on the part of the patient, relief benefits may not be granted or may be limited.

# 18. Secondary Use of Specimens/Information and Provision to Other Research Institutions

In principle, specimens and information obtained in this study will be used for this study and will be safely stored in anonymized form for five years from the date on which completion of the study is reported. If, in the future, a new study is planned and the specimens or information obtained in this study are to be used for medical research, a new study protocol will be submitted, approval by the ethics committee will be obtained, and either your consent will be sought again or you will be given an opportunity to refuse to be included as a research subject through public disclosure of information.

# 19. Monitoring and Audit

To confirm that this study is being conducted properly while your human rights are protected, persons involved in this clinical research - such as the ethics review committee of this university, staff members of our hospital, personnel from regulatory authorities, and others designated by the principal investigator - may review your medical records, including your chart. This is called monitoring or audit. However, there is no risk that information related to your privacy, such as your address, name, or telephone number, will be leaked externally. In addition, no report or other document will identify you personally.

# 20. Ownership of Intellectual Property Rights

No intellectual property rights are expected to arise from this study.

# 21. Consultation Contact for Patients and Others

If there is anything you would like to know about this study or anything that worries you, please feel free to consult the study physician.

700-8558
2-5-1 Shikata-cho, Kita-ku, Okayama
Center for Innovative Clinical Medicine, Okayama University Hospital
Principal Investigator: Lecturer, Yasuto Takeuchi
Contact information:
Department of Gastroenterology office: 086-235-7219 (weekdays 9:00-17:00)
West Ward 6F, Department of Gastroenterology: 086-235-6724 (nights and holidays)

Our hospital also has the following complaints and consultation desk:
Clinical Trial and Clinical Research Consultation Desk
General Patient Support Center, Okayama University Hospital
Tel: 086-235-7744 (weekdays 8:30-17:00)
E-mail: iscps@okayama-u.ac.jp

## Please read this document carefully and discuss it fully with your physician before deciding whether to participate.
