# Supplementary material for: Atezolizumab–bevacizumab with or without cisplatin-based HAIC for unresectable HCC: A randomized phase II trial
Source: JHEP Rep. 2026 Aug 29;8(11):102002. doi: 10.1016/j.jhepr.2026.102002 (PMC13659464; doi:10.1016/j.jhepr.2026.102002)
Supplement: Supplementary file 5 [file mmc4.zip › Clinical Trial/English_ICF_ver2.0.docx]

English ICF (Revised Version)

*Explanatory and Consent Document for Clinical Research
(Text Only; Tables/Figures Omitted)*

Version 2.0 (Prepared on September 25, 2023)

**To all patients,**

Explanation Regarding the Study entitled "Evaluation of the Safety and Efficacy of Combination Therapy with Hepatic Arterial Infusion Chemotherapy and Systemic Chemotherapy (Atezolizumab plus Bevacizumab) for Unresectable Hepatocellular Carcinoma"

Principal Investigator

Center for Innovative Clinical Medicine, Okayama University Hospital

Title: Physician

Name: Yasuto Takeuchi

# Introduction

The clinical research for which we are asking your participation is planned and conducted by physicians engaged in actual clinical practice in light of medical necessity and importance. Establishing new treatments through clinical research is an important mission of physicians and can only be achieved with the cooperation of patients. This study is not a so-called company-sponsored clinical trial conducted to investigate the safety and usefulness of a new drug for the purpose of obtaining approval from the Ministry of Health, Labour and Welfare.

In Japan, the incidence of primary liver cancer is high by global standards, and deaths due to hepatocellular carcinoma rank fifth among all malignant neoplasms in this country. When hepatocellular carcinoma is judged to be unresectable because of vascular invasion within the liver or metastasis outside the liver, systemic chemotherapy is recommended, and several agents are available. At present, a regimen using the two anticancer agents atezolizumab and bevacizumab is considered the first-choice treatment. On the other hand, the Japanese Clinical Practice Guidelines for Liver Cancer also allow hepatic arterial infusion chemotherapy using cisplatin, an anticancer drug that damages cancer cells, in which the drug is administered directly through a catheter into the artery feeding the liver tumor. However, there is no clear evidence regarding the combination of cisplatin-based hepatic arterial infusion chemotherapy with atezolizumab plus bevacizumab. Previously, a study evaluated the efficacy of combination therapy consisting of hepatic arterial infusion chemotherapy and sorafenib, an anticancer agent approved in 2009, and although no statistically significant difference was observed, the results suggested favorable therapeutic efficacy for the combination. The purpose of this study is to confirm the therapeutic effect of combining cisplatin with atezolizumab plus bevacizumab, which has greater efficacy than sorafenib, and to explore a treatment strategy that may be recommended in the future. In addition, in a similar study in lung cancer, increases in serious adverse events such as proteinuria, hypertension, bleeding events, neutropenia, febrile neutropenia, and treatment-related death have been reported. Therefore, another objective of this study is to confirm safety by investigating the characteristics of adverse events in detail.

Please note that this study does not include minors or patients for whom it is difficult to provide consent based on sufficient understanding. We appreciate your understanding and cooperation.

# 1. Permission from the Head of the Research Institution to Conduct This Study

This clinical research has been approved by the Okayama University Certified Review Board for Clinical Research, and the administrator of the implementing medical institution (the hospital director) has granted permission for its conduct. The study will also be conducted in compliance with the Clinical Research Act. Whether or not you participate in this study is entirely up to your own free will. You will suffer no disadvantage whatsoever if you choose not to participate.

Whether this study should be conducted and related matters have been reviewed by the following committee to determine whether the clinical research is ethically and scientifically appropriate.

The operating procedures of the Okayama University Certified Review Board for Clinical Research, the roster of committee members, and the outline of committee deliberations are publicly available on its website and may be viewed freely.

# 2. Study Organization

This study will be conducted under the following structure:

• Principal Investigator: Yasuto Takeuchi, Physician, Center for Innovative Clinical Medicine, Okayama University Hospital

• Participating institutions: See separate appendix

# 3. Purpose and Significance of the Study

The purpose of this study is to administer a combination of hepatic arterial infusion chemotherapy using a cisplatin formulation and systemic chemotherapy with atezolizumab plus bevacizumab to patients with unresectable hepatocellular carcinoma, and to evaluate its efficacy and safety. Clarifying these points may lead to the development of a new treatment option.

# 4. Methods and Study Period

## 4-1) Methods

Eligible participants are patients diagnosed with unresectable hepatocellular carcinoma at our hospital or a participating institution who agree to participate in this study. There are several additional eligibility requirements. Please see Section 5, "Reasons Why You Were Selected as a Study Participant," for details.

Study Flow

(Screening period) For those who consent to participate, screening will first be performed. This includes confirmation of clinical information (such as age and diagnosis) and information from previous imaging and blood tests to determine whether all requirements for formal participation in the study are met.

(Registration and treatment period) Once the above has been confirmed, you will be registered in the study. At that time, you will be allocated to one of two methods: administration of atezolizumab plus bevacizumab after administration of a cisplatin formulation, or administration of atezolizumab plus bevacizumab without the cisplatin formulation. The allocation is determined with a probability of 1:1; however, neither your attending physician nor you can know or choose which method will be assigned. Once the assigned method has been determined, you will be hospitalized and undergo hepatic arterial infusion therapy with the cisplatin formulation. Within 4 weeks after arterial infusion therapy, you will again be hospitalized and receive atezolizumab plus bevacizumab. The hospitalization period after atezolizumab plus bevacizumab administration is expected to be approximately 1 to 3 days, although this may vary depending on your condition. After discharge, administration of atezolizumab plus bevacizumab will be repeated every 3 weeks and will be continued at the discretion of the attending physician until the disease worsens or treatment must be discontinued because of adverse events. For details of the study schedule including administration, please refer to the explanation provided later.

Dosing schedule: omitted in this English text-only version.

If severe adverse events occur due to hepatic arterial infusion chemotherapy with the cisplatin formulation, administration of atezolizumab plus bevacizumab may be postponed or discontinued. In addition, if severe adverse events occur with atezolizumab plus bevacizumab, or if the treatment effect is insufficient, treatment may be interrupted or discontinued.

During the treatment period, there are no restrictions on treatment for adverse events (concomitant therapy or supportive care). However, cancer treatments other than those specified in this study, such as chemotherapy, radiotherapy, hormone therapy, immunotherapy, hyperthermia, or surgery, are prohibited. This is for patient safety and in order to evaluate efficacy appropriately. However, there may be situations in which the use of such medications or treatments is necessary to protect your health. In such cases, continued participation in the study may become difficult. Please ask your study physician for details.

(Observation period) If administration of atezolizumab plus bevacizumab is completed or discontinued, your condition will be observed for study purposes for 12 months from that date. The specific observation items were listed in the original schedule table, which has been omitted in this text-only English version.

## 4-2) Planned Participation Period

This study will be conducted from the date of publication in the public database established by the Ministry of Health, Labour and Welfare (Japan Registry of Clinical Trials: jRCT) until December 31, 2026.

For each patient, the planned participation period is approximately 2 years in total, consisting of an approximately 8-week screening period, an approximately 8-month treatment period (which may vary among individuals), and a 12-month observation period.

## 4-3) Types of Study Drugs

The following medicinal products will be used in this study:

• IA-call for arterial infusion, 50 mg (cisplatin formulation)

• Tecentriq, 1200 mg (atezolizumab)

• Avastin, 100 mg (bevacizumab)

## 4-4) Method of Administration of the Study Drugs

If you participate in this study, you will be assigned to one of the following groups:

• IA-call administration group

• Non-IA-call administration group

In either group, you will receive Tecentriq 1200 mg and Avastin 15 mg/kg every 3 weeks.

IA-call will be administered into the hepatic artery using a catheter. Tecentriq and Avastin will be administered by intravenous infusion, and the start date of administration will be determined by the attending physician according to your condition and other factors. Intravenous infusion of Tecentriq and Avastin will be repeated every 3 weeks beginning from the first administration date. Your symptoms and other findings will be checked using a treatment diary at each visit, so please record the diary accurately.

## 4-5) Planned Number of Participants

This is a multicenter study conducted at 13 institutions involving a total of 67 patients with unresectable hepatocellular carcinoma. At our hospital, approximately 30 patients are planned to participate.

## 4-6) Cases in Which Your Participation May Be Discontinued

Even if you agree to participate, your participation in the study may be discontinued in the following cases. If discontinuation becomes necessary, the study physician will explain the reason and how the data collected up to that point will be handled. Please be assured that even after discontinuation, your physician will continue to treat you sincerely.

1. If you request withdrawal from the study or revoke your consent.

2. If a pre-existing disease (such as a comorbidity) worsens and it is considered difficult for you to continue participating in this study.

3. If adverse events or other issues occur after the start of the study that are considered likely to affect continuation of participation.

4. If, for some reason, the study as a whole is discontinued.

5. If you intentionally fail to comply with the matters described in Section 4-7, "What You Need to Observe If You Participate in the Study."

6. If the study physician otherwise judges that discontinuation of your participation is appropriate.

Examples of situations in which the study as a whole may be discontinued include the following:

• Serious information is obtained regarding the quality, safety, or efficacy of the medicinal products used in this study.

• Recruitment of study participants is difficult and it is judged to be extremely unlikely that the planned number of cases can be achieved.

• The objective of the study is achieved before the planned number of cases or planned study period is reached.

• The Certified Review Board for Clinical Research instructs modifications to the implementation plan or other study documents, and it is judged difficult to accept those modifications.

## 4-7) What You Need to Observe If You Participate in the Study

If you are currently visiting another hospital, please inform us of that hospital, your diagnosis, and the medications you are using. Please also inform us of any medicines you purchase and use from pharmacies or elsewhere. This is important for conducting the study safely. In addition, if you are visiting another hospital, please understand that we may inform that hospital that you are participating in this study.

## 4-8) If New Information Is Obtained

If, during the study period, new information becomes available that may affect your safety or your willingness to continue participating in the study, we will inform you promptly. Whether you continue participation in this study will be entirely up to your own free will.

# 5. Reasons Why You Were Selected as a Study Participant

Patients who participate in this study are selected according to the following criteria.

## 5-1) Inclusion Criteria (Main Requirements for Participation)

1. You are 20 years of age or older at the time consent is obtained.

2. Sex is not restricted.

3. Your activities of daily living are either completely unimpaired, or although strenuous physical activity is limited, you are ambulatory and able to perform light work or sedentary tasks.

4. You have been diagnosed with unresectable hepatocellular carcinoma.

5. You have never received hepatic arterial infusion chemotherapy using IA-call.

6. Treatment with Tecentriq and Avastin is planned.

7. Intrahepatic lesions are confirmed.

8. After receiving sufficient explanation regarding participation in this study and understanding it sufficiently, you have provided written consent of your own free will.

## 5-2) Exclusion Criteria (Main Conditions Under Which Participation Is Not Possible)

1. Poor liver function (to be judged specifically by the principal investigator or subinvestigators).

2. Allergy to CT contrast media.

3. Poor renal function (to be judged specifically by the principal investigator or subinvestigators).

4. Allergy to any component of IA-call.

5. Allergy to any component of Tecentriq.

6. Allergy to any component of Avastin.

7. History of hemoptysis.

8. Presence of an autoimmune disease or a history of chronic or recurrent autoimmune disease.

9. Complication of interstitial pneumonia.

10. Inflammation in the abdominal cavity or an unhealed surgical wound.

11. Brain metastasis.

12. A hereditary disease associated with a tendency to bleed.

13. Severe heart disease such as severe heart failure.

14. Pregnancy or the possibility of being pregnant.

15. Any other reason for which the principal investigator or subinvestigators judge participation to be inappropriate, such as difficulty initiating chemotherapy.

We are asking patients who meet these criteria to participate in this study.

# 6. Burden, Foreseeable Risks, and Benefits Associated with Participation in This Study

## (1) Expected Benefits

As a possible benefit of participating in this study, if the addition of IA-call provides an additive effect, combination therapy may improve treatment efficacy.

## (2) Expected Disadvantages

If you participate in this study, the following disadvantages may occur. The physicians in charge of this study will always monitor your health condition and conduct the study with the utmost care to minimize your burden.

1. If hepatic arterial infusion chemotherapy is performed, an additional hospitalization period of approximately 5 to 10 days will be necessary in addition to ordinary chemotherapy. However, the hospitalization period required for each of hepatic arterial infusion chemotherapy and chemotherapy itself is not different from that in ordinary treatment.

2. Events such as those described below in "(3) Anticipated Side Effects/Complications" may occur.

Even if you participate in this study, compared with usual medical care, the number of outpatient visits, the amount of time spent in the hospital, the number of tests, and the number of blood collections will not increase.

## (3) Anticipated Side Effects/Complications

It has been reported in previous studies that the following side effects may occur with the study drugs used in this research: IA-call, Tecentriq, and Avastin.

[IA-call]

1) Domestic clinical trials in patients with hepatocellular carcinoma: Major adverse reactions reported include loss of appetite (79.8%), nausea/vomiting (76.0%), fever (63.5%), malaise (26.9%), leukopenia (78.8%), neutropenia (77.2%), thrombocytopenia (76.9%), elevated AST/GOT (56.7%), decreased hemoglobin (51.0%), elevated ALT/GPT (45.2%), increased serum bilirubin (36.5%), decreased serum albumin (31.7%), decreased total serum protein (29.8%), elevated LDH (27.9%), and elevated BUN (25.0%) and blood creatinine (21.2%).

2) Other adverse reactions: Acute renal failure (frequency unknown); myelosuppression such as pancytopenia, including thrombocytopenia (23.2%), leukopenia (20.8%), neutropenia (18.7%), anemia (less than 1%), and pancytopenia (frequency unknown); thrombocytopenia (1.4%); shock and anaphylaxis (frequency unknown); liver dysfunction (35.4%); jaundice (less than 1%); fulminant hepatitis (frequency unknown); hepatobiliary disorders (less than 1%); myocardial infarction (less than 1%); angina pectoris (frequency unknown); congestive heart failure (frequency unknown); arrhythmia (less than 1%); pulmonary tuberculosis (less than 1%); hearing impairment (less than 1%); papilledema, retrobulbar optic neuritis, and cortical blindness (all frequency unknown); cerebral infarction (frequency unknown); hemolytic uremic syndrome (frequency unknown); hemolytic anemia (frequency unknown); interstitial pneumonia (frequency unknown); syndrome of inappropriate antidiuretic hormone secretion (frequency unknown); gastrointestinal bleeding, peptic ulcer, and gastrointestinal perforation (all frequency unknown); acute pancreatitis (frequency unknown); hyperglycemia (less than 1%); worsening of diabetes mellitus (frequency unknown); and rhabdomyolysis (frequency unknown).

[Tecentriq]

1) Domestic clinical trials in patients with hepatocellular carcinoma: Major adverse reactions reported include hypertension in 78 cases (23.7%), proteinuria in 62 cases (18.8%), fatigue in 50 cases (15.2%), increased AST in 46 cases (14.0%), pruritus in 43 cases (13.1%), infusion-related reactions in 36 cases (10.9%), diarrhea in 34 cases (10.3%), increased ALT in 34 cases (10.3%), and decreased appetite in 33 cases (10.0%).

2) Other adverse reactions: Interstitial lung disease (2.7%); liver dysfunction and hepatitis, including increased AST (5.5%), increased ALT (5.5%), increased alkaline phosphatase (1.9%), increased gamma-GTP (0.8%), and increased bilirubin (1.4%); colitis (1.2%); severe diarrhea (1.4%); pancreatitis (0.1%); type 1 diabetes mellitus (0.1%); thyroid dysfunction such as hypothyroidism (7.5%), hyperthyroidism (2.9%), and thyroiditis (0.4%); adrenal dysfunction such as adrenal insufficiency (0.4%) and acute adrenocortical insufficiency (less than 0.1%); pituitary dysfunction such as hypophysitis (0.3%) and hypopituitarism (0.1%); encephalitis (0.1%); meningitis (0.1%); neurologic disorders such as peripheral neuropathy (3.1%), polyneuropathy (0.2%), and Guillain-Barre syndrome (0.1%); myasthenia gravis (frequency unknown); severe skin disorders such as erythema multiforme (0.2%), toxic epidermal necrolysis (TEN) (frequency unknown), and Stevens-Johnson syndrome (frequency unknown); renal dysfunction such as acute kidney injury (0.4%), renal failure (0.4%), tubulointerstitial nephritis (0.3%), and nephritis (0.2%); myositis (0.1%); rhabdomyolysis (0.1%); myocarditis (0.1%); hemophagocytic syndrome (less than 0.1%); infusion reaction (2.8%); and febrile neutropenia (2.8%).

[Avastin]

1) Domestic clinical trials in patients with hepatocellular carcinoma: Major adverse reactions reported include hypertension in 78 cases (23.7%), proteinuria in 62 cases (18.8%), fatigue in 50 cases (15.2%), increased AST in 46 cases (14.0%), pruritus in 43 cases (13.1%), infusion-related reactions in 36 cases (10.9%), diarrhea in 34 cases (10.3%), increased ALT in 34 cases (10.3%), and decreased appetite in 33 cases (10.0%).

2) Other adverse reactions: Shock and anaphylaxis (1.9%); gastrointestinal perforation (0.9%); fistula (0.3%); delayed wound healing, including wound dehiscence due to delayed healing (0.5%) and postoperative bleeding (0.4%); bleeding (19.3%); thromboembolism such as cerebral infarction (0.2%), transient ischemic attack (0.1%), angina pectoris (0.1%), myocardial infarction (less than 0.1%), cerebrovascular accident (frequency unknown), and cerebral ischemia (frequency unknown); hypertensive encephalopathy (frequency unknown); hypertensive crisis (frequency unknown); reversible posterior leukoencephalopathy syndrome (less than 0.1%); nephrotic syndrome (less than 0.1%); myelosuppression, including pancytopenia (less than 0.1%) when used in combination with other antineoplastic agents, neutropenia (24.5%), leukopenia (24.3%), anemia (8.7%), and thrombocytopenia (10.4%); infection (10.0%); congestive heart failure (less than 0.1%); interstitial pneumonia (0.4%); thrombotic microangiopathy (frequency unknown); and arterial dissection (less than 0.1%).

Other, as yet unknown, side effects may also occur.

During the study period, we will carefully observe whether symptoms such as adverse events appear. If adverse events occur, appropriate treatment will be provided. If you experience any symptoms different from usual or any physical discomfort, please record them accurately in the treatment diary provided to you and inform the study physician immediately.

# 7. Freedom to Participate and Withdrawal of Consent

Participation in this study is based on your own free will. If you understand this study and agree to participate of your own free will, please sign the separate Clinical Research Consent Form. Even after giving consent once, you may withdraw it at any time. In that case, please inform your physician orally and sign the separate Withdrawal of Consent Form. You will not be disadvantaged in your medical care in any way even if you do not consent or if you withdraw your consent. If you withdraw consent, the information obtained about you in this study will be discarded; however, please note that after analysis of the study results, disposal may be difficult.

# 8. How Information About the Study Will Be Disclosed

Before implementation of the study, the clinical research implementation plan and study summary will be registered in advance in the database of the Clinical Research Implementation Plan and Research Summary Disclosure System (jRCT), and the results will be made public after the study is completed. In addition, the study results are planned to be presented in the jRCT database, in medical journal articles, and at academic meetings.

# 9. Access to or Review of the Study Protocol and Materials Concerning Study Methods

If you would like to know about the study plan or related materials for this study, information can be provided to you except for personal information of other patients and matters that would interfere with the study as a whole. Please ask the study physician if you wish to do so.

# 10. Handling of Personal Information

Information extracted from your medical records and related data will be managed using study-specific code numbers and similar methods, with sufficient care taken so that third parties cannot identify your name or other personal information. This is called anonymization.

The results obtained in this study may be published in medical journals or presented at academic meetings, but your name and other identifying information will never be disclosed, and your privacy will be protected.

If the information obtained in this study is to be used for future research, a new review by the ethics committee will be sought. In that case as well, sufficient care will be taken to protect your privacy.

This study is a joint study with other institutions. Therefore, your information will be provided to and aggregated at Okayama University Hospital; however, your name and other identifying information will not be included, and the information will be sent with sufficient consideration for your privacy.

# 11. Method of Storage and Disposal of Information

The information obtained in this study will be used for this study and will be stored in a locked location at each institution (at the Department of Gastroenterology at Okayama University Hospital, the coordinating institution) until five years have passed from the date on which the end of the study is reported. Thereafter, the information will be completely discarded by shredding documents and deleting electronic files.

# 12. Research Funding and Conflicts of Interest

This study is conducted with research funding from Nippon Kayaku Co., Ltd.

In addition, the researchers have declared conflicts of interest in advance to the Okayama University Certified Review Board for Clinical Research, and it has been confirmed that there is no problem in their management. By regularly reporting the progress of the study to the Okayama University Certified Review Board for Clinical Research, fairness regarding any interests related to this study will be maintained.

# 13. Financial Burden and Compensation

The costs necessary for this study will be covered within ordinary insured medical care; however, if hepatic arterial infusion chemotherapy is combined, differences from the costs of ordinary chemotherapy may arise because of prolonged hospitalization or additional inpatient treatment.

Please also note that even if you participate in this study, no honorarium, travel expenses, or similar payments will be provided.

# 14. Other Treatment Options If You Do Not Participate in This Study

In accordance with the latest Clinical Practice Guidelines for Liver Cancer (treatment guide), the most appropriate treatment will be selected from among conventional therapies according to your condition.

# 15. Care After the End of the Study

After this study has ended, the study physician will responsibly provide the medical care considered most appropriate, including consideration of the results obtained from this study.

# 16. Handling of Important Findings Obtained in Connection with the Study

If, in connection with the conduct of the study, either (1) important findings concerning your health or (2) other important findings are obtained, you may be informed of them.

# 17. Compensation for Health Damage

This clinical research will be scientifically planned and conducted with caution; however, if you experience any symptoms different from usual or any physical discomfort while participating in this study, please inform the study physician immediately. Appropriate measures and treatment will be provided promptly. In such a case, treatment will also be provided using your health insurance in the same way as ordinary medical care.

In addition, because the treatment examined in this study uses drugs within their approved indications, there is no special compensation such as medical expenses, medical allowances, or compensation money for health damage that may occur. Please fully understand this point when deciding whether to participate in the study.

However, if serious health damage occurs due to side effects or similar causes despite your receiving treatment properly in accordance with the study physician's instructions, you may be eligible to apply for relief benefits under the Adverse Drug Reaction Relief System. Please note that if a causal relationship between the health damage and the medicinal product used is denied, or if the health damage arises due to intentional action or gross negligence on the part of the patient, such relief benefits may not be available or may be limited.

# 18. Secondary Use of Specimens/Information and Provision to Other Research Institutions

Specimens and information obtained in this study will, in principle, be used for this study and will be safely stored in anonymized form for five years from the date on which the completion of the study is reported. If a new study is planned in the future and the specimens or information obtained in this study are to be used for medical research, a new study protocol will be submitted, approval from the ethics committee will be obtained, and either your consent will be sought again or an opportunity will be provided for study subjects to refuse participation through public disclosure of information.

# 19. Monitoring and Audit

In order to confirm that this study is being conducted properly while your human rights are protected, persons involved in this clinical research (such as the ethics review committee of this university, staff of this hospital, persons in charge from regulatory authorities, and other persons designated by the principal investigator) may review your medical records and similar information. This is called monitoring or audit. However, there is no concern that information relating to your privacy (such as your address, name, or telephone number) will be leaked outside. In addition, such information will not be used in reports or otherwise in a way that identifies you personally.

# 20. Ownership of Intellectual Property Rights

No intellectual property rights or similar rights are expected to arise from this study.

# 21. Response to Consultations from Patients and Others

If there is anything you would like to know about this study or if you have any concerns, please feel free to consult the study physician.

700-8558 2-5-1 Shikata-cho, Kita-ku, Okayama City, Okayama

Center for Innovative Clinical Medicine, Okayama University Hospital

Principal Investigator: Lecturer, Yasuto Takeuchi

Contact:

Department of Gastroenterology office: 086-235-7219 (weekdays 9:00-17:00)

Department of Gastroenterology, West Ward 6F: 086-235-6724 (nights, weekends, and holidays)

In addition, this hospital has established the following contact point for complaints and consultations:

Okayama University Hospital General Patient Support Center, Consultation Desk for Clinical Trials and Clinical Research

Contact: 086-235-7744 (weekdays 8:30-17:00)

E-mail: iscps@okayama-u.ac.jp
