# Supplementary material for: Atezolizumab–bevacizumab with or without cisplatin-based HAIC for unresectable HCC: A randomized phase II trial
Source: JHEP Rep. 2026 Aug 29;8(11):102002. doi: 10.1016/j.jhepr.2026.102002 (PMC13659464; doi:10.1016/j.jhepr.2026.102002)
Supplement: Supplementary file 5 [file mmc4.zip › Clinical Trial/English_Protocol_ver1.0.docx]

Study Protocol

Safety and Efficacy of Combination Therapy with Hepatic Arterial Infusion Chemotherapy and Systemic Chemotherapy (Atezolizumab plus Bevacizumab) for Unresectable Hepatocellular Carcinoma

*English text-only version translated from the attached Japanese protocol. Figures and tables have been omitted at the user's request.*

**Principal Investigator**

Yasuto Takeuchi, MD

Center for Innovative Clinical Medicine, Okayama University Hospital

2-5-1 Shikata-cho, Kita-ku, Okayama 700-8558, Japan

Phone: 086-235-7219

Fax: 086-225-5991

**Study Secretariat**

Center for Innovative Clinical Medicine, Okayama University Hospital

Secretariat Contact: Yasuto Takeuchi

2-5-1 Shikata-cho, Kita-ku, Okayama 700-8558, Japan

Phone: 086-235-7219 (weekdays 9:00-17:00)

086-235-6724 (weekday evenings, weekends, and holidays)

Fax: 086-225-5991

**Planned Study Period**

From the date of publication in the jRCT after approval by the Certified Clinical Research Review Board until December 31, 2025.

Version 1.0 created on July 20, 2021

# 1. Title of the Study

Safety and Efficacy of Combination Therapy with Hepatic Arterial Infusion Chemotherapy and Systemic Chemotherapy (Atezolizumab plus Bevacizumab) for Unresectable Hepatocellular Carcinoma.

# 2. Study Organization (including the names of participating institutions and investigators)

This study will be conducted under the following structure.

Principal Investigator: Yasuto Takeuchi, MD, Physician, Center for Innovative Clinical Medicine, Okayama University Hospital, 2-5-1 Shikata-cho, Kita-ku, Okayama 700-8558, Japan. Phone: 086-235-7219.

Contact Office: Study Secretariat, Center for Innovative Clinical Medicine, Okayama University Hospital, Yasuto Takeuchi, 2-5-1 Shikata-cho, Kita-ku, Okayama 700-8558, Japan. Phone: 086-235-7219 (weekdays 9:00-17:00); 086-235-6724 (weekday evenings, weekends, and holidays). Fax: 086-225-5991.

Monitoring Officer: Shigeru Horiguchi, Associate Professor, Department of General Medicine in the Southwestern Area of Okayama Prefecture (Kasaoka), Okayama University Hospital.

Participating collaborative institutions: see separate appendix.

# 3. Background, Objectives, and Significance of the Study

In Japan, the incidence of primary liver cancer is high by global standards, and deaths from hepatocellular carcinoma (HCC) rank fifth among all cancer deaths in the country.1) Treatment for HCC is generally selected in accordance with the treatment algorithm specified in the Clinical Practice Guidelines for HCC.2) Under this algorithm, when a patient is judged to have unresectable disease due to vascular invasion or extrahepatic metastasis, systemic chemotherapy with molecular targeted agents or related drugs is recommended for patients with good performance status (PS) and preserved hepatic reserve. For unresectable HCC, sorafenib was approved and reimbursed in 2009, followed by regorafenib in 2017, lenvatinib in 2018, ramucirumab in 2019, and atezolizumab (an immune checkpoint inhibitor) plus bevacizumab (an angiogenesis inhibitor) and cabozantinib in 2020.

As noted above, multiple agents are currently available for unresectable HCC. At the same time, hepatic arterial infusion chemotherapy (HAIC) is also positioned as a treatment option in the same algorithm, and reports have shown antitumor shrinkage with response rates ranging from 0% to 71%, although results vary.2) Currently, HAIC mainly consists of cisplatin monotherapy; however, the timing of its introduction has not been standardized, and clear evidence regarding combination use with systemic chemotherapy is lacking. In this context, the phase II SCOOP-2 trial compared cisplatin HAIC followed by sorafenib with sorafenib alone and showed a numerically favorable response rate in the cisplatin-first HAIC group (14.3% vs. 9.1%), although the difference was not statistically significant.3) Another phase II trial comparing sorafenib plus cisplatin HAIC with sorafenib alone showed a significantly prolonged median survival time in the combination arm, and numerically better response rates in the combination arm (21.7% vs. 7.3%), again without a statistically significant difference in response rate.4)

At present, atezolizumab plus bevacizumab is recommended as systemic chemotherapy. The reported response rate with sorafenib was 12%, whereas the response rate with atezolizumab plus bevacizumab was higher at 27%.5) By combining cisplatin, a cytotoxic anticancer agent, with an immune checkpoint inhibitor and an angiogenesis inhibitor, a higher response rate than with existing treatment modalities may be expected, as has been reported in the treatment of lung cancer and cervical cancer.6)7) On the other hand, meta-analytic data have reported an increased incidence of serious adverse events with cisplatin plus angiogenesis inhibitors, including proteinuria, hypertension, bleeding events, neutropenia, febrile neutropenia, and treatment-related death.8) Accordingly, this study aims to investigate in detail the characteristics of adverse events associated with the combination regimen and to evaluate not only efficacy but also safety, thereby informing future treatment recommendations.

# 4. Study Methods

## (1) Study type and design

Open-label, randomized, two-arm comparative study.

## (2) Study outline (see study flowchart)

Eligibility will be confirmed against the inclusion and exclusion criteria, and written informed consent will be obtained from each study participant. After pre-registration examinations (screening tests), eligibility will be finally confirmed and the participant will be registered. Study treatment will then be initiated according to the allocation result. After the prespecified intervention, intergroup comparison of the response rate, which is the study outcome, will be performed.

## (3) Planned study participation period for each participant (intervention period at the participant level)

Intervention period: from the time informed consent is obtained until disease progression or until a discontinuation criterion is met.

Planned participation period: approximately 2 years.

(Screening period: approximately 4 weeks; intervention period: treatment induction and maintenance until disease progression or until a discontinuation criterion is met [average approximately 8 months anticipated]; observation period: 12 months.)

## (4) Overview of the investigational medicinal products/medical devices and details of the intervention

1) Overview

*[Tables describing the details of cisplatin, atezolizumab, and bevacizumab have been omitted in this English text-only version.]*

2) Intervention details

*[IA-call administration group]*

After hepatic arterial infusion of IA-call for arterial infusion (65 mg/m2), Tecentriq intravenous infusion 1200 mg and Avastin intravenous infusion 15 mg/kg will be administered within 2 weeks (up to 4 weeks is allowable) after the HAIC treatment. Tecentriq and Avastin will be administered repeatedly every 3 weeks until discontinuation is judged necessary because of tumor progression, adverse events, or other reasons.

A deviation of within +/-5% between the calculated dose and the actual administered dose will be allowed.

*[Non-IA-call administration group]*

Tecentriq intravenous infusion 1200 mg and Avastin intravenous infusion 15 mg/kg will be administered. They will be repeated every 3 weeks until discontinuation is judged necessary because of tumor progression, adverse events, or other reasons.

A deviation of within +/-5% between the calculated dose and the actual administered dose will be allowed.

Dosing schedule:

*[The dosing schedule table has been omitted in this English text-only version.]*

Criteria for treatment interruption / dose reduction / discontinuation:

a. IA-call for arterial infusion

Dose reduction and treatment interruption will be performed in accordance with the proper use guide for IA-call for arterial infusion.

*[The renal function-based dose adjustment table has been omitted in this English text-only version.]*

b. Tecentriq and Avastin

Dose reduction and treatment interruption will be performed in accordance with the proper use guides for Tecentriq and Avastin.

The grade of adverse events will be defined according to CTCAE v5.0.

When multiple adverse events occur simultaneously, the strictest applicable action among the criteria below will be taken.

*[The detailed adverse-event management tables for interstitial pneumonitis, hepatic dysfunction/hepatitis, colitis/severe diarrhea, pancreatitis, type 1 diabetes mellitus, thyroid dysfunction, adrenal dysfunction, pituitary dysfunction, encephalitis, neurologic disorders, myasthenia gravis, skin disorders, renal dysfunction, myositis/rhabdomyolysis, myocarditis, infusion reaction, and other events have been omitted in this English text-only version.]*

## (5) Management of study drugs

The cisplatin preparation, atezolizumab, and bevacizumab used in this study are already approved drugs, and they will be appropriately stored and managed in accordance with the approved product information.

## (6) Rules regarding concomitant medications (therapies), etc.

1) Concomitant medications (therapies)

a. Recommended concomitant/supportive therapy

The following concomitant and supportive therapies are recommended. Failure to administer them will not be regarded as a protocol deviation.

1. Management of febrile neutropenia (FN)

For therapeutic use of G-CSF at the onset of FN, refer to section d, Granulocyte-Colony Stimulating Factor.

2. Nausea and vomiting

Antiemetics should be administered proactively in accordance with the 'Guidelines for Proper Use of Antiemetics' published by the Japanese Society of Clinical Oncology. If oral intake is markedly reduced, fluids and electrolytes should be supplemented.

3. Anemia and decreased platelet count

When anemia (hemoglobin <=8.0 g/dL) or thrombocytopenia (platelet count <=2.0 x 10^4 /mm3) is observed, transfusion should be performed as appropriate.

b. Permitted concomitant/supportive therapy

1. Bone-modifying agents such as anti-RANKL antibodies and bisphosphonates, drugs for comorbidities such as hypertension and diabetes mellitus, and symptomatic medications such as morphine for pain may be used concomitantly.

2. Palliative radiotherapy outside the abdomen, such as radiotherapy for bone metastases at risk of fracture, is permitted. Interruption of protocol treatment is not mandatory during palliative radiotherapy.

3. Steroid treatment or prophylactic steroid administration for allergic reactions and similar conditions is permitted.

4. Use of an inactivated influenza vaccine is permitted; however, encephalitis after vaccination during immunotherapy has been reported, and the attending physician should carefully consider the clinical necessity.

5. Use of a COVID-19 vaccine is permitted; however, the attending physician should carefully consider the clinical necessity.

6. Other supportive therapies intended for symptom control of complications and adverse events are permitted, including gabapentin or Kampo medicines for peripheral neuropathy (including prophylactic use), and acetaminophen, diphenhydramine, famotidine, and similar agents for infusion reactions.

c. Non-permitted concomitant/supportive therapy

1. During protocol treatment, concomitant anticancer therapies such as radiotherapy, hormone therapy other than steroids, and immunotherapy other than the protocol treatment must not be administered.

d. Granulocyte-colony stimulating factor (G-CSF)

1. Primary prophylactic use of G-CSF (*)

Because the risk of FN with this treatment is less than 10%, primary prophylactic use of G-CSF is not mandatory in accordance with the 'Guidelines for Proper Use of G-CSF, 2013 Edition' and the 'JSMO Clinical Practice Guidelines for Febrile Neutropenia (FN).' Primary prophylaxis with G-CSF should not be used solely because the patient is elderly; however, it is permitted when the attending physician judges that the patient is at high risk of FN after considering risk factors other than age.

2. Secondary prophylactic use of G-CSF (**)

When FN occurred in the previous course, secondary prophylactic G-CSF administration is recommended from the next course onward. However, failure to administer it will not be regarded as a protocol deviation.

G-CSF should be administered in accordance with the approved dosage and administration.

Start timing: (1) at least 24 hours after completion of anticancer chemotherapy; (2)-(4) when the neutrophil count is expected to fall below 1,000/mm3.

Discontinuation timing (except item (1)): discontinue when the neutrophil count has passed its nadir and reaches >=5,000/mm3; or when the neutrophil count has recovered to >=2,000/mm3, there are no symptoms suggestive of infection, and the safety of the patient is judged to be secured based on the response to the administered drug.

3. Therapeutic use of G-CSF

G-CSF should be administered in accordance with the approved dosage and administration. However, in patients with risk factors for severe worsening such as pneumonia or organ dysfunction, the timing and appropriateness of administration should be carefully considered.

Start timing: when fever (in principle, >=38.0 C) is observed with a neutrophil count <1,000/mm3; or when a neutrophil count <500/mm3 is observed.

Discontinuation timing: discontinue when the neutrophil count has passed its nadir and reaches >=5,000/mm3; or when the neutrophil count has recovered to >=2,000/mm3, there are no symptoms suggestive of infection, and the patient's safety is judged to be secured based on the response to the administered drug.

(*) Administration of G-CSF for the purpose of preventing FN or prolonged neutropenia before they occur during anticancer drug treatment.

(**) Administration of G-CSF for the purpose of preventing recurrence of FN or prolonged neutropenia after they have once occurred during anticancer drug treatment.

2) Provisions regarding prohibited concomitant drugs (therapies)

1. Prohibited concomitant use

The following drugs and therapies that are considered likely to affect this study are prohibited throughout the study period.

## (1) Other antineoplastic treatment

Chemotherapy, molecular targeted therapy, immunotherapy other than protocol treatment, antibody therapy, radiotherapy (except where radiotherapy for vascular invasion or palliative radiotherapy outside the abdomen is permitted), hyperthermia, surgery (however, conversion to curative treatment such as hepatectomy, radiofrequency ablation, or transarterial chemoembolization after tumor shrinkage/reduction is permitted), etc.

## (2) Investigational drugs and unapproved drugs

## (7) Case registration and allocation method

The principal investigator at each site or a subinvestigator will register and allocate cases according to the following procedures.

1) Patients who meet all inclusion criteria and none of the exclusion criteria will be regarded as eligible, and written informed consent will be obtained.

2) The principal investigator or subinvestigator will enter the necessary information into the EDC, and allocation will be performed according to age, sex, hepatic reserve, and disease stage.

3) Based on the allocation result, the corresponding study treatment (atezolizumab, bevacizumab, with or without cisplatin preparation) will be administered.

4) If consent withdrawal, discontinuation, dropout, or a similar event occurs, it must be promptly reported to the principal investigator.

## (8) Measures after completion of the study

After completion of this study, the principal investigator or subinvestigator will provide the medical care considered most appropriate for each study participant, taking into account the findings obtained from this study.

## (9) Use of information from other institutions, etc.

In this study, information will be collected from the investigators' own institutions and from other institutions and analyzed.

1) Method of information collection: EDC will be used in this study. Data should be entered promptly after completion of each visit.

The contents entered into the EDC will not include personally identifying information such as the participant's name or date of birth.

2) Information obtained from study participants, etc.: there are no plans to provide such information to other research institutions.

# 5. Observation and Examination Items

## (1) Participant background information

Age, sex, height, body weight, Performance Status (PS), present illness, comorbidities, past history, lifestyle history (smoking history, alcohol history), concomitant medications, underlying liver disease, presence or absence of ascites, presence or absence of hepatic encephalopathy, presence or absence of gastroesophageal varices, date of onset of HCC, history of HCC treatment, and disease stage.

## (2) Observation and examination items

**<Screening period>**

Hematology (WBC, Hgb, Plt), blood biochemistry (T-Bil, TP, Alb, AST, ALT, ALP, GGT, BUN, Cre, T-cho, HbA1c, NH3), coagulation tests (PT, PT-INR), tumor markers (AFP, AFP-L3, PIVKA-II), urinalysis (urinary protein, beta2-microglobulin, NAG), and imaging examinations (number of tumors, tumor diameter, including the head) (CT and/or MRI).

**<Intervention period>**

Treatment-related information (drug dose, treatment start and end dates, date of confirmed progression, reason for treatment discontinuation, grade >=3 adverse events, whether dose reduction/discontinuation occurred and the reason, and antitumor effect).

Hematology (WBC, Hgb, Plt), blood biochemistry (T-Bil, TP, Alb, AST, ALT, ALP, GGT, BUN, Cre, T-cho, HbA1c, NH3), coagulation tests (PT, PT-INR), tumor markers (AFP, AFP-L3, PIVKA-II), urinalysis (urinary protein, beta2-microglobulin, NAG), and imaging examinations (CT and/or MRI).

**<Observation period>**

Treatment-related information (whether additional treatment was given).

Hematology (WBC, Hgb, Plt), blood biochemistry (T-Bil, TP, Alb, AST, ALT, ALP, GGT, BUN, Cre, T-cho, HbA1c, NH3), coagulation tests (PT, PT-INR), tumor markers (AFP, AFP-L3, PIVKA-II), and imaging examinations (CT and/or MRI).

## (3) Confirmation of subjective and objective symptoms

This will be confirmed by interview.

## (4) Confirmation of adverse events (*) and adverse drug reactions

The contents of adverse events, onset and resolution times, severity, treatment, outcome, seriousness assessment, causal relationship to the study drug, and related information will be recorded in the case report form (CRF). Follow-up investigations will be performed as necessary.

(*) Any unfavorable or unintended sign, symptom, or disease occurring in a study participant during the study period.

## (5) Efficacy assessment

During protocol treatment, from treatment initiation up to 12 months, contrast-enhanced abdominal CT or MRI and imaging examinations of sites with target lesions will be performed every 12 weeks (+/-2 weeks), and tumor response will be assessed according to RECIST v1.1 (*) and mRECIST (**). As far as possible, the same imaging modality as at baseline should be used for assessment.

(*) New Response Evaluation Criteria in Solid Tumors (version 1.1): the new guideline for evaluating treatment response in solid tumors.

(**) Modified Response Evaluation Criteria in Solid Tumors: criteria that add decreased blood flow due to tumor necrosis to the RECIST-based assessment of tumor shrinkage.

Participants will undergo this efficacy assessment until a discontinuation criterion is met.

For non-target lesions outside the CT scan range from chest to pelvis, such as bone or brain lesions, head MRI or head CT (contrast-enhanced MRI is preferable), bone scintigraphy, or PET may be performed as appropriate when symptoms appear or a new lesion is suspected; mandatory 12-week examinations are not required for these lesions.

*[The schedule table has been omitted in this English text-only version.]*

An open circle indicates an item performed before obtaining consent, and a filled circle indicates an item performed after obtaining consent.

a) During study treatment, imaging examinations will be performed every 12 weeks; however, additional imaging may be performed as needed depending on the participant's condition.

b) Hematology, blood biochemistry, coagulation tests, tumor marker tests, urinalysis, and imaging examinations.

# 6. Evaluation Endpoints

## (1) Primary endpoint

Response rate (***) according to mRECIST.

Rationale: This is an appropriate indicator for evaluating the efficacy of cisplatin combination therapy in this trial.

(***) Sum of complete response and partial response.

## (2) Secondary endpoints

1. Response rate according to RECIST version 1.1.

2. Progression-free survival rate.

3. Disease control rate.

4. Overall survival.

5. Response rate, progression-free survival, disease control rate, and overall survival according to prior treatment.

6. Conversion rate to curative treatment.

7. Adverse events.

Rationale: Endpoints 1-6 are included to compare the efficacy of cisplatin combination therapy in this trial with results from other studies. Endpoint 7 is included to ensure the safety of study participants.

# 7. Target Sample Size and Rationale

At our institution, 40 cases are planned.

For the study as a whole, 11 participating institutions and a target sample size of 100 cases are planned.

Rationale:

The primary endpoint of this trial is the response rate. Based on previous clinical trial results showing a response rate of 27.3% with atezolizumab plus bevacizumab and approximately 30% with cisplatin preparations, the threshold response rate is set at 27% and the expected response rate at 52%. Assuming a 1:1 allocation and a one-sided alpha error of 0.05, 47 patients per arm are required to achieve 80% statistical power. Allowing for approximately 5% exclusions and dropouts, the target sample size was set at 100.

# 8. Data Aggregation and Statistical Analysis

## (1) Analysis set

The analysis set will be the Full Analysis Set (FAS), defined as all randomized cases in which study treatment has been started.

## (2) Analytical methods

The obtained data will be entered into a dedicated database and aggregated and analyzed. The response rate, blood test results, presence or absence of adverse events, and correlations with participant background factors will be analyzed using the chi-square test. Progression-free survival and overall survival will be analyzed using the Kaplan-Meier method with the log-rank test. Confidence intervals will be 95%.

# 9. Study Period

From the date of publication in the jRCT after approval by the Certified Clinical Research Review Board until December 31, 2025.

(Enrollment deadline: December 31, 2023.)

# 10. Policy for Selecting Study Participants

## (1) Inclusion criteria

Participants must meet all of the following criteria:

1. Age >=20 years at the time of consent.

2. Male or female.

3. PS 0 or 1.

4. Diagnosed with unresectable hepatocellular carcinoma.

5. No prior treatment with hepatic arterial infusion chemotherapy using cisplatin.

6. Scheduled to start atezolizumab plus bevacizumab.

7. Has measurable intrahepatic lesions.

8. Has received sufficient explanation regarding participation in this study, fully understands the study, and has provided written informed consent voluntarily.

## (2) Exclusion criteria

Participants meeting any of the following criteria will not be enrolled in this study:

1. Child-Pugh score >=8.

2. History of TACE on two or more occasions (except combination therapy performed for curative intent).

3. Allergy to CT contrast agent.

4. Estimated glomerular filtration rate (eGFR) <30.

5. History of hypersensitivity to any component of cisplatin.

6. History of hypersensitivity to any component of atezolizumab.

7. History of hypersensitivity to any component of bevacizumab.

8. History of hemoptysis.

9. Complicated by autoimmune disease or with a history of chronic or recurrent autoimmune disease.

10. Complicated by interstitial pneumonia.

11. Intra-abdominal inflammation or unhealed surgical wounds.

12. Brain metastases.

13. Congenital bleeding tendency or coagulation abnormality.

14. Serious heart disease such as congestive heart failure or coronary artery disease.

15. Pregnant women or women who may be pregnant.

16. Any other patient judged by the principal investigator or subinvestigator to be inappropriate as a study participant, for example because introduction of chemotherapy is considered difficult.

# 11. Background of the Study and Scientific Rationale (Rationality and Validity)

Previous studies have shown the individual therapeutic effects of cisplatin HAIC and atezolizumab plus bevacizumab in patients with HCC. If comparison and analysis of response rates, adverse events, and related outcomes between the combination group and the non-combination group clarify the efficacy and safety of this treatment strategy, it may become available in the future as a new treatment option for patients with unresectable HCC.

Both treatment modalities to be combined are already widely used in routine practice at all participating institutions, and therefore no problems are anticipated in implementing the study. In addition, the number of patients with unresectable HCC who initiated systemic chemotherapy at all participating institutions has exceeded 800 over 10 years; therefore, from the viewpoint of case accrual, the target is considered achievable within the study period.

# 12. Procedures for Obtaining Informed Consent

The principal investigator or a subinvestigator will provide the approved written explanation document to the study participant, give sufficient written and oral explanations, and obtain voluntary written informed consent. When information on efficacy, safety, or other matters that could affect the participant's willingness to continue consent becomes available, or when changes to the protocol or related documents that could affect consent are made, the participant will be informed promptly. The participant's intention regarding continued study participation will be confirmed in advance, and after prior approval by the Certified Clinical Research Review Board, the explanatory document, consent form, and other relevant documents will be revised and re-consent will be obtained from the participant.

The written explanation document shall include the following items:

1. The name of the specified clinical research, the fact that the study has been approved by the administrator of the implementing medical institution, and the fact that the implementation plan has been submitted to the Minister of Health, Labour and Welfare.

2. The name of the implementing medical institution and the name and title of the principal investigator.

3. The reason the individual was selected as a participant in the specified clinical research.

4. The expected benefits and disadvantages of the specified clinical research.

5. That participation in the specified clinical research is voluntary and refusal is permitted.

6. Matters concerning withdrawal of consent.

7. That refusal to participate in the specified clinical research or withdrawal of consent will not result in any disadvantageous treatment.

8. How information on the specified clinical research will be disclosed.

9. That the participant or legally acceptable representative may obtain or review the protocol and other materials related to conduct of the specified clinical research, and how such access may be obtained.

10. Matters relating to protection of the participant's personal information.

11. Methods for storage and disposal of specimens and other materials.

12. Whether pharmaceutical manufacturers or other relevant companies are involved in the specified clinical research, and if so, the details of such involvement.

13. The framework for responding to complaints and inquiries.

14. Matters relating to the costs associated with conduct of the specified clinical research.

15. The existence and content of alternative treatments, and a comparison of the expected benefits and disadvantages of those treatments.

16. Matters relating to compensation and medical care for health damage caused by participation in the specified clinical research.

17. Matters reviewed by the Certified Clinical Research Review Board that conducts the review of the specified clinical research, and other matters relating to that board.

18. Any other matters necessary for conduct of the specified clinical research.

# 13. Handling of Personal Information, etc. (including anonymization methods)

All investigators involved in this study will conduct the research in compliance with the Declaration of Helsinki and the Clinical Trials Act. When handling information related to study conduct, each participant will be assigned a study-specific research ID, and a correspondence table linking names to research IDs will be created. Names will be removed from the source data used for the research. Throughout the study period, the correspondence table file will be password-protected and stored securely to prevent leakage. When the results are published, information that could directly identify the participant, such as name or date of birth, will not be included. Information obtained in the study will not be used for purposes other than those of the study.

# 14. Burdens on Participants, Foreseeable Risks and Benefits, Overall Assessment, and Measures to Minimize Burdens and Risks

## (1) Expected benefits

Participants who receive the cisplatin combination treatment in this study may experience improved therapeutic efficacy.

## (2) Expected disadvantages

Among study participants, those who receive the cisplatin combination treatment will need to undergo one day of HAIC using a cisplatin preparation before the planned atezolizumab plus bevacizumab treatment. To receive this treatment, an additional hospitalization period of approximately 5-10 days will be required beyond that needed for atezolizumab plus bevacizumab therapy. There is also the possibility of adverse events due to combination use of HAIC with cisplatin. According to the package insert for the cisplatin preparation (IA-call for arterial infusion), the main adverse events are anorexia (79.8%), nausea/vomiting (76.0%), fever (63.5%), malaise (26.9%), leukopenia (78.8%), neutropenia (77.2%), thrombocytopenia (76.9%), increased AST (GOT) (56.7%), decreased hemoglobin (51.0%), increased ALT (GPT) (45.2%), increased serum bilirubin (36.5%), decreased serum albumin (31.7%), decreased total serum protein (29.8%), increased LDH (27.9%), increased BUN (25.0%), and increased blood creatinine (21.2%), among others.

## (3) Response to adverse events occurring in participants

When an adverse event is observed, the principal investigator and subinvestigators shall immediately take appropriate measures and record the event in the medical record and the case report form. When study drug administration is discontinued or treatment for an adverse event becomes necessary, the participant shall be informed accordingly.

## (4) Amendments to the protocol, etc.

Information necessary for safe conduct of the clinical research will be collected and reviewed. When new safety information or similar findings become available, the protocol and the written explanation/consent documents will be amended as necessary. Any change or revision of the protocol or consent documents requires prior approval by the Certified Clinical Research Review Board. When changes to the implementation plan occur, the following documents shall be submitted to the Minister of Health, Labour and Welfare:

1. Notification of change to implementation plan items (Ministerial Ordinance Form No. 2).

2. Revised implementation plan.

Changes concerning the progress of the specified clinical research shall be made without delay after the change. When the implementation plan is changed, an application for amendment shall promptly be submitted to the Certified Clinical Research Review Board. After approval by the board, the following documents shall be submitted to the Minister of Health, Labour and Welfare:

1. Notification of change to implementation plan items (Ministerial Ordinance Form No. 2).

2. Revised implementation plan.

When the following minor changes are made to the implementation plan, the change details shall be notified to the Certified Clinical Research Review Board within 10 days after the change, and a notification (Ministerial Ordinance Form No. 3) shall also be submitted to the Minister of Health, Labour and Welfare:

1. A change in the name of a person engaged in the specified clinical research that does not involve a change in the personnel engaged in the specified clinical research.

2. A change due to renaming of an area or change in lot number.

## (5) Criteria for discontinuation in individual participants

*[Response when research is discontinued]*

When the principal investigator or subinvestigator judges that continuation of the study is impossible for an individual participant for any of the reasons listed below, the study for that participant shall be discontinued. The reason for discontinuation will be explained to the participant as necessary. After discontinuation, treatment of the participant will be handled sincerely so that the participant does not suffer disadvantage.

*[Discontinuation criteria]*

1. The participant requests to withdraw from study participation or withdraws consent.

2. The entire study is discontinued.

3. The participant meets the treatment discontinuation criteria for any of the study drugs.

4. It is judged difficult to continue the study because of worsening comorbidities or similar reasons.

5. For any other reason, the principal investigator or subinvestigator judges discontinuation of the study to be appropriate.

*[Response when study treatment is discontinued]*

When the principal investigator or subinvestigator judges, for any of the reasons above, that study treatment cannot be continued for an individual participant, the study treatment for that participant shall be discontinued. The reason for discontinuation will be explained to the participant as necessary. After discontinuation, treatment of the participant will be handled sincerely so that the participant does not suffer disadvantage.

Among cases in which study treatment is discontinued, those who have received the study drug will continue to be observed for safety evaluation.

# 15. Methods for Storage and Disposal of Information (including storage period)

The principal investigator shall prepare records concerning the following items:

1. Items identifying study participants.

2. Items concerning medical care and examinations for study participants.

3. Items concerning participation in this study.

4. Other items necessary for conduct of this study.

Specimens and information collected in this study will be stored in a lockable location until five years have elapsed after study completion (at the coordinating study site, the Department of Gastroenterology, Okayama University Hospital; at participating sites, locations designated by each institution), and thereafter disposed of with sufficient attention to personal information protection. Information will be stored after removing direct identifiers such as name, address, and date of birth.

The principal investigator will retain essential documents related to conduct of the research (the protocol, implementation plan, explanatory documents and consent-related documents for participants, the summary report and other documents prepared by the principal investigator under ministerial ordinances or copies thereof, documents concerning review opinions received from the Certified Clinical Research Review Board, monitoring-related documents, the participant identification code list, consent forms, copies of case report forms, and other documents or records necessary to assure data reliability) in a lockable location until five years have elapsed after study completion (Department of Gastroenterology, Okayama University Hospital at the coordinating site; locations designated by each participating site at other institutions), and will thereafter dispose of them with sufficient attention to personal information. Electronic data will be completely deleted, and paper records will be shredded and discarded.

# 16. Access to Source Documents, etc.

In this study, source documents mean the following:

1. Records relating to investigators' consent procedures and provision of information.

2. Medical records, clinical laboratory data, imaging films, and other data at the time of case registration, as well as the records on which case report forms are based.

The principal investigator and the implementing medical institutions will make all clinical research-related records, including source documents, available for direct access during monitoring, audits, and investigations by the Certified Clinical Research Review Board and regulatory authorities.

# 17. Periodic Reporting

The representative investigator will report the following matters concerning the status of this study to the administrator of the implementing medical institution and then to the Certified Clinical Research Review Board once every year, within two months after the end of the relevant period, counting from the date on which information on the study was made public in the jRCT. Unified Form 5 Periodic Report and Appendix Form 3 Periodic Report shall be used.

1. Number of participants enrolled in this study.

2. Occurrence and subsequent course of diseases, etc. related to this study.

3. Occurrence of non-compliance with ministerial ordinances or the protocol related to this study, and subsequent responses.

4. Evaluation of the safety and scientific validity of this study.

5. Matters relating to involvement of pharmaceutical manufacturers and related entities in this study.

In addition, within one month from the date on which the Certified Clinical Research Review Board states its opinion, the following information shall be reported to the Minister of Health, Labour and Welfare using Appendix Form 3:

1. The name of the Certified Clinical Research Review Board stated in the implementation plan.

2. Whether the board judged continuation of this study to be appropriate.

3. Number of participants enrolled in this study.

The representative investigator will provide information to each site principal investigator regarding the contents of the periodic report and approval by the board. Each principal investigator will also report the above periodic report and related documents to the administrator of his or her own implementing medical institution.

# 18. Source of Funding, Conflicts of Interest Related to the Study During the Study Period, Personal Financial Interests, and Status of Conflicts of Interest Related to the Research

This study will be conducted using research funding from Nippon Kayaku Co., Ltd. The principal investigators will declare conflicts of interest and obtain review and approval in accordance with the 'Guidance on Conflict of Interest Management under the Clinical Trials Act.' At the time of periodic reporting, it will be confirmed whether there have been any changes in institutional or personal conflicts of interest, and such matters will be reported to the Certified Clinical Research Review Board.

# 19. Public Disclosure of Information Related to the Study (registration of the study plan and publication of results)

This study will be registered in the Japan Registry of Clinical Trials (jRCT). The results obtained in this study will be presented through the jRCT and at academic meetings, and are also planned to be published as a paper in a peer-reviewed academic journal.

# 20. Response to Consultations, etc. from Study Participants

The principal investigator and subinvestigators will respond appropriately and promptly to consultations, inquiries, complaints, and related matters from study participants and their related persons.

At Okayama University Hospital, the following consultation desk will be provided.

Person responsible for consultation desk:

Yasuto Takeuchi, Center for Innovative Clinical Medicine, Okayama University Hospital, 2-5-1 Shikata-cho, Kita-ku, Okayama 700-8558, Japan.

Phone: 086-235-7219 (weekdays 9:00-17:00); 086-235-6724 (weekday evenings, weekends, and holidays). Fax: 086-225-5991.

Complaint consultation desk:

Clinical Trial and Clinical Research Consultation Desk, General Patient Support Center, Okayama University Hospital.

Phone: 086-235-7744 (weekdays 8:30-17:00).

E-mail: iscps@okayama-u.ac.jp.

# 21. Procedures When Informed Consent Is Obtained from a Legally Authorized Representative, etc.

No legally authorized representative will be designated in this study.

# 22. Procedures for Obtaining Informed Assent (including explanatory items and methods)

Not applicable to this study.

# 23. Procedures to Confirm That All Requirements for Research Conducted in an Urgent and Obvious Life-Threatening Situation Are Met

Not applicable to this study.

# 24. Financial Burden on Participants or Honoraria, If Any

The medical costs for treatment provided in this study will be covered by the participant's health insurance because the treatments are within the approved indications; however, participants receiving the cisplatin combination treatment may incur additional costs due to prolonged hospitalization or additional inpatient treatment associated with study participation. No honorarium will be provided for participation in this study.

# 25. Measures to Be Taken When Diseases, etc. Occur

## (1) Definition of diseases, etc.

'Diseases, etc.' means diseases, disorders, death, or infections suspected to have been caused by conduct of the specified clinical research.

## (2) Serious diseases, etc.

The seriousness of diseases, etc. suspected to have been caused by conduct of the specified clinical research is defined as follows:

1) Death or conditions that may lead to death.

2) The following items (1)-(5) other than those in item 1):

(1) Conditions requiring hospitalization or prolongation of hospitalization for treatment.

(2) Disability.

(3) Disease that may lead to disability.

(4) Conditions that are serious in the same way as items (1)-(3), death, or conditions that may lead to death.

(5) Congenital disease or abnormality in subsequent generations.

## (3) Causal relationship with the study

The representative investigator, principal investigator, or subinvestigator will determine the relationship of all diseases, disorders, deaths, and infections to study conduct or study treatment. The judgment will take into account not only the temporal relationship with initiation of study treatment, but also the course of the underlying disease, comorbidities, concomitant medications, study procedures, accidents, and other external factors.

## (4) Expectedness

The expectedness of diseases will be judged based on the package insert and interview form. If the nature, severity, or frequency of an adverse event does not match the known information, it will be treated as an unknown adverse event. The expected adverse events are as follows.

1. Arterial infusion cisplatin

**<Overview>**

In a domestic clinical trial involving 104 patients with HCC, the incidence of adverse drug reactions and abnormal laboratory values was 99.0%. The main events were anorexia (79.8%), nausea/vomiting (76.0%), fever (63.5%), malaise (26.9%), leukopenia (78.8%), neutropenia (77.2%), thrombocytopenia (76.9%), increased AST (GOT) (56.7%), decreased hemoglobin (51.0%), increased ALT (GPT) (45.2%), increased serum bilirubin (36.5%), and related abnormalities.

**<Serious adverse reactions>**

1) Acute renal failure (frequency unknown).

2) Bone marrow suppression such as thrombocytopenia (23.2%), leukopenia (20.8%), neutropenia (18.7%), anemia (<1%), and pancytopenia (frequency unknown).

3) Thrombocytopenia (1.4%).

4) Shock and anaphylaxis (frequency unknown).

5) Hepatic dysfunction (35.4%), jaundice (<1%), and fulminant hepatitis (frequency unknown).

6) Hepatobiliary disorders (<1%).

7) Myocardial infarction (<1%), angina pectoris (frequency unknown), congestive heart failure (frequency unknown), and arrhythmia (<1%).

8) Pulmonary tuberculosis (<1%).

9) Hearing impairment (<1%).

10) Papilledema, retrobulbar optic neuritis, and cortical blindness (all frequency unknown).

11) Cerebral infarction (frequency unknown).

12) Hemolytic uremic syndrome (frequency unknown).

13) Hemolytic anemia (frequency unknown).

14) Interstitial pneumonia (frequency unknown).

15) Syndrome of inappropriate antidiuretic hormone secretion (frequency unknown).

16) Gastrointestinal bleeding, peptic ulcer, and gastrointestinal perforation (all frequency unknown).

17) Acute pancreatitis (frequency unknown).

18) Hyperglycemia (<1%) and worsening diabetes mellitus (frequency unknown).

19) Rhabdomyolysis (frequency unknown).

2. Atezolizumab

**<International phase III clinical trial (IMbrave150)>**

In 329 patients (including 35 Japanese patients) who received this drug plus bevacizumab, adverse drug reactions were observed in 276 patients (83.9%). The main adverse drug reactions (>=10%) were hypertension in 78 patients (23.7%), proteinuria in 62 (18.8%), fatigue in 50 (15.2%), increased AST in 46 (14.0%), pruritus in 43 (13.1%), and related events.

**<Serious adverse reactions>**

1) Interstitial lung disease (2.7%).

2) Hepatic dysfunction and hepatitis: increased AST (5.5%), increased ALT (5.5%), increased ALP (1.9%), increased gamma-GTP (0.8%), increased bilirubin (1.4%), etc.

3) Colitis (1.2%) and severe diarrhea (1.4%).

4) Pancreatitis (0.1%).

5) Type 1 diabetes mellitus (0.1%).

6) Thyroid dysfunction: hypothyroidism (7.5%), hyperthyroidism (2.9%), thyroiditis (0.4%), etc.

7) Adrenal dysfunction: adrenal insufficiency (0.4%), acute adrenocortical insufficiency (<0.1%), etc.

8) Pituitary dysfunction: hypophysitis (0.3%), hypopituitarism (0.1%), etc.

9) Encephalitis (0.1%) and meningitis (0.1%).

10) Neurologic disorders: peripheral neuropathy (3.1%), polyneuropathy (0.2%), Guillain-Barre syndrome (0.1%), etc.

11) Myasthenia gravis (frequency unknown).

12) Severe skin disorders: erythema multiforme (0.2%), toxic epidermal necrolysis (frequency unknown), Stevens-Johnson syndrome (frequency unknown), etc.

13) Renal dysfunction: acute kidney injury (0.4%), renal failure (0.4%), tubulointerstitial nephritis (0.3%), nephritis (0.2%), etc.

14) Myositis (0.1%) and rhabdomyolysis (0.1%).

15) Myocarditis (0.1%).

16) Hemophagocytic syndrome (<0.1%).

17) Infusion reaction (2.8%).

18) Febrile neutropenia (2.8%).

3. Bevacizumab

**<International phase III clinical trial (YO40245 [IMbrave150])>**

In 329 patients (including 35 Japanese patients) who received this drug plus atezolizumab, adverse drug reactions were observed in 276 patients (83.9%). The main adverse drug reactions (>=10%) were hypertension in 78 patients (23.7%), proteinuria in 62 (18.8%), fatigue in 50 (15.2%), increased AST in 46 (14.0%), pruritus in 43 (13.1%), and related events.

**<Serious adverse reactions>**

1) Shock and anaphylaxis (1.9%).

2) Gastrointestinal perforation (0.9%).

3) Fistula (0.3%).

4) Delayed wound healing, including wound dehiscence (0.5%) and postoperative bleeding (0.4%), because impairment of wound healing may occur.

5) Hemorrhage (19.3%).

6) Thromboembolism: cerebral infarction (0.2%), transient ischemic attack (0.1%), angina pectoris (0.1%), myocardial infarction (<0.1%), cerebrovascular accident (frequency unknown), cerebral ischemia (frequency unknown), etc.

7) Hypertensive encephalopathy (frequency unknown) and hypertensive crisis (frequency unknown).

8) Reversible posterior leukoencephalopathy syndrome (<0.1%).

9) Nephrotic syndrome (<0.1%).

10) Bone marrow suppression, including pancytopenia (<0.1%), neutropenia (24.5%), leukopenia (24.3%), anemia (8.7%), and thrombocytopenia (10.4%) when used in combination with other antineoplastic agents.

11) Infection (10.0%).

12) Congestive heart failure (<0.1%).

13) Interstitial pneumonia (0.4%).

14) Thrombotic microangiopathy (frequency unknown).

15) Arterial dissection (<0.1%).

## (5) Outcome

The principal investigator and related investigators will provide appropriate measures and treatment for all diseases, etc. that occur, and will conduct follow-up until symptoms disappear, laboratory values return to the reference range or pre-treatment values, or until the principal investigator determines that medical follow-up is no longer necessary. Outcomes of adverse events will be classified as follows: 1) recovered (complete disappearance of symptoms, findings, and abnormal laboratory values), etc.

## (6) Responsibilities of the principal (representative) investigator and subinvestigators (reporting of serious diseases, etc.)

1. If the principal (representative) investigator or subinvestigator becomes aware of the occurrence of a serious disease, etc. in conduct of the specified clinical research, he or she must take necessary measures such as explaining the matter to the participant and providing treatment.

2. When a principal investigator becomes aware of the occurrence of a disease, etc., he or she must promptly report it to the administrator of the implementing medical institution and then report it to the representative investigator.

3. When the representative investigator becomes aware of the occurrence of a disease, etc., he or she must report it to the administrator of the implementing medical institution and, in accordance with the ministerial ordinance, report it to the Certified Clinical Research Review Board named in the implementation plan using Unified Form 8.

4. When the representative investigator becomes aware of any matter relating to occurrence of diseases, etc. suspected to have been caused by conduct of the specified clinical research, and the matter falls under items specified by ministerial ordinance (see table in the original protocol), he or she must report it to the Minister of Health, Labour and Welfare using Appendix Form 2-1 (drugs) in accordance with the ministerial ordinance.

*[The reporting table referred to above has been omitted in this English text-only version.]*

# 26. Compensation for Health Damage

This study uses approved drugs within their approved scope; therefore, no clinical research insurance will be purchased. If health damage occurs as a result of participation in this study, it will be addressed using the participant's health insurance.

# 27. Reporting of Non-Compliance

If a principal investigator becomes aware that the clinical research is in a state of non-compliance with laws/regulations or the protocol ('non-compliance'), he or she must promptly report it to the administrator of the implementing medical institution and to the representative investigator. If a subinvestigator becomes aware of non-compliance, he or she must promptly report it to the principal investigator. When the representative investigator becomes aware of non-compliance, he or she must take the necessary measures in accordance with applicable requirements.

'Serious non-compliance' means non-compliance that affects the human rights or safety of study participants or the reliability of study progress and results. Examples include failure to comply with the inclusion/exclusion criteria, discontinuation criteria, or prohibited concomitant therapies. However, deviations from the protocol for unavoidable medical reasons, such as avoidance of urgent danger to a study participant, are not included.

# 28. Completion or Early Termination of the Study

## (1) Completion of the study

When the period for collecting data related to the primary endpoint described in the protocol has ended, the representative investigator will, in principle, prepare a primary endpoint report within one year. When the period for collecting data related to all endpoints described as study content has ended, the representative investigator will, in principle, prepare a summary report and its synopsis within one year and submit them to the relevant parties as required.

When the time for preparing the primary endpoint report and the summary report coincides, preparation of the summary report shall be deemed to satisfy preparation of the primary endpoint report.

The representative investigator shall submit the synopsis of the primary endpoint report or summary report to the Minister of Health, Labour and Welfare within one month from the date on which the Certified Clinical Research Review Board states its opinion, and shall make it public in the jRCT. When submitting the synopsis of the summary report to the Minister, the protocol and the explanatory document shall also be submitted.

When the representative investigator prepares the primary endpoint report or summary report and its synopsis, he or she shall submit them to the head of the implementing medical institution without delay. When public disclosure has been made, the representative investigator shall promptly report this to the administrator of the implementing medical institution. When the synopsis of the primary endpoint report or summary report has been publicly disclosed, the representative investigator shall provide information to the other principal investigators.

## (2) Early termination of the study

The representative investigator shall consider whether study conduct can continue if any of the following applies:

1. Important information is obtained regarding the quality, safety, or efficacy of the medicinal products used in this study.

2. Recruitment of study participants is difficult and achievement of the planned sample size is judged to be extremely unlikely.

3. The study objective has been achieved before reaching the planned sample size or planned study period.

4. The Certified Clinical Research Review Board instructs changes to the implementation plan or related matters and it is judged difficult to accept them.

If the Certified Clinical Research Review Board recommends or instructs study termination, the study shall be terminated.

When termination of the study is decided, notification shall be given within 10 days from the date of termination to the Certified Clinical Research Review Board named in the implementation plan using Unified Form 11, and a notification shall also be submitted to the Minister of Health, Labour and Welfare using Form No. 4. Even after submitting the termination notification, the representative investigator shall continue necessary responses concerning diseases, etc. until the clinical research is fully completed.

# 29. Handling of Study Results (including incidental findings) When Important Findings Concerning Participants' Health or Genetic Characteristics May Be Obtained

If important findings related to the health of study participants or other important findings are obtained in association with conduct of the study, the participant may be informed of such findings.

# 30. Outsourcing of Part of Study-Related Work

No part of the work related to this study will be outsourced to another institution.

# 31. Possibility of Future Use of Specimens/Information Obtained in This Study

There is a possibility that information obtained in this study may be used for future research; if so, approval from the ethics committee will be obtained.

# 32. Monitoring and Audit System and Procedures

## (1) Monitoring

Monitoring will be conducted in this study. Specific procedures will be described separately in a monitoring plan.

## (2) Audit

No audit will be conducted in this study.

# 33. Ownership of Intellectual Property Rights

No intellectual property rights or similar rights are expected to arise from this study.

# 34. References

1) National Cancer Center, Center for Cancer Control and Information Services. Cancer mortality data based on vital statistics. 2018 (URL in original protocol).

2) The Japan Society of Hepatology, ed. Clinical Practice Guidelines for Hepatocellular Carcinoma, revised 2017 edition. Kanehara, 2020.

3) Kondo M, Morimoto M, Kobayashi S, Ohkawa S, Hidaka H, Nakazawa T, et al. Randomized, phase II trial of sequential hepatic arterial infusion chemotherapy with cisplatin followed by sorafenib versus sorafenib alone in patients with advanced hepatocellular carcinoma (SCOOP-2 trial).

4) Ikeda M, Shimizu S, Sato T, Morimoto M, Kojima Y, Inaba Y, et al. Sorafenib plus hepatic arterial infusion chemotherapy with cisplatin versus sorafenib for advanced hepatocellular carcinoma: randomized phase II trial.

5) Finn RS, et al. Atezolizumab plus Bevacizumab in Unresectable Hepatocellular Carcinoma. N Engl J Med. 2020;382(20):1894-1905.

6) Sandler A, et al. Paclitaxel-Carboplatin Alone or with Bevacizumab for Non-Small-Cell Lung Cancer. N Engl J Med. 2006;355:2542-2550.

7) Tewari KS, et al. Bevacizumab for advanced cervical cancer: final overall survival and adverse event analysis of a randomised, controlled trial.

8) Botrel TEA, et al. Efficacy of bevacizumab plus chemotherapy compared to chemotherapy alone in previously untreated locally advanced or metastatic non-small-cell lung cancer: meta-analysis.
