# Supplementary material for: Atezolizumab–bevacizumab with or without cisplatin-based HAIC for unresectable HCC: A randomized phase II trial
Source: JHEP Rep. 2026 Aug 29;8(11):102002. doi: 10.1016/j.jhepr.2026.102002 (PMC13659464; doi:10.1016/j.jhepr.2026.102002)
Supplement: Supplementary file 5 [file mmc4.zip › Clinical Trial/English_Protocol_ver2.0.docx]

Study Protocol

*Evaluation of the Safety and Efficacy of Combination Therapy with Hepatic Arterial Infusion Chemotherapy and Systemic Chemotherapy (Atezolizumab plus Bevacizumab) for Unresectable Hepatocellular Carcinoma*

## Principal Investigator

Yasuto Takeuchi, MD

Center for Innovative Clinical Medicine, Okayama University Hospital

2-5-1 Shikata-cho, Kita-ku, Okayama 700-8558, Japan

Tel: 086-235-7219

Fax: 086-225-5991

## Study Office

Center for Innovative Clinical Medicine, Okayama University Hospital

Study Office Contact: Yasuto Takeuchi

2-5-1 Shikata-cho, Kita-ku, Okayama 700-8558, Japan

Tel: 086-235-7219 (weekdays 9:00–17:00)

Tel: 086-235-6724 (weekday nights and holidays)

Fax: 086-225-5991

## Planned Study Period

From the date of publication on jRCT after approval by the Certified Clinical Research Review Board until December 31, 2026.

Version 1.0 created on July 20, 2021

Version 2.0 created on September 25, 2023

# 1. Study Title

Evaluation of the Safety and Efficacy of Combination Therapy with Hepatic Arterial Infusion Chemotherapy and Systemic Chemotherapy (Atezolizumab plus Bevacizumab) for Unresectable Hepatocellular Carcinoma

# 2. Study Organization (Including Names of Study Institutions and Investigators)

This study will be conducted under the following structure.

## Principal Investigator

Affiliation: Center for Innovative Clinical Medicine, Okayama University Hospital

Position: Physician

Name: Yasuto Takeuchi

2-5-1 Shikata-cho, Kita-ku, Okayama 700-8558, Japan

Tel: 086-235-7219

## Contact Information

Study Office, Center for Innovative Clinical Medicine, Okayama University Hospital

Contact person: Yasuto Takeuchi

Address: 2-5-1 Shikata-cho, Kita-ku, Okayama 700-8558, Japan

Tel: 086-235-7219 (weekdays 9:00–17:00)

Tel: 086-235-6724 (weekday nights and holidays)

Fax: 086-225-5991

## Monitoring Supervisor

Affiliation: Department of General Medicine, Southwestern Okayama (Kasaoka), Okayama University Hospital

Position: Associate Professor

Name: Shigeru Horiguchi

## Participating Institutions

See separate appendix. (Table omitted in this English text-only version.)

# 3. Background, Objective, and Significance of the Study

In Japan, the incidence of primary liver cancer is high by global standards, and deaths due to hepatocellular carcinoma (HCC) rank fifth among all malignant tumors in this country.1) In principle, treatment for HCC is provided in accordance with the treatment algorithm defined in the Clinical Practice Guidelines for Hepatocellular Carcinoma.2) Under this algorithm, when a patient is judged to have unresectable disease because of vascular invasion or extrahepatic metastasis, systemic chemotherapy with molecular targeted agents or related therapies is recommended for patients with good performance status (PS) and preserved liver function reserve. Currently approved systemic therapies for unresectable HCC in Japan include sorafenib in 2009, regorafenib in 2017, lenvatinib in 2018, ramucirumab in 2019, atezolizumab (an immune checkpoint inhibitor) plus bevacizumab (an angiogenesis inhibitor) in 2020, and cabozantinib.

As described above, multiple agents are now available for unresectable HCC. At the same time, hepatic arterial infusion chemotherapy (HAIC) is also an available option in the same treatment algorithm, and previous reports have shown tumor shrinkage effects with response rates ranging from 0% to 71%, although with considerable variability.2) At present, HAIC mainly consists of cisplatin monotherapy; however, the timing of its introduction is not standardized, and there is no clear evidence regarding its combination with systemic chemotherapy. In this context, a phase II trial comparing sorafenib alone with cisplatin HAIC followed by sorafenib (the SCOOP-2 trial) showed a response rate of 14.3% in the cisplatin-first HAIC group and 9.1% in the sorafenib-alone group; although the difference was not statistically significant, outcomes were more favorable in the cisplatin-first HAIC group.3) Another phase II trial comparing sorafenib alone with sorafenib plus cisplatin HAIC showed a significantly prolonged median survival time (MST) in the combination group, and response rates were 21.7% in the combination group versus 7.3% in the sorafenib-alone group; again, although the difference in response rate was not statistically significant, the combination group showed more favorable results.4)

At present, atezolizumab plus bevacizumab is recommended as systemic chemotherapy. While the response rate of sorafenib was 12%, the response rate of atezolizumab plus bevacizumab was higher at 27%.5) It is therefore expected that combining cisplatin, a cytotoxic anticancer drug, with an immune checkpoint inhibitor and an angiogenesis inhibitor may produce a higher response rate than existing treatment strategies, as has also been reported in lung cancer and cervical cancer.6)7) On the other hand, a meta-analysis has reported an increased incidence of serious adverse events with the combination of cisplatin and angiogenesis inhibitors, including proteinuria, hypertension, bleeding events, neutropenia, febrile neutropenia, and treatment-related death.8) Accordingly, the purpose of this study is to investigate in detail the characteristics of adverse events associated with the combination therapy and to evaluate not only its efficacy but also its safety, thereby contributing to consideration of future recommended treatment strategies.

# 4. Study Methods

## (1) Study Type and Design

Open-label, randomized, two-arm comparative study.

## (2) Study Outline (See Study Flowchart)

Eligibility will be confirmed against the inclusion and exclusion criteria, and written informed consent will be obtained from the study participant. After pre-registration examinations (screening examinations), eligibility will be confirmed again and the participant will be registered. Investigational treatment will then be started according to the allocation result. After the prescribed intervention, the response rate, which is the study outcome, will be compared between the groups.

## (3) Expected Duration of Study Participation for Each Participant (Intervention Period Per Participant)

Intervention period: from the time informed consent is obtained for this study until disease progression or the participant meets the discontinuation criteria.

Expected participation period: approximately 2 years.

(Screening period: approximately 8 weeks; intervention period: induction and maintenance until disease progression or discontinuation criteria are met [average approximately 8 months expected]; observation period: 12 months.)

## (4) Overview of the Medicinal Products/Medical Devices Used and Details of the Intervention

Overview:

Cisplatin formulation

Brand name / Marketing authorization holder: IA-call® for intra-arterial administration 50 mg (Nippon Kayaku Co., Ltd.)

Dosage form / Characteristics / Content: An injectable formulation containing 50 mg cisplatin per vial, dissolved immediately before use. Yellow powdered injection; pH 4.5–7.0 when 100 mg cisplatin is dissolved in 100 mL normal saline.

Pharmacologic class: Antineoplastic agent.

Indication: Hepatocellular carcinoma.

Dosage and administration: Add 70 mL normal saline to each 100 mg of cisplatin to dissolve. Administer 65 mg/m² (body surface area) into the hepatic artery through a catheter inserted into the hepatic artery once daily over 20–40 minutes, followed by a 4–6-week rest period. This constitutes one course, and courses may be repeated. The dose may be reduced as appropriate depending on symptoms and other factors. To reduce nephrotoxicity, the following measures will be taken at the time of administration: (1) administer 1,000–2,000 mL of appropriate infusion fluid over at least 4 hours before administration; (2) administer 1,500–3,000 mL of appropriate infusion fluid over at least 6 hours from the start of administration until completion; and (3) carefully ensure urine output during administration and, if necessary, administer diuretics such as mannitol and furosemide.

Storage conditions: Vial—store at room temperature, protected from light. After dissolution—administer promptly.

Atezolizumab

Brand name / Marketing authorization holder: Tecentriq® for intravenous infusion 1200 mg (Chugai Pharmaceutical Co., Ltd.)

Dosage form / Characteristics / Content: An injectable formulation containing 1200 mg atezolizumab (genetically engineered) per vial (20.0 mL), dissolved immediately before use. Clear to opalescent, colorless to brownish-yellow liquid; pH 5.5–6.1.

Pharmacologic class: Antineoplastic agent / anti-PD-L1 humanized monoclonal antibody.

Indication: Unresectable hepatocellular carcinoma.

Dosage and administration: In combination with bevacizumab (genetically engineered), administer atezolizumab 1200 mg intravenously over 60 minutes every 3 weeks in adults. If the first infusion is well tolerated, the infusion time may be shortened to 30 minutes from the second dose onward.

Storage conditions: Vial—2–8°C. After opening the outer box—protect from light. After preparation—use promptly.

Bevacizumab

Brand name / Marketing authorization holder: Avastin® for intravenous infusion 100 mg/4 mL (Chugai Pharmaceutical Co., Ltd.)

Dosage form / Characteristics / Content: An injectable formulation containing 100 mg bevacizumab (genetically engineered) per vial, dissolved immediately before use. Clear to opalescent, colorless to pale brown liquid; pH 5.9–6.3.

Pharmacologic class: Antineoplastic agent / anti-VEGF humanized monoclonal antibody.

Indication: Unresectable hepatocellular carcinoma.

Dosage and administration: In combination with atezolizumab (genetically engineered), administer bevacizumab 15 mg/kg (body weight) intravenously in adults. The dosing interval shall be at least 3 weeks.

Storage conditions: Vial—2–8°C. After opening the outer box—protect from light. After preparation—use promptly.

Intervention details:

## IA-call Administration Group

After hepatic arterial infusion of IA-call® for intra-arterial administration (65 mg/m²), Tecentriq® 1200 mg IV infusion and Avastin® 15 mg/kg IV infusion will be administered within 2 weeks (up to a maximum of 4 weeks is permitted). Tecentriq® and Avastin® will then be repeated every 3 weeks until discontinuation is judged necessary due to tumor progression, adverse events, or other reasons. A discrepancy within ±5% between the calculated dose and the actually administered dose is permitted.

## Non-IA-call Group

Tecentriq® 1200 mg IV infusion and Avastin® 15 mg/kg IV infusion will be administered. These drugs will be repeated every 3 weeks until discontinuation is judged necessary due to tumor progression, adverse events, or other reasons. A discrepancy within ±5% between the calculated dose and the actually administered dose is permitted.

Dosing schedule: Table omitted in this English text-only version.

Criteria for dose interruption / dose reduction / discontinuation:

a. IA-call® for intra-arterial administration: Dose reduction and temporary interruption will be performed in accordance with the appropriate-use guide for IA-call® as follows: creatinine clearance (mL/min) >60: 100% dose; 30–60: 50% dose; <30: 0% dose.

b. Tecentriq® and Avastin®: Dose reduction and temporary interruption will be performed in accordance with the appropriate-use guides for Tecentriq® and Avastin®. Adverse event grades will be defined according to CTCAE v5.0. If multiple adverse events occur during the same period, the strictest applicable management rule will be followed.

Interstitial pneumonia: For Grade 2, treatment will be interrupted. If the event does not improve to Grade 1 or lower within 12 weeks, treatment will be permanently discontinued. If it recurs, it will be handled as a Grade 4 event. For Grade 3 or 4, treatment will be discontinued.

Hepatic dysfunction / hepatitis: For all of the following events, treatment will be interrupted: baseline AST/ALT within the normal range with on-treatment AST/ALT >3×ULN to ≤10×ULN; baseline AST/ALT >1×ULN to ≤3×ULN with on-treatment AST/ALT >5×ULN to ≤10×ULN; or baseline AST/ALT >3×ULN to ≤5×ULN with on-treatment AST/ALT >8×ULN to ≤10×ULN. If such an event persists for more than 5 days and does not improve to baseline or Grade 1 or lower within 12 weeks, treatment will be discontinued. If AST or ALT increases to >10×ULN or total bilirubin increases to >3×ULN, treatment will be discontinued.

Colitis / severe diarrhea: For Grade 2 or 3, treatment will be interrupted. If the event does not improve to Grade 1 or lower within 12 weeks, treatment will be discontinued. For Grade 4, treatment will be discontinued.

Pancreatitis: For Grade 3–4 increases in amylase and/or lipase, treatment will be interrupted; if the event does not improve to Grade 1 or lower within 12 weeks, treatment will be discontinued; if it recurs, treatment will be discontinued. For immune-related pancreatitis Grade 2–3, treatment will be interrupted and permanently discontinued if it does not improve to Grade 1 or lower within 12 weeks or if it recurs. Grade 4 immune-related pancreatitis requires discontinuation.

Type 1 diabetes mellitus: For Grade 3–4 hyperglycemia, treatment will be interrupted.

Thyroid dysfunction: For symptomatic hypothyroidism, treatment will be interrupted. For symptomatic thyrotoxicosis, treatment will be interrupted; life-threatening immune-related thyrotoxicosis requires discontinuation.

Adrenal dysfunction: For symptomatic adrenal insufficiency Grade 2–4, treatment will be interrupted. If the event does not improve to Grade 1 or lower within 12 weeks or is not stabilized by replacement therapy, treatment will be discontinued.

Pituitary dysfunction: For hypophysitis Grade 2–3, treatment will be interrupted. If the event does not improve to Grade 1 or lower within 12 weeks, treatment will be discontinued. Recurrence of hypophysitis will be managed as a Grade 4 event. Grade 4 hypophysitis requires discontinuation.

Encephalitis: Treatment will be discontinued for any grade.

Neurologic disorders: For immune-related neurologic disorders Grade 2, treatment will be interrupted; if no improvement to Grade 1 or lower occurs within 12 weeks, treatment will be discontinued. Grade 3–4 immune-related neurologic disorders require discontinuation. Guillain–Barré syndrome requires discontinuation regardless of grade.

Myasthenia gravis: Treatment will be discontinued for any grade.

Skin disorders: For Grade 3, treatment will be interrupted; if the event does not improve to Grade 1 or lower within 12 weeks, treatment will be discontinued. Grade 4 requires discontinuation.

Renal dysfunction: For Grade 2, treatment will be interrupted; if the event does not improve to Grade 1 or lower within 12 weeks, treatment will be discontinued. Grade 3–4 requires discontinuation.

Myositis / rhabdomyolysis: For immune-related myositis Grade 2, treatment will be interrupted; if the event does not improve to Grade 1 or lower within 12 weeks, treatment will be discontinued. For Grade 3, treatment will be interrupted; if it does not improve to Grade 1 or lower within 12 weeks, treatment will be discontinued; if it recurs, it will be handled as a Grade 4 event. Grade 4 requires discontinuation.

Myocarditis: For immune-related myocarditis Grade 2, treatment will be interrupted; if the event does not improve to Grade 1 or lower within 12 weeks, treatment will be discontinued. Grade 3–4 immune-related myocarditis requires discontinuation.

Infusion reaction: For Grade 1, stop the infusion immediately. If symptoms recover and 30 minutes have elapsed, restart at half the infusion rate. If tolerated for 30 minutes at the reduced rate, the original rate may be resumed. If symptoms recur, the current administration will be discontinued. For Grade 2, stop the infusion immediately; restart at half the rate after recovery and 30 minutes; if symptoms recur, discontinue the current administration. Grade 3–4 requires discontinuation.

Other events: Grade 3–4 events require discontinuation.

## (5) Management of Investigational Drugs

The cisplatin formulation, atezolizumab, and bevacizumab used in this study are all already approved drugs and will be appropriately stored and managed in accordance with the approved labeling and other regulatory requirements.

## (6) Rules Regarding Concomitant Medications/Therapies

1) Concomitant medications (therapies)

a. Recommended concomitant/supportive therapies: The following concomitant and supportive therapies are recommended, but omission of these measures will not constitute a protocol deviation.

1. Management of febrile neutropenia (FN): For therapeutic use of G-CSF in patients with FN, see section d, Granulocyte Colony-Stimulating Factor.

2. Nausea and vomiting: Antiemetics should be actively administered in accordance with the JSCO Guidelines for Appropriate Use of Antiemetic Drugs. If oral intake is markedly decreased, fluids and electrolytes should be supplemented.

3. Anemia and thrombocytopenia: Transfusion should be provided as appropriate when anemia (hemoglobin ≤8.0 g/dL) or thrombocytopenia (platelet count ≤2.0×10^4/mm^3) is observed.

b. Permitted concomitant/supportive therapies:

1. Bone-modifying agents such as anti-RANKL antibodies and bisphosphonates; medications for comorbidities such as hypertension and diabetes; and symptomatic treatment such as morphine for pain may be used concomitantly.

2. Palliative radiotherapy outside the abdomen, including radiotherapy for bone metastases at risk of fracture, is permitted. Interruption of protocol treatment is not mandatory during palliative radiotherapy.

3. Steroid administration or prophylactic administration for allergic reactions and similar conditions is permitted.

4. Inactivated influenza vaccine is permitted; however, because encephalitis has been reported after vaccination with this vaccine during immunotherapy, the treating physician should carefully consider the clinical necessity.

5. COVID-19 vaccination is permitted; however, the treating physician should carefully consider the clinical necessity.

6. Other supportive care such as gabapentin or Kampo medicines for peripheral neuropathy, including prophylactic use, and acetaminophen, diphenhydramine, or famotidine for infusion reactions, as well as symptomatic treatment for complications and adverse events, is permitted.

c. Non-permitted concomitant/supportive therapies:

1. During protocol treatment, no concomitant anticancer treatment other than the protocol therapy may be administered, including radiotherapy, hormone therapy other than steroids, and immunotherapy other than the protocol treatment.

d. Granulocyte colony-stimulating factor (G-CSF)

1. Primary prophylactic use of G-CSF (*): Because the risk of FN with this treatment is less than 10%, primary prophylactic G-CSF is not mandatory in accordance with the 2013 Appropriate Use Guidelines for G-CSF and the JSMO Guidelines for the Management of Febrile Neutropenia. Primary prophylaxis should not be given solely because a patient is elderly; however, primary prophylaxis is permitted at the treating physician’s discretion when the patient is considered to be at high risk of FN based on risk factors other than age.

2. Secondary prophylactic use of G-CSF (**): If FN occurred in the previous course, secondary prophylactic G-CSF is recommended from the subsequent course onward, although omission will not constitute a protocol deviation. G-CSF will be administered according to the approved dosage and schedule. Start timing: (i) at least 24 hours after completion of chemotherapy administration; or (ii) when a neutrophil count of <1,000/mm^3 is anticipated. Discontinuation timing (except for item (i)): discontinue when the neutrophil count has passed the nadir and reaches ≥5,000/mm^3, or when the count recovers to ≥2,000/mm^3, no symptoms suggestive of infection are present, and patient safety is judged to be secured based on the response to treatment.

3. Therapeutic use of G-CSF: G-CSF will be administered according to the approved dosage and schedule. However, in patients with risk factors for severe disease such as pneumonia or organ dysfunction, the timing and appropriateness of administration should be carefully considered. Start timing: when fever (in principle ≥38.0°C) is observed with neutrophils <1,000/mm^3, or when neutrophils <500/mm^3 are observed. Discontinuation timing: discontinue when the neutrophil count has passed the nadir and reaches ≥5,000/mm^3, or when the count recovers to ≥2,000/mm^3, no symptoms suggestive of infection are present, and patient safety is judged to be secured based on the response to treatment.

* Primary prophylactic use of G-CSF means administration before FN or prolonged neutropenia occurs, for the purpose of preventing these events during anticancer therapy.

** Secondary prophylactic use of G-CSF means administration after FN or prolonged neutropenia has already occurred once, for the purpose of preventing recurrence during anticancer therapy.

2) Rules regarding prohibited concomitant medications (therapies)

1. Prohibited concomitant use: The following drugs and therapies that may affect this study are prohibited throughout the study period.

(1) Other antineoplastic treatments: chemotherapy, molecular targeted therapy, immunotherapy other than the protocol treatment, antibody therapy, radiotherapy (except where permitted, such as for vascular invasion or palliative radiotherapy outside the abdomen), hyperthermia, and surgery (however, transition to curative treatment such as hepatectomy, radiofrequency ablation, or transarterial chemoembolization after tumor shrinkage/reduction is permitted).

(2) Investigational drugs and unapproved drugs.

## (7) Case Registration and Allocation

The principal investigator or subinvestigator will register cases and perform allocation as follows:

1. Patients who meet all inclusion criteria and none of the exclusion criteria will be considered eligible, and written informed consent will be obtained.

2. The principal investigator or subinvestigator will enter the required information into the EDC, and allocation will be performed according to age, sex, liver functional reserve, and disease stage.

3. According to the allocation result, the corresponding investigational treatment (atezolizumab, bevacizumab, with or without cisplatin formulation) will be administered.

4. If consent withdrawal, discontinuation, dropout, or any similar event occurs, it will be promptly reported to the principal investigator.

## (8) Actions After Completion of the Study

After completion of this study, the principal investigator or subinvestigator will provide each study participant with the medical care considered most appropriate, including care based on the results obtained in this study.

## (9) Use of Information from Other Institutions

In this study, information will be collected from the study site itself and from other institutions and will be analyzed.

1. Method of information collection: In this study, data will be collected using an electronic data capture (EDC) system. Data will be entered promptly after completion of each visit. The EDC will not contain directly identifying personal information such as names or dates of birth.

2. Information obtained from study participants: There are no plans to provide such information to other research institutions.

# 5. Observation and Examination Items

## (1) Participant Background

Age, sex, height, weight, performance status (PS), present illness, comorbidities, past medical history, lifestyle history (smoking history and alcohol history), concomitant medications, background liver disease, presence or absence of ascites, presence or absence of encephalopathy, presence or absence of gastroesophageal varices, date of HCC onset, history of HCC treatment, and disease stage.

## (2) Observation and Examination Items

Screening period: Complete blood count (WBC, Hgb, Plt), biochemical tests (T-Bil, TP, Alb, AST, ALT, ALP, GGT, BUN, Cre, T-cho, HbA1c, NH3), coagulation tests (PT, PT-INR), tumor markers (AFP, AFP-L3, PIVKA-II), urinalysis (urinary protein, β2-microglobulin, NAG), and imaging studies (number of tumors, tumor size, including the head) by CT and/or MRI. Information obtained within 8 weeks before informed consent may be used as a substitute.

Intervention period: Treatment-related information (drug dose, treatment start and end dates, date of confirmed progression, reason for treatment discontinuation, Grade 3 or higher adverse events, presence/absence of dose reduction or discontinuation and the reason, and antitumor effect), complete blood count, biochemical tests, coagulation tests, tumor markers, urinalysis, and imaging studies (CT and/or MRI).

Observation period: Treatment-related information (whether additional treatment was given), complete blood count, biochemical tests, coagulation tests, tumor markers, and imaging studies (CT and/or MRI).

## (3) Confirmation of Subjective and Objective Symptoms

These will be confirmed by medical interview.

## (4) Confirmation of Adverse Events (*) and Adverse Drug Reactions

The details of adverse events, time of onset and resolution, severity, treatment, outcome, seriousness, causal relationship to the investigational drug, and other relevant information will be recorded in the case report form (CRF). Follow-up investigations will be conducted as necessary.

* Adverse event means any unfavorable or unintended sign, symptom, or disease that occurs in a study participant during the study period.

## (5) Efficacy Evaluation

During protocol treatment, contrast-enhanced abdominal CT or MRI and imaging of the site where target lesions are present will be performed every 12 weeks (±2 weeks) during the intervention period, and tumor response will be evaluated according to RECIST v1.1 (*) and mRECIST (**). Baseline evaluation should be performed using the same imaging modality whenever possible.

* New Response Evaluation Criteria in Solid Tumors (version 1.1): new guidelines for evaluating treatment response in solid tumors.

** Modified Response Evaluation Criteria in Solid Tumors: criteria that add reduced blood flow due to tumor necrosis to the RECIST criteria based on tumor shrinkage.

Participants will undergo this efficacy evaluation until they meet the discontinuation criteria.

For non-target lesions not included in the imaging range from the chest to pelvis CT, such as bone or brain lesions, head MRI or head CT (contrast-enhanced MRI is preferable), bone scintigraphy, or PET may be performed as appropriate when symptoms appear or when new lesions are suspected, and routine 12-week testing is not mandatory.

Schedule table: omitted in this English text-only version.

# 6. Endpoints

## (1) Primary Endpoint

Response rate (***) according to mRECIST.

Rationale: This is an appropriate indicator for evaluating the efficacy of adding the cisplatin formulation in this study.

*** Sum of complete response and partial response.

## (2) Secondary Endpoints

1. Response rate according to RECIST version 1.1

2. Progression-free survival

3. Disease control rate

4. Overall survival

5. Response rate, progression-free survival, disease control rate, and overall survival according to prior treatment

6. Rate of transition to curative treatment

7. Adverse events

Rationale: Items 1–6 are included to compare the efficacy of combination treatment with the cisplatin formulation in this trial with results from other studies. Item 7 is included to ensure participant safety.

# 7. Target Sample Size and Rationale

A total of 30 patients are planned at our institution.

For the study as a whole, 13 participating institutions and a target sample size of 67 patients are planned.

Rationale: The primary endpoint of this study is response rate. Based on previous clinical trial results showing a response rate of approximately 25% for atezolizumab plus bevacizumab and approximately 30% for the cisplatin formulation, the threshold response rate was set at 25% and the expected response rate at 50%. Assuming a 1:1 allocation and one-sided alpha error of 0.05, 32 patients in the cisplatin group and 32 patients in the non-cisplatin group are required to detect a difference with 80% statistical power. Allowing for approximately 5% exclusions or dropouts, the target sample size was set at 67 patients.

# 8. Data Aggregation and Statistical Analysis

## (1) Analysis Set

The analysis population will be the Full Analysis Set (FAS), defined as all randomized cases in whom investigational treatment was started.

## (2) Analysis Methods

The data obtained will be entered into a dedicated database and tabulated and analyzed. The response rate, blood test results, presence or absence of adverse events, and correlations with participant background factors will be analyzed using the chi-square test. Progression-free survival and overall survival will be analyzed by the Kaplan–Meier method with the log-rank test. The confidence interval will be 95%.

# 9. Study Period

From the date of publication on jRCT after approval by the Certified Clinical Research Review Board until December 31, 2026.

(Enrollment deadline: December 31, 2024)

# 10. Policy for Selection of Study Participants

## (1) Inclusion Criteria

Participants must meet all of the following criteria:

1. Age 20 years or older at the time of consent.

2. Male or female.

3. PS 0 or 1.

4. Diagnosed with unresectable hepatocellular carcinoma.

5. No prior treatment with hepatic arterial infusion chemotherapy using cisplatin.

6. Scheduled to start atezolizumab plus bevacizumab.

7. Have measurable intrahepatic lesions.

8. Have received sufficient explanation about participation in this study, have adequately understood the study, and have provided written informed consent of their own free will.

## (2) Exclusion Criteria

Participants meeting any of the following criteria will not be enrolled:

1. Child–Pugh score of 8 or higher.

2. Allergy to CT contrast media.

3. Estimated glomerular filtration rate (eGFR) <30.

4. History of hypersensitivity to any component of cisplatin.

5. History of hypersensitivity to any component of atezolizumab.

6. History of hypersensitivity to any component of bevacizumab.

7. History of hemoptysis.

8. Presence of an autoimmune disease or a history of chronic or recurrent autoimmune disease.

9. Concomitant interstitial pneumonia.

10. Intra-abdominal inflammation or unhealed surgical wounds.

11. Brain metastases.

12. Congenital bleeding tendency or coagulation abnormality.

13. Severe heart disease such as congestive heart failure or coronary artery disease.

14. Pregnant women or women who may be pregnant.

15. Any other patient judged by the principal investigator or subinvestigator to be inappropriate for the study, for example because introduction of chemotherapy is difficult.

# 11. Background of the Study and Scientific Rationale (Rationality and Validity)

Previous studies have shown the individual therapeutic effects of cisplatin hepatic arterial infusion chemotherapy and atezolizumab plus bevacizumab in patients with hepatocellular carcinoma. By comparing and analyzing the response rate, adverse events, and other outcomes between the combination group and the non-combination group, if the efficacy and safety of this treatment are clarified, it may become possible in the future to use this regimen as one of the new treatment options for patients with unresectable HCC.

Both treatments to be combined have been used extensively as part of routine clinical practice at all participating institutions, and therefore no major difficulty is anticipated in carrying out the study. In addition, more than 800 patients with unresectable HCC who started systemic chemotherapy have been treated across all participating institutions over 10 years; thus, from the standpoint of case accrual, achievement of the target sample size within the study period is considered feasible.

# 12. Procedures for Obtaining Informed Consent

The principal investigator or subinvestigator will provide the study participant with the explanatory document approved by the Certified Clinical Research Review Board, give a full written and oral explanation, and obtain written voluntary consent. When information on efficacy, safety, or other matters that may affect the participant’s willingness to consent becomes available, or when changes are made to the implementation plan or other aspects that may affect consent, such information will be promptly provided to the participant, and the participant’s intention regarding continued participation will be confirmed in advance. The explanatory document, consent form, and related materials will be revised after prior approval by the Certified Clinical Research Review Board, and re-consent will be obtained from the participant.

The informed consent explanatory document shall include the following:

1. The name of the specified clinical research to be conducted; the fact that approval for implementation has been obtained from the administrator of the medical institution; and the fact that the study plan has been submitted to the Minister of Health, Labour and Welfare.

2. The name of the implementing medical institution and the name and title of the principal investigator.

3. The reason why the individual was selected as a potential participant in the specified clinical research.

4. The expected benefits and disadvantages associated with implementation of the specified clinical research.

5. That refusal to participate in the specified clinical research is voluntary.

6. Matters concerning withdrawal of consent.

7. That refusal to participate or withdrawal of consent will not result in any disadvantageous treatment.

8. The method of public disclosure of information concerning the specified clinical research.

9. That the participant or legally acceptable representative may obtain or review the study protocol and other materials related to conduct of the study, and how such materials may be obtained or reviewed.

10. Matters concerning protection of the participant’s personal information.

11. The methods of storage and disposal of specimens and other materials.

12. Whether and how pharmaceutical manufacturers or other parties are involved in the specified clinical research.

13. The system for responding to complaints and inquiries.

14. Matters concerning costs related to conduct of the specified clinical research.

15. The availability and content of other treatment options and a comparison of their expected benefits and disadvantages.

16. Matters concerning compensation and provision of medical care for health damage caused by implementation of the specified clinical research.

17. Matters reviewed by the Certified Clinical Research Review Board conducting the review and other matters concerning that board.

18. Any other matters necessary for implementation of the specified clinical research.

# 13. Handling of Personal Information (Including Method of Anonymization)

All researchers involved in this study will conduct the study in compliance with the Declaration of Helsinki and the Clinical Trials Act. When handling information related to implementation of the study, a study-specific research ID will be assigned to each participant, and a correspondence table linking the participant’s name and research ID will be created. Names will be removed from the source data before use in the research. Throughout the study period, the correspondence table file will be password protected and strictly managed to prevent leakage. When study results are published, information that can immediately identify individual participants, such as names and dates of birth, will not be included. Information obtained in this study will not be used for purposes other than the objectives of the study.

# 14. Burdens on Study Participants, Foreseeable Risks and Benefits, Overall Assessment, and Measures to Minimize Burdens and Risks

## (1) Expected Benefits

For participants who receive the combination treatment with the cisplatin formulation, therapeutic efficacy may improve.

## (2) Expected Disadvantages

Participants who receive combination treatment with the cisplatin formulation will need to undergo hepatic arterial infusion chemotherapy using cisplatin for 1 day before the planned treatment with atezolizumab plus bevacizumab. To receive this treatment, an additional hospitalization period of approximately 5–10 days will be required on top of the hospitalization needed for atezolizumab plus bevacizumab treatment. There is also a possibility of adverse events due to combining hepatic arterial infusion chemotherapy using cisplatin. According to the package insert of the cisplatin formulation (IA-call® for intra-arterial administration), the main adverse events are anorexia (79.8%), nausea/vomiting (76.0%), fever (63.5%), fatigue (26.9%), leukopenia (78.8%), neutropenia (77.2%), thrombocytopenia (76.9%), increased AST (GOT) (56.7%), decreased hemoglobin (51.0%), increased ALT (GPT) (45.2%), increased serum bilirubin (36.5%), decreased serum albumin (31.7%), decreased total serum protein (29.8%), increased LDH (27.9%), increased BUN (25.0%), and increased blood creatinine (21.2%), among others.

## (3) Response to Participants When Adverse Events Occur

When an adverse event is observed, the principal investigator and subinvestigators will immediately take appropriate measures and record the event in the medical record and the case report form. If administration of the investigational drug is discontinued or treatment for the adverse event becomes necessary, the participant will be informed accordingly.

## (4) Changes to the Study Protocol and Related Documents

Information necessary for safe conduct of the clinical research will be collected and reviewed. If new safety information or other important information is obtained, the study protocol and the informed consent explanatory document will be revised as necessary. Any modification or revision of the study protocol or the informed consent document will require prior approval from the Certified Clinical Research Review Board. If the implementation plan is changed, the following documents will be submitted to the Minister of Health, Labour and Welfare: (1) Notification of changes to items in the implementation plan (Ministerial Ordinance Form 2), and (2) the implementation plan after the change.

Changes concerning the progress of the specified clinical research will be handled without delay after the change. A request for amendment will be promptly submitted to the Certified Clinical Research Review Board. After approval is obtained, the above documents will be submitted to the Minister of Health, Labour and Welfare.

If any of the following minor changes are made to the implementation plan, the details of the change will be notified to the Certified Clinical Research Review Board within 10 days after the change, and a notification form (Ministerial Ordinance Form 3) will be submitted to the Minister of Health, Labour and Welfare: (1) a change in the name of a person engaged in the specified clinical research that does not involve a change in the person engaged in the research; and (2) a change due to a change in a regional name or street address number.

## (5) Discontinuation Criteria for Individual Participants

## Response at Study Discontinuation

If the principal investigator or subinvestigator judges that continuation of the study is impossible for an individual participant for any of the reasons listed below, the study will be discontinued for that participant. If necessary, the reason for discontinuation will be explained to the participant. After discontinuation, treatment of the participant will be handled sincerely so as not to place the participant at a disadvantage.

## Discontinuation Criteria

1. The participant expresses a wish to withdraw from study participation or withdraws consent.

2. The entire study is discontinued.

3. The participant meets the discontinuation criteria for any of the study drugs.

4. Continuation of the study is judged difficult because of worsening comorbidities or other factors.

5. For any other reason, the principal investigator or subinvestigator judges that discontinuation of the study is appropriate.

## Response at Discontinuation of Study Treatment

If the principal investigator or subinvestigator judges that investigational treatment cannot be continued for an individual participant for any of the reasons listed above, the study treatment will be discontinued for that participant. If necessary, the reason for discontinuation will be explained to the participant. After treatment discontinuation, the participant will continue to be cared for sincerely so as not to be disadvantaged. Among cases in which study treatment is discontinued, participants who have received the investigational drug will continue to be observed for safety evaluation.

# 15. Methods for Storage and Disposal of Information (Including Storage Period)

The principal investigator will prepare records concerning the following matters:

1. Matters identifying the participants in this study.

2. Matters concerning medical care and examinations for participants in this study.

3. Matters concerning participation in this study.

4. Any other matters necessary to conduct this study.

Specimens and information collected in this study will be stored until 5 years have passed after completion of the study in a lockable location (at the representative institution, the Department of Gastroenterology, Okayama University Hospital; at participating institutions, locations determined by each institution), and will thereafter be discarded with due attention to personal information. Direct identifiers such as names, addresses, and dates of birth will be removed from the information retained.

The principal investigator will also store essential documents related to conduct of the study (including the study protocol, implementation plan, explanatory documents and consent documents for participants, the summary report and other documents prepared by the principal investigator under ministerial ordinances or copies thereof, documents regarding review opinions received from the Certified Clinical Research Review Board, monitoring-related documents, the participant identification code list, consent forms, copies of case report forms, and any other documents or records necessary to ensure data reliability) in a lockable location until 5 years have passed after completion of the study, and will thereafter dispose of them with due attention to personal information. Electronic information will be completely deleted, and paper materials will be shredded and discarded.

# 16. Access to Source Documents and Related Materials

In this study, source documents include the following:

1. Records concerning informed consent and information provision by investigators.

2. Medical records, clinical laboratory data, imaging films, and other records that formed the basis of the data at case registration and of the case report forms.

The principal investigator and the implementing medical institutions will provide direct access to all clinical research-related records, including source documents, for monitoring, auditing, and investigations by the Certified Clinical Research Review Board and regulatory authorities.

# 17. Periodic Reporting

The principal investigator will report the following information on the conduct of this study to the administrator of the implementing medical institution and then to the Certified Clinical Research Review Board once every year from the date on which information concerning the study was made public on jRCT, and within 2 months after the end of each such period. Standardized Form 5 (Periodic Report) and Appendix Form 3 (Periodic Report) will be used.

1. Number of participants enrolled in this study.

2. Occurrence of diseases, disorders, or other events related to this study and subsequent course.

3. Occurrence of non-compliance with ministerial ordinances or the study protocol in relation to this study and subsequent actions taken.

4. Evaluation of the safety and scientific validity of this study.

5. Matters concerning involvement of pharmaceutical manufacturers or others in this study.

In addition, within 1 month from the date on which the Certified Clinical Research Review Board issues its opinion, the following matters will be reported to the Minister of Health, Labour and Welfare using Appendix Form 3:

1. The name of the Certified Clinical Research Review Board stated in the implementation plan.

2. The judgment of the Certified Clinical Research Review Board on whether continuation of this study is appropriate.

3. The number of participants enrolled in this study.

The principal investigator will provide information on the contents of the periodic report and the committee’s approval to the responsible investigators at each site. Each site investigator will also report the above periodic report and related documents to the administrator of his or her own implementing medical institution.

# 18. Source of Funding, Conflicts of Interest Related to the Study During the Study Period, Personal Financial Interests, and Other Conflict-of-Interest Status Related to the Study

This study will be funded by Nippon Kayaku Co., Ltd. The principal investigator will declare conflicts of interest and obtain review and approval in accordance with the Guidance for Conflict of Interest Management under the Clinical Trials Act. At the time of periodic reporting, it will also be confirmed whether there have been any changes in study-related conflicts of interest or personal conflicts of interest, and such information will be reported to the Certified Clinical Research Review Board.

# 19. Public Disclosure of Information on the Study (Registration of the Study Plan and Publication of Results)

This study will be registered in the Japan Registry of Clinical Trials (jRCT). The results obtained in this study are planned to be presented through jRCT and academic meetings and to be published as a paper in a peer-reviewed academic journal.

# 20. Response to Consultations and Inquiries from Study Participants and Others

The principal investigator and subinvestigators will respond appropriately and promptly to consultations, inquiries, complaints, and other matters from study participants and related persons.

At Okayama University Hospital, the following consultation desk will be established.

## Person Responsible for the Consultation Desk

Yasuto Takeuchi

Center for Innovative Clinical Medicine, Okayama University Hospital

Address: 2-5-1 Shikata-cho, Kita-ku, Okayama 700-8558, Japan

Tel: 086-235-7219 (weekdays 9:00–17:00)

Tel: 086-235-6724 (weekday nights and holidays)

Fax: 086-225-5991

## Complaint Consultation Desk

Clinical Trial and Clinical Research Consultation Desk, General Patient Support Center, Okayama University Hospital

Contact: 086-235-7744 (weekdays 8:30–17:00)

E-mail: iscps@okayama-u.ac.jp

# 21. Procedures for Obtaining Informed Consent from a Legally Acceptable Representative

No legally acceptable representative will be used in this study.

# 22. Procedures for Obtaining Informed Assent (Including Items to Be Explained and Method of Explanation)

Not applicable to this study.

# 23. Procedures for Confirming That All Requirements for Research Conducted in an Urgent and Clear Life-Threatening Situation Are Met

Not applicable to this study.

# 24. Economic Burden on Study Participants and Others, or Honoraria, If Any

Medical expenses for treatment administered in this study will be paid through the participant’s health insurance because the treatment is within the approved insurance coverage. However, participants who receive the combination treatment with the cisplatin formulation may incur additional costs due to prolonged hospitalization or additional inpatient treatment associated with participation in the study. No honorarium will be paid for participation in this study.

# 25. Response When Diseases, Disorders, or Other Events Occur

## (1) Definition of Diseases, Disorders, and Other Events

Diseases, disorders, and other events mean diseases, disabilities, deaths, or infections suspected to be caused by implementation of the specified clinical research.

## (2) Serious Diseases, Disorders, and Other Events

Seriousness of diseases, disorders, and other events suspected to be caused by implementation of the specified clinical research is defined as follows:

1. Death or events that may lead to death.

2. Events other than item 1 that fall under any of the following:

(i) events requiring hospitalization in a medical institution for treatment or prolongation of hospitalization;

(ii) disability;

(iii) disease that may lead to disability;

(iv) events that are serious in a manner equivalent to items (i) through (iii), death, or events that may lead to death;

(v) congenital disease or anomaly in the next generation.

## (3) Causal Relationship to the Study

The principal investigator, site investigator, or subinvestigator will judge the relationship between all diseases, disabilities, deaths, or infections and implementation of the study or investigational treatment. This judgment will consider not only the temporal relationship to the start of investigational treatment but also the course of the underlying disease, comorbidities, concomitant medications, study procedures, accidents, and other external factors.

## (4) Expectedness

Expectedness of diseases or disorders will be judged on the basis of the package inserts and interview forms. If the nature, severity, or frequency of an adverse event does not match known information, it will be treated as an unknown adverse event. The expected adverse events are as follows.

1. Cisplatin for hepatic arterial administration

Overview: In a domestic clinical trial involving 104 patients with hepatocellular carcinoma, adverse reactions and abnormal laboratory values were observed in 99.0% of patients. Major events included anorexia (79.8%), nausea/vomiting (76.0%), fever (63.5%), fatigue (26.9%), leukopenia (78.8%), neutropenia (77.2%), thrombocytopenia (76.9%), increased AST (GOT) (56.7%), decreased hemoglobin (51.0%), increased ALT (GPT) (45.2%), increased serum bilirubin (36.5%), decreased serum albumin (31.7%), decreased total serum protein (29.8%), increased LDH (27.9%), increased BUN (25.0%), and increased blood creatinine (21.2%).

Serious adverse reactions:

1) Acute renal failure (frequency unknown).

2) Myelosuppression including pancytopenia: thrombocytopenia (23.2%), leukopenia (20.8%), neutropenia (18.7%), anemia (<1%), and pancytopenia (frequency unknown).

3) Thrombocytopenia (1.4%).

4) Shock and anaphylaxis (frequency unknown).

5) Hepatic dysfunction (35.4%), jaundice (<1%), and fulminant hepatitis (frequency unknown).

6) Hepatobiliary disorders (<1%).

7) Myocardial infarction (<1%), angina pectoris (frequency unknown), congestive heart failure (frequency unknown), and arrhythmia (<1%).

8) Pulmonary tuberculosis (<1%).

9) Hearing impairment (<1%).

10) Papilledema, retrobulbar optic neuritis, and cortical blindness (all frequency unknown).

11) Cerebral infarction (frequency unknown).

12) Hemolytic uremic syndrome (frequency unknown).

13) Hemolytic anemia (frequency unknown).

14) Interstitial pneumonia (frequency unknown).

15) Syndrome of inappropriate antidiuretic hormone secretion (frequency unknown).

16) Gastrointestinal bleeding, peptic ulcer, and gastrointestinal perforation (all frequency unknown).

17) Acute pancreatitis (frequency unknown).

18) Hyperglycemia (<1%) and worsening of diabetes (frequency unknown).

19) Rhabdomyolysis (frequency unknown).

2. Atezolizumab

International phase III clinical trial (IMbrave150): Among 329 patients (including 35 Japanese patients) treated with this drug and bevacizumab (genetically engineered), adverse reactions were observed in 276 patients (83.9%). Major adverse reactions (≥10%) were hypertension in 78 patients (23.7%), proteinuria in 62 (18.8%), fatigue in 50 (15.2%), increased AST in 46 (14.0%), pruritus in 43 (13.1%), infusion-related reactions in 36 (10.9%), diarrhea in 34 (10.3%), increased ALT in 34 (10.3%), and decreased appetite in 33 (10.0%).

Serious adverse reactions:

1) Interstitial lung disease (2.7%).

2) Hepatic dysfunction and hepatitis: increased AST (5.5%), increased ALT (5.5%), increased alkaline phosphatase (1.9%), increased γ-GTP (0.8%), increased bilirubin (1.4%), etc.

3) Colitis (1.2%) and severe diarrhea (1.4%).

4) Pancreatitis (0.1%).

5) Type 1 diabetes mellitus (0.1%).

6) Thyroid dysfunction: hypothyroidism (7.5%), hyperthyroidism (2.9%), thyroiditis (0.4%), etc.

7) Adrenal dysfunction: adrenal insufficiency (0.4%), acute adrenocortical insufficiency (<0.1%), etc.

8) Pituitary dysfunction: hypophysitis (0.3%), hypopituitarism (0.1%), etc.

9) Encephalitis (0.1%) and meningitis (0.1%).

10) Neurologic disorders: peripheral neuropathy (3.1%), polyneuropathy (0.2%), Guillain–Barré syndrome (0.1%), etc.

11) Myasthenia gravis (frequency unknown).

12) Severe skin disorders: erythema multiforme (0.2%), toxic epidermal necrolysis (TEN) (frequency unknown), and oculomucocutaneous syndrome / Stevens–Johnson syndrome (frequency unknown), etc.

13) Renal dysfunction: acute kidney injury (0.4%), renal failure (0.4%), tubulointerstitial nephritis (0.3%), nephritis (0.2%), etc.

14) Myositis (0.1%) and rhabdomyolysis (0.1%).

15) Myocarditis (0.1%).

16) Hemophagocytic syndrome (<0.1%).

17) Infusion reaction (2.8%).

18) Febrile neutropenia (2.8%).

3. Bevacizumab

International phase III clinical trial (YO40245 [IMbrave150]): Among 329 patients (including 35 Japanese patients) treated with this drug and atezolizumab (genetically engineered), adverse reactions were observed in 276 patients (83.9%). Major adverse reactions (≥10%) were hypertension in 78 patients (23.7%), proteinuria in 62 (18.8%), fatigue in 50 (15.2%), increased AST in 46 (14.0%), pruritus in 43 (13.1%), infusion-related reactions in 36 (10.9%), diarrhea in 34 (10.3%), increased ALT in 34 (10.3%), and decreased appetite in 33 (10.0%).

Serious adverse reactions:

1) Shock and anaphylaxis (1.9%).

2) Gastrointestinal perforation (0.9%).

3) Fistula (0.3%).

4) Delayed wound healing: because an effect on wound healing is possible, wound dehiscence (0.5%) and postoperative hemorrhage (0.4%), etc.

5) Hemorrhage (19.3%).

6) Thromboembolism: cerebral infarction (0.2%), transient ischemic attack (0.1%), angina pectoris (0.1%), myocardial infarction (<0.1%), cerebrovascular accident (frequency unknown), cerebral ischemia (frequency unknown), etc.

7) Hypertensive encephalopathy (frequency unknown) and hypertensive crisis (frequency unknown).

8) Reversible posterior leukoencephalopathy syndrome (<0.1%).

9) Nephrotic syndrome (<0.1%).

10) Myelosuppression: when used in combination with other antineoplastic agents, pancytopenia (<0.1%), neutropenia (24.5%), leukopenia (24.3%), anemia (8.7%), and thrombocytopenia (10.4%) may occur.

11) Infection (10.0%).

12) Congestive heart failure (<0.1%).

13) Interstitial pneumonia (0.4%).

14) Thrombotic microangiopathy (frequency unknown).

15) Arterial dissection (<0.1%).

## (5) Outcome

The principal investigator and others will provide appropriate management and treatment for all diseases, disorders, and other events that occur, and will perform follow-up until symptoms disappear, laboratory values return to the reference range or pre-treatment values, or the principal investigator judges that no further medical follow-up is necessary. Outcomes of adverse events will be classified as follows: 1) recovered (disappearance or recovery of symptoms, findings, or clinical laboratory values); 2) improved (amelioration of the event); 3) not recovered (unchanged to worsened); 4) death; or 5) unknown.

## (6) Responsibilities of the Principal Investigator, Responsible Investigators, and Subinvestigators (Reporting of Serious Diseases, Disorders, and Other Events)

1. When the principal investigator, site investigator, or subinvestigator becomes aware of the occurrence of a serious disease, disorder, or other event during implementation of the specified clinical research, he or she must take the necessary measures, including explanation to the participant and treatment.

2. When a responsible investigator becomes aware of the occurrence of a disease, disorder, or other event, he or she shall promptly report it to the administrator of the implementing medical institution and then to the principal investigator.

3. When the principal investigator becomes aware of the occurrence of a disease, disorder, or other event, he or she shall report it to the administrator of the implementing medical institution and, in accordance with the ministerial ordinances, report it using Standardized Form 8 to the Certified Clinical Research Review Board stated in the implementation plan for the specified clinical research.

4. When the principal investigator becomes aware of matters concerning the occurrence of diseases, disorders, or other events suspected to be caused by implementation of the specified clinical research and prescribed by ministerial ordinances (see table in the original protocol), he or she shall report them to the Minister of Health, Labour and Welfare using Appendix Form 2-1 (drugs) in accordance with the ministerial ordinances.

The corresponding table is omitted in this English text-only version.

# 26. Compensation for Health Damage and Details Thereof

Because this study uses medicinal products within the approved indications and dosage range, clinical research insurance will not be purchased. Should health damage occur as a result of participation in this study, the participant’s own health insurance will be used to provide care.

# 27. Reporting of Non-Compliance

If a responsible investigator becomes aware that the clinical research is in a state not conforming to laws, regulations, or the study protocol (“non-compliance”), he or she shall promptly report this to the administrator of the implementing medical institution and to the principal investigator. If a subinvestigator becomes aware of non-compliance, he or she shall promptly report it to the responsible investigator. If particularly serious non-compliance becomes known, the principal investigator shall promptly seek the opinion of the Certified Clinical Research Review Board.

“Serious non-compliance” means non-compliance that affects the human rights or safety of study participants or the reliability of the progress or results of the study. Examples include failure to comply with the inclusion/exclusion criteria, discontinuation criteria, or prohibited concomitant therapies; however, deviations from the protocol made to avoid an urgent danger to a participant or for other medically unavoidable reasons are not included. When serious non-compliance occurs, the responsible investigator shall take measures to prevent recurrence, inform subinvestigators and other persons engaged in the study, and ensure thorough implementation of those measures. At the time of periodic reporting to the committee, the occurrence of non-compliance with laws, regulations, or the study protocol and the subsequent responses must be reported.

# 28. Completion, Suspension, or Discontinuation of the Study

## (1) Completion of the Study

When the period for collecting data related to the primary endpoint described in the study protocol has ended, the principal investigator shall, in principle, prepare a primary endpoint report within 1 year. When the period for collecting data related to all endpoints described as matters concerning the content of the clinical research has ended, the principal investigator shall, in principle, prepare a summary report and its synopsis within 1 year and submit them to the Certified Clinical Research Review Board.

If the time when the primary endpoint report and the summary report must be prepared coincides, preparation of the summary report shall be deemed to constitute preparation of the primary endpoint report.

The principal investigator shall submit the synopsis of the primary endpoint report or the summary report to the Minister of Health, Labour and Welfare within 1 month from the date on which the Certified Clinical Research Review Board issues its opinion, and shall publish it on jRCT. When submitting the synopsis of the summary report to the Minister of Health, Labour and Welfare, the study protocol and explanatory document shall also be submitted.

When the principal investigator prepares the primary endpoint report or summary report and its synopsis, he or she shall submit them to the head of the implementing medical institution without delay. When public disclosure has been made, he or she shall promptly report this to the administrator of the implementing medical institution. When the synopsis of the primary endpoint report or summary report has been made public, the principal investigator shall provide that information to the other responsible investigators. Upon receiving the information, each responsible investigator shall promptly report its content to the administrator of the affiliated medical institution.

## (2) Discontinuation of the Study

The principal investigator will consider whether continuation of the study is possible when any of the following apply:

1. Important information is obtained regarding the quality, safety, or efficacy of the medicinal products used in this study.

2. Recruitment of participants is difficult and it is judged extremely difficult to achieve the planned sample size.

3. Before the planned sample size or planned period is reached, the objective of the trial has been achieved.

4. The Certified Clinical Research Review Board instructs changes to the implementation plan or related matters and it is judged difficult to accept them.

If the Certified Clinical Research Review Board recommends or instructs discontinuation, the study will be discontinued.

When discontinuation of the study is decided, the principal investigator shall notify the Certified Clinical Research Review Board stated in the implementation plan within 10 days from the date of discontinuation using Standardized Form 11 and shall also notify the Minister of Health, Labour and Welfare using Form 4. Even after submitting the discontinuation notice, the principal investigator shall continue disease/event reporting, periodic reporting, and other required reporting until the clinical research is completed. If changes related to the progress of the specified clinical research occur before its completion, a notification of changes to the implementation plan shall be submitted.

# 29. Handling of Study Results When Important Findings Concerning Participants’ Health or Genetic Characteristics May Be Obtained (Including Incidental Findings)

If important findings concerning the participant’s health or other important findings are obtained during the study, the participant may be informed of such findings.

# 30. Outsourcing of Part of the Study-Related Work, If Any, and Supervision of the Contractor

No study-related work will be outsourced to another institution.

# 31. Possibility of Using Samples/Information Obtained in This Study for Future Research

Information obtained in this study may be used for future research; however, in such cases approval from the ethics committee will be obtained.

# 32. Monitoring and Audit Structure and Procedures

## (1) Monitoring

Monitoring will be conducted in this study. Detailed procedures will be specified separately in the monitoring plan.

## (2) Audit

No audit will be conducted in this study.

# 33. Ownership of Intellectual Property Rights and Property

No intellectual property rights or similar property interests are expected to arise from this study.

# 34. Reference Materials and Bibliography

1) Center for Cancer Control and Information Services, National Cancer Center Japan. Cancer mortality data based on vital statistics. 2018.

2) Japan Society of Hepatology, ed. Clinical Practice Guidelines for Hepatocellular Carcinoma, 2017 revised edition. Kanehara Publishing; 2020.

3) Kondo M, Morimoto M, Kobayashi S, Ohkawa S, Hidaka H, Nakazawa T, et al. Randomized, phase II trial of sequential hepatic arterial infusion chemotherapy and sorafenib versus sorafenib alone as initial therapy for advanced hepatocellular carcinoma: SCOOP-2 trial. BMC Cancer. 2019;19(1):954.

4) Ikeda M, Shimizu S, Sato T, Morimoto M, Kojima Y, Inaba Y, et al. Sorafenib plus hepatic arterial infusion chemotherapy with cisplatin versus sorafenib for advanced hepatocellular carcinoma: randomized phase II trial. Ann Oncol. 2016;27:2090–6.

5) Finn RS, et al. Atezolizumab plus Bevacizumab in Unresectable Hepatocellular Carcinoma. N Engl J Med. 2020;382(20):1894–1905.

6) Sandler A. Paclitaxel–Carboplatin Alone or with Bevacizumab for Non–Small-Cell Lung Cancer. N Engl J Med. 2006;355:2542–2550.

7) Tewari KS, et al. Bevacizumab for advanced cervical cancer: final overall survival and adverse event analysis of a randomized, controlled, open-label, phase 3 trial (Gynecologic Oncology Group 240). Lancet. 2017;390:1654–1663.

8) Botrel TEA, et al. Efficacy of bevacizumab plus chemotherapy compared to chemotherapy alone in previously untreated locally advanced or metastatic non-small cell lung cancer: systematic review and meta-analysis. Lung Cancer. 2011;74:89–97.
